# Comparative Mitogenomics Reveals Mitochondrial Diversity of *Haemonchus contortus*, a Major Gastrointestinal Parasite of Ruminants

**DOI:** 10.3390/vetsci13101018

**Published:** 2026-09-26

**Authors:** Mashal Khalid, Ghulam Narjis, Noorah Saleh Al-Sowayan, Sabika Firasat, Iffat Naz, Kiran Afshan

**Affiliations:** 1 Department of Zoology, Faculty of Biological Sciences, Quaid-i-Azam University, Federal Islamabad, Islamabad 45320, Pakistan; mashal.khalid444@gmail.com (M.K.); sabika.firasat@qau.edu.pk (S.F.); 2 Department of Statistics, Rawalpindi Women University, 6th Road, Satellite Town, Rawalpindi 46300, Pakistan; ghulam.narjis@rwu.edu.pk; 3 Department of Biology, College of Science, Qassim University, Buraydah 51452, Saudi Arabia; nsaoiean@qu.edu.sa

**Keywords:** *Haemonchus contortus*, molecular evolution, animals, mt genome, Pakistan

## Abstract

*Haemonchus contortus* is a highly pathogenic hematophagous gastrointestinal nematode and one of the most important constraints to small-ruminant health and productivity worldwide, causing anemia, reduced weight gain, decreased milk production, and substantial economic losses. Understanding its genetic diversity and population structure is essential for improving parasite surveillance and informing sustainable control strategies. In this study, individual *H. contortus* larvae collected from Pakistani livestock were subjected to whole-genome sequencing, and complete mitochondrial (mt) genomes were assembled and analyzed using comparative mitogenomics, phylogenetic reconstruction, pairwise genetic distance estimation, and haplotype network analyses. Forty-one *H. contortus* larvae were successfully characterized for their mitochondrial genomes. The first complete mitochondrial genome of Pakistani *H. contortus* was 14,002 bp in length and comprised 12 protein-coding genes, 22 transfer RNA genes, and two ribosomal RNA genes, with a marked AT content of 78.37%. Mitochondrial genome organization and gene order were highly conserved relative to published *H. contortus* mitogenomes. Comparative analyses resolved the Pakistani isolates into two well-supported mitochondrial lineages, while maximum-likelihood phylogenetic analysis clustered all isolates within a robust *H. contortus* clade clearly distinct from *H. placei* (bootstrap support, 97–100%). Pairwise genetic distances among Pakistani isolates ranged from 0.00 to 0.05, indicating pronounced nucleotide divergence. Haplotype network analysis identified numerous unique haplotypes together with globally shared haplotypes, supporting substantial mitochondrial diversity. Collectively, these findings provide the first complete mitogenomic resource for *H. contortus* from Pakistan and advance our understanding of the genetic structure of this economically important veterinary parasite. The generated genomic resource will support molecular epidemiology, population genetic studies, veterinary parasite surveillance, and future investigations of parasite dissemination and anthelmintic resistance in ruminant production systems.

## Simple Summary

*Haemonchus contortus* is a blood-feeding gastrointestinal nematode that causes anemia, poor growth, and reduced milk yield in sheep and goats, resulting in substantial economic losses to livestock producers. Characterizing parasite genetic diversity is essential for effective surveillance and control. Here, we generated complete mitochondrial genomes from 41 individual *H. contortus* larvae collected from Pakistani livestock, providing the first such resource for the country. Genome organization was highly conserved relative to published isolates, yet comparative analyses revealed two distinct mitochondrial lineages and pronounced nucleotide diversity, including haplotypes unique to Pakistan alongside others shared globally. These findings show that Pakistani *H. contortus* populations are genetically diverse and connected to the global parasite gene pool, offering a valuable resource for future epidemiological surveillance and anthelmintic resistance studies.

## 1. Introduction

Haemonchosis, a parasitic disease caused by the voracious blood suckers of the genus *Haemonchus*, leads to the death of severely afflicted animals [1,2,3]. More than a dozen distinct hematophagous species that infect a wide variety of artiodactyl hosts are found in the genus *Haemonchus* [4,5,6]. Of these, *H. contortus* is the most prevalent species contributing to major financial losses of the livestock industry [7,8,9,10]. Due to the huge economic threat that haemonchosis poses to livestock production [11], accurate identification of these closely related roundworms is critical to their management and control.

The characterization of *Haemonchus* species is controversial with regard to host preferences and morphology [12]. Strict host specificity between these species is difficult to assert, as on shared pastures, *H. contortus* predominantly causes disease in small ruminants yet can infect cattle, while *H. placei* primarily infects cattle but is also found in small ruminants [13]. Moreover, relying solely on morphometry is inadequate, as adult worms’ morphological traits are considered to be influenced by a variety of factors, including host immunity [14], host gender [15], host feeding patterns [16], host variant [17] and concurrent parasitic infection [12,18]. Consequently, the classification of *Haemonchus* species based entirely on host preferences and physical characteristics remains debatable and necessitates the application of molecular approaches to identify unique and intraspecific mutations linked to their geographic distributions [12,19].

Understanding the genetic diversity and evolutionary trends of *Haemonchus* spp. is essential for species delineation, for the establishment of realistic control programs, and to circumvent the challenges related to anti-helminthic resistance. Significant advancements in genotyping and bioinformatics have allowed the sequencing and compilation of genomic and multi-omics data sets for *H. contortus* [20,21,22]. New trends in sequencing technologies are revolutionizing the process of decoding the organelle genome [23]. The eukaryotic cell’s powerhouse, the mitochondrion, is a subcellular organelle that performs significant biochemical functions and possesses a distinct genome from the nuclear genome known as the mitochondrial genome [24]. Owing to its rapid evolution, high mutation rates, maternal inheritance and simple organization, the huge and extremely diverse mt genomes of parasitic nematodes serve as an invaluable repository of genetic markers for resolving phylogenetic, evolutionary, taxonomical and speciation research [25]. Complete mt genome sequences frequently serve as useful genetic markers to distinguish cryptic species, species complexes, and synonymous species in parasites, especially for *Trichostrongyloidea* [26,27,28].

The mitogenome of *H. contortus* strains sequenced globally includes the McMaster strain from Australia [29]; MHco3(ISE). N1 from the United Kingdom [30]; NZ_Hco_NP from New Zealand [31]; Haecon-5 from Australia [32]; and strain ChinHco from China [25]. In Pakistan, parasitism is one of the major menaces for livestock, causing obstacles in the development of a profitable livestock industry [33]. Geoclimatic conditions and poor awareness among livestock farmers contribute to a conducive environment for the development and growth of parasites [34]. High prevalence of haemonchosis has been reported from all provinces of the country [11,35,36,37,38], costing significant economic losses.

Despite the global veterinary importance of *Haemonchus contortus*, complete mitochondrial genomic data from Pakistani populations remain unavailable, limiting molecular investigations of parasite diversity and population structure. Therefore, this study aimed to generate and characterize the first complete mitochondrial genomes of Pakistani *H. contortus* and to investigate their genetic diversity, phylogenetic relationships, genetic divergence, and haplotype diversity through comparative mitogenomic analyses.

## 2. Materials and Methods

### 2.1. Fecal Sampling and Larval Culture

Fresh fecal material was collected directly from the rectum of slaughtered goats (n = 150) and cattle (n = 100) using sterile gloves at four abattoirs (Barakahu, Sihala, Saidpur, Rawalpindi) in the Rawalpindi district of Pakistan (Figure S1). For parasite recovery, fecal material was thoroughly homogenized and incubated at room temperature for up to seven days. This culture method facilitated the development of infective third-stage (L3) larvae. Morphological identification of recovered larvae as belonging to the genus *Haemonchus* was performed using established diagnostic criteria. Using an ocular micrometer, larvae of the genus *Haemonchus* were identified and distinguished from other nematodes based on the sheath tail length [39]. Larvae were subsequently rinsed with distilled water and preserved in 70% ethanol for downstream molecular work.

### 2.2. Single-Larva DNA Extraction

Genomic DNA was isolated from individual larvae using a previously described protocol [40] with minor handling modifications. Briefly, ethanol-fixed larvae were washed to eliminate residual preservative and transferred into 96-well plates containing 50 µL TE buffer. Plates were stored at −80 °C prior to lysis. Cell disruption was carried out by adding 70 µL of lysis solution composed of 200 mM NaCl, 100 mM Tris-HCl (pH 8.5), 50 mM EDTA (pH 8.0), 0.5% SDS, 0.9 mg/mL Proteinase K, and 45 mM DTT. Samples were incubated at 60 °C for 2 h to ensure complete digestion, followed by heat inactivation of Proteinase K at 85 °C for 15 min. Resulting lysates were stored at −80 °C until further processing.

### 2.3. Library Preparation and Whole Genome Sequencing

Sequencing libraries were constructed from single-larva DNA extracts using the NEB Next Ultra II FS DNA Library Prep Kit (New England Biolabs, Ipswich, MA, USA) (half reaction conditions). Input DNA (0.1–5 ng) was enzymatically fragmented at 37 °C for 8 min, followed by end repair, adapter ligation, and indexing. Libraries were amplified through 13 PCR cycles and purified using AMPure XP beads (Beckman Coulter, Brea, CA, USA). After indexing, libraries were pooled and evaluated for quality and concentration using Qubit fluorometry (Thermo Fisher Scientific, Waltham, MA, USA), Agilent Bioanalyzer (Agilent Technologies, Santa Clara, CA, USA), and qPCR-based quantification. Sequencing was performed on an Illumina NovaSeq 6000 platform using paired-end 150 bp chemistry (2 × 150 bp) with an S1 flow cell.

### 2.4. Bioinformatic and Sequence Analyses

Out of 60 confirmed microscopically positive fecal samples, 2–3 larvae per sample were randomly selected for genomic sequencing. Species identity was validated through comparison with mitochondrial genome sequences. Raw sequencing reads were aligned to the reference mitochondrial genome of *Haemonchus contortus* (NC_010383; NCBI) using Bowtie2 (v2.3.5.1). Alignment files were processed using SAMtools (v1.9) to obtain mapping statistics, sequencing depth, and genome coverage estimates.

Mitochondrial genomes from 41 individual larvae were successfully reconstructed and subjected to multiple sequence alignment using MAFFT v7. Genome features, including nucleotide composition, genome size, and AT/GC content, were analyzed using MEGA12 and supplementary nucleotide analysis tools, DNA stats (https://www.bioinformatics.org/sms2/dna_stats.html, accessed on 10 July 2026). Multiple sequence alignments for the 12 protein-coding genes were established using MEGA12 to identify the variable sites. To ensure reliability, rare variants observed fewer than 3 times across samples were not included in the analysis (Tables S1–S12). Moreover, the percent identity of each individual mt sample with the previously reported *H. contortus* mt genome mined from the NCBI GenBank was determined (Table S13).

For downstream analysis and reporting, sample Pk18_02 was selected as the representative mitogenome due to its wholeness, query coverage and close similarity (98.47% identity) to the published reference genome (CP035799.1, https://www.ncbi.nlm.nih.gov/nucleotide/CP035799.1?report=genbank&log$=nucltop&blast_rank=1&RID=7PBUD9X3015, accessed on 10 July 2026) (Table S13). The length and position of each gene in the mitochondrial genome of larvae (Pk18_02) were confirmed manually by comparing with the reference sequence from NCBI using BioEdit software (https://bioedit.software.informer.com/7.2/, accessed on 10 July 2026). Additionally, BLAST (https://blast.ncbi.nlm.nih.gov/Blast.cgi, accessed on 10 July 2026) searches were performed to check for precise gene boundaries. The mitochondrial genome map of the sample Pk18_02 was constructed using GenomeVx (http://wolfe.ucd.ie/GenomeVx/, accessed on 10 July 2026). Nucleotide composition of each gene was calculated using DNA stats. The nucleotide diversity of the present study’s strain Pk18_02 was compared with the previously reported mt strains of *H. contortus*.

Maximum likelihood tree estimation was carried out utilizing RaxML version 8.1.15, applying a general time-reversible gamma model of substitution rate heterogeneity, and rapid bootstrapping with 100 replicates. The resulting tree was visualized as a bipartition file using the ITOL (https://itol.embl.de/, accessed on 10 July 2026) program. Reference sequences of the mt genome of other nematodes were also downloaded from the NCBI database. To describe the phylogenetic relationship of *H. contortus* with other nematode genera, a maximum likelihood tree under the Kimura-2-parameter model of nucleotide substitution with 1000 bootstrap replicates was generated using MEGA12. *T. canis* was used as the outgroup to root the tree (Table S14). Pairwise distance analyses were conducted using the Maximum Composite Likelihood model (Table S15). The analytical procedure encompassed 55 coded nucleotide sequences. The complete deletion option was applied to eliminate positions containing gaps and missing data.

A median-joining haplotype network of the *H. contortus* mitochondrial genome was generated using popart v.1.7 (https://popart.maths.otago.ac.nz, accessed on 10 July 2026). Sequences with the same nucleotide composition were regarded as a single haplotype (PK18_02, PK_18_03). The haplotype network included all successfully sequenced study isolates (41) together with the corresponding homologous full-length mt-DNA of *H. contortus* retrieved from GenBank. All newly generated gene sequences were submitted and are accessible at Mendeley Data, doi: 10.17632/btnvv54mnp.1.

## 3. Results

A total of 60 fecal samples from a population of 150 goats and 100 cattle hosts were found microscopically positive for *Haemonchus*. The genetic diversity of *Haemonchus contortus* was investigated by analyzing the whole mt-genome sequence of larvae cultured from these 60 samples. By employing Illumina technology, 41 larvae of *H. contortus* were successfully characterized for the mitochondrial genome. All sequences were complete and provided insight into the target genome. Each sequence comprised a total of 36 genes, of which 12 genes were protein-coding genes (PCGs), 22 were tRNA genes, and 2 were rRNA genes. Besides these, an AT-rich region was present in each individual genome. Sequence length, nucleotide percentage, AT richness and GC content of each sample are given in Table 1.

### 3.1. Sequence Analysis

The comparison of the protein-coding regions of the present study’s sequences revealed high intraspecific diversity. Comparative analysis showed that the most variable gene among the PCGs was Nad6 (13.6%) and the most conserved one was Cox3 (7.4%) (Table 2). The detailed intraspecific analysis of each coding gene is given in Tables S1–S12.

Among the previously reported strains, the present study’s mt sequences showed the highest similarity with the *H. contortus* strain reported from New Zealand (CP035799.1). The percent identity of each sample with the already published strain is given in Table S13.

### 3.2. Mitogenome Features

Pk 18_02 was selected as the representative mitochondrial genome for reporting. The total length of the present study’s *H. contortus* (PK 18_02) mitochondrial genome was 14,002 bp. The overall base composition was 33.21% for A, 6.42% for C, 15.20% for G and 45.16% for T. The AT richness and GC content of the present study’s *H. contortus* (Pk18_02) were 78.37% and 21.62%, respectively. The mitochondrial genome of the present study, *H. contortus* (PK 18_02), was compared to the already reported strains (Table 3).

#### 3.2.1. Mitochondrial Genome Variability of Pk (18_02)

A high degree of consistency was observed among the present study’s isolate and already reported mitogenomes, indicating reliable and reproducible results (Table 3). To analyze the nucleotide diversity, the sequences of protein-coding genes obtained were compared with those of previously reported *H. contortus* genomes. The comparative analysis enabled the identification of sequence polymorphisms and provided insights into the extent of genetic divergence among the Pk (18_02) and reference isolates. The Nad6 gene exhibited high genetic diversity with 26 (5.90%), 13 (2.95%) and 23 (5.22%) alterations in CP149429.1, CP035799.1 and NC_010383.2 respectively. The genes Nad3, Nad4, Nad4l and Cyt b of the present study’s isolate showed high sequence similarity with the strain reported from New Zealand (CP035799.1), indicating a conserved evolutionary trend across these mitochondrial loci (Table 4).

**Figure 1:**
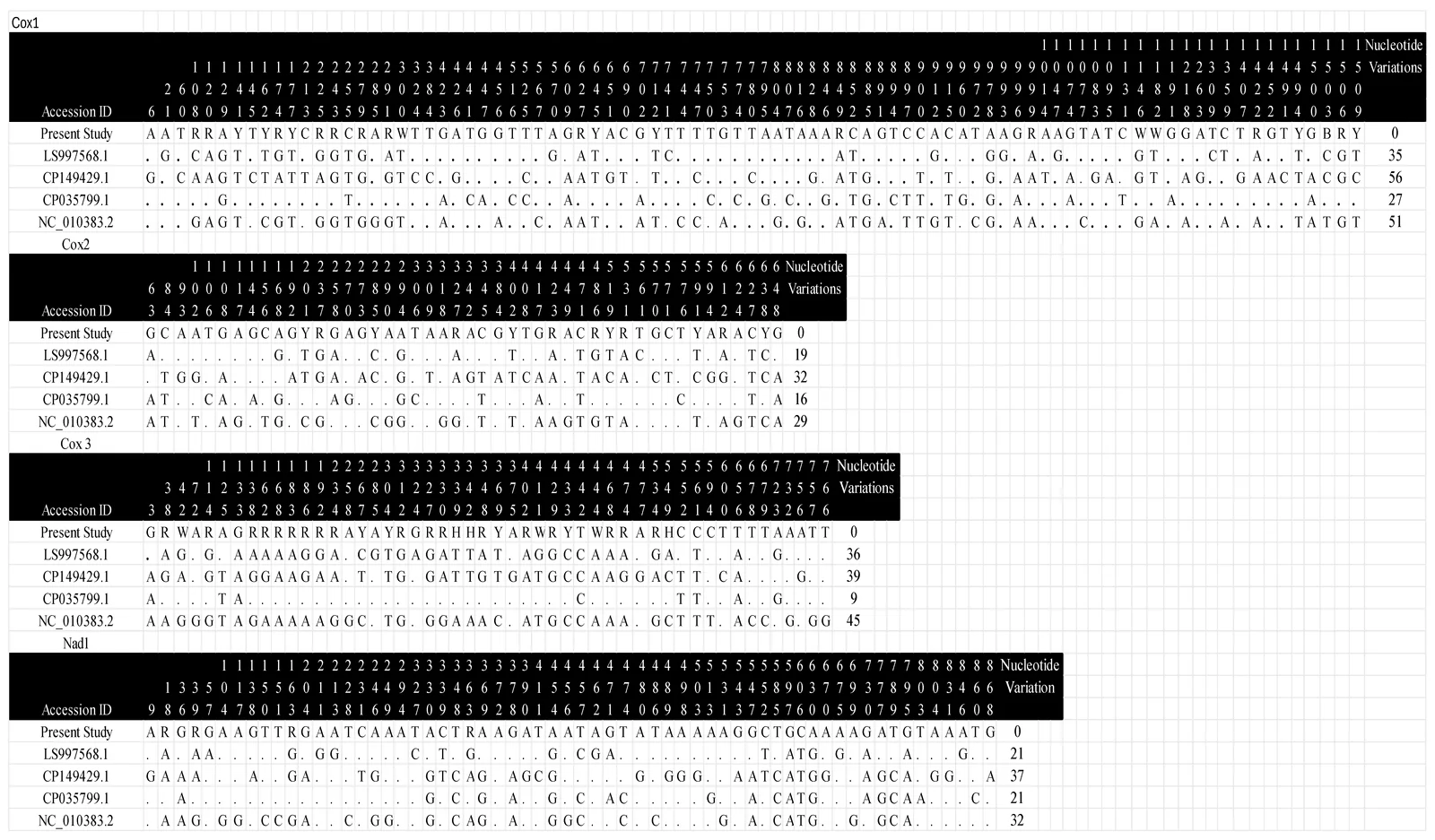

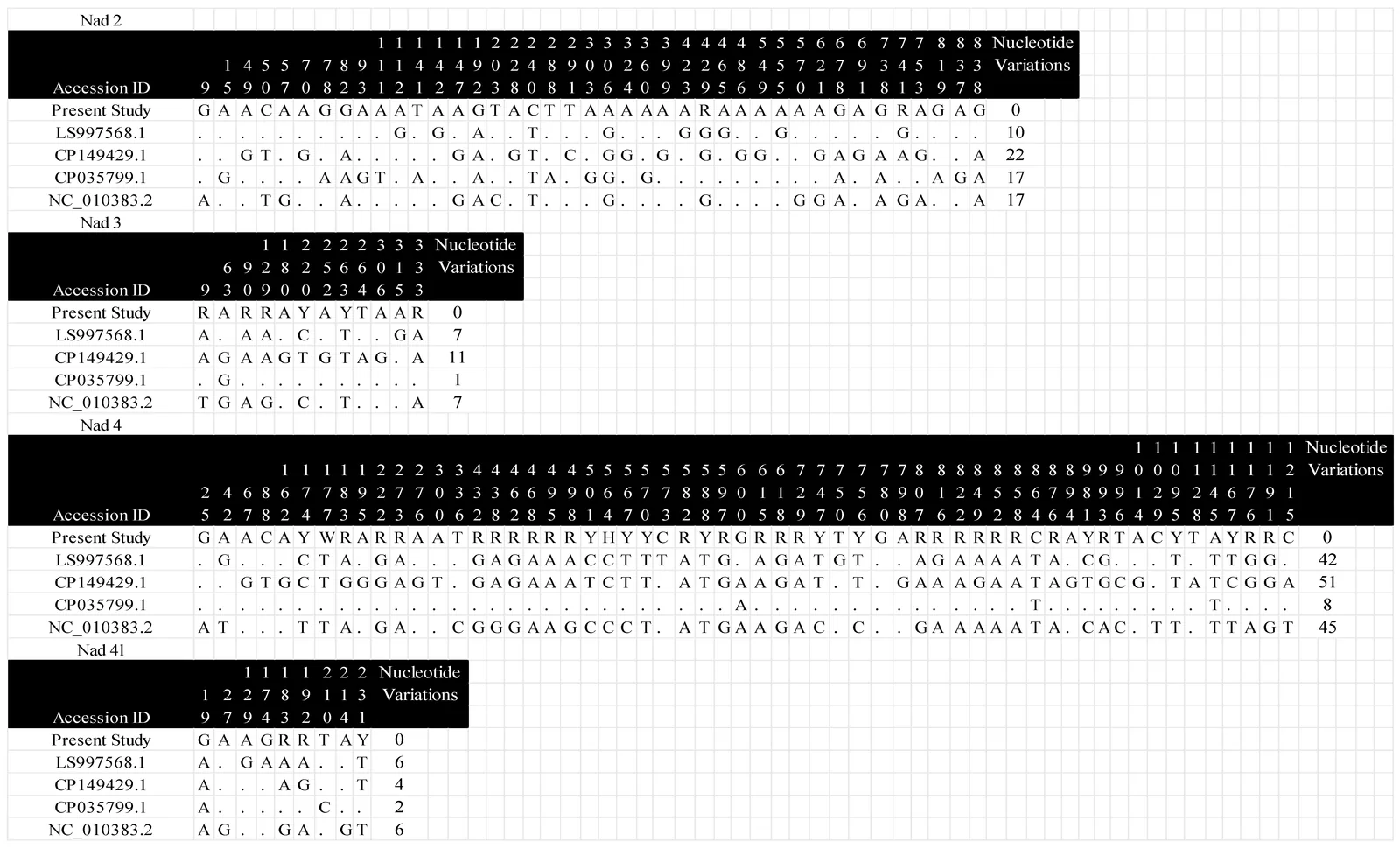

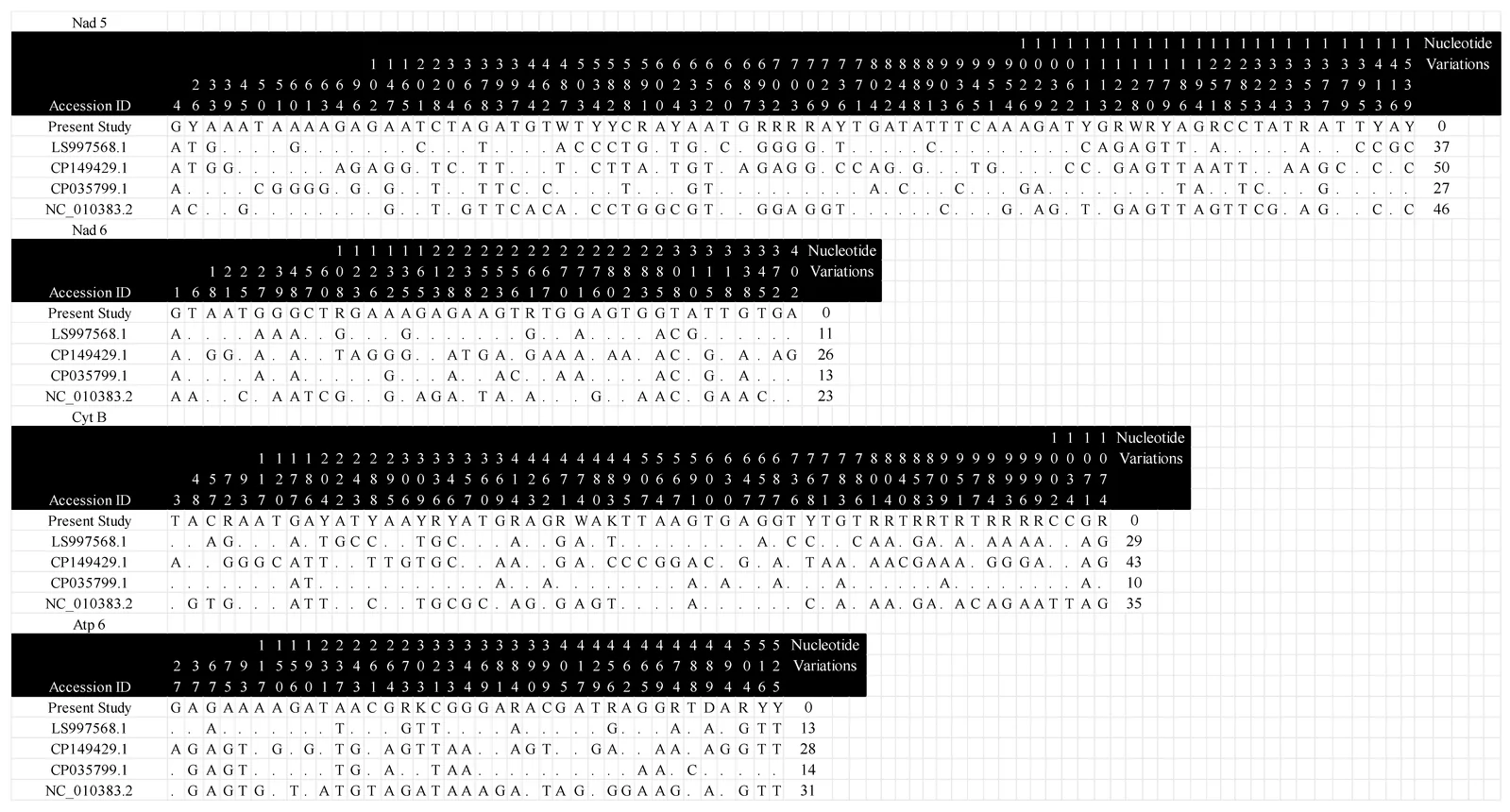
Nucleotide variability in mitochondrial genes of *H. contortus* Pk18_02 isolate with GenBank reference sequences. Dots indicate similarity.

The detailed sequence analysis for each coding gene is presented in Figure 1. Ambiguous nucleotide bases observed in the present study’s sequences corresponded to the previously published GenBank strain (Accession No: CP035799.1), reflecting natural intraspecific genetic diversity.

#### 3.2.2. Genome Orientation

**Figure 2:**
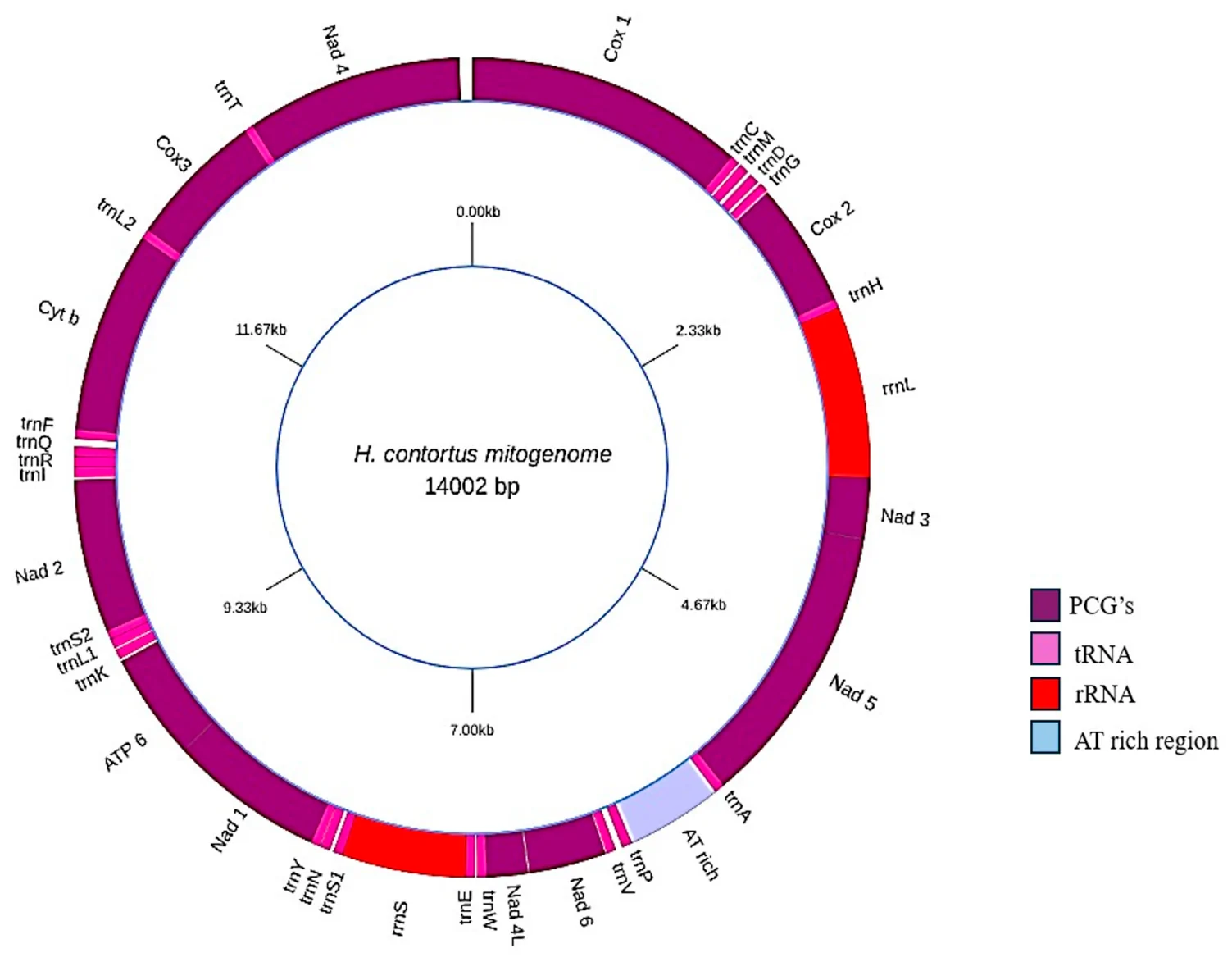
Complete circular mitochondrial genome map for *Haemonchus contortus*.

The mitochondrial genome of *H. contortus* (Pk18_02) consists of 36 genes (12 protein-coding genes, 22 tRNA genes, two rRNA genes, and an AT-rich region (Figure 2). The order of genes observed in the present study of the *H. contortus* mitogenome is identical to that of previously reported strains of the parasite.

#### 3.2.3. Comparative Mitogenomics of Pk (18_02) with Other Strains

The length, start/stop position, start/stop codon and nucleotide composition of each individual gene are given in Table 5. All protein-coding genes consist of high AT content. The protein-coding genes with the highest AT (83.4%) content were Nad2, Nad4l and the gene with the lowest AT (70.16%) content was Cox1. The predominant initiation codon for the *H. contortus* mt PCGs was ATN (ATA/ATT), while the most common termination codon was TAA for 11 genes (Table 5).

### 3.3. Phylogenomic Analysis

**Figure 3:**
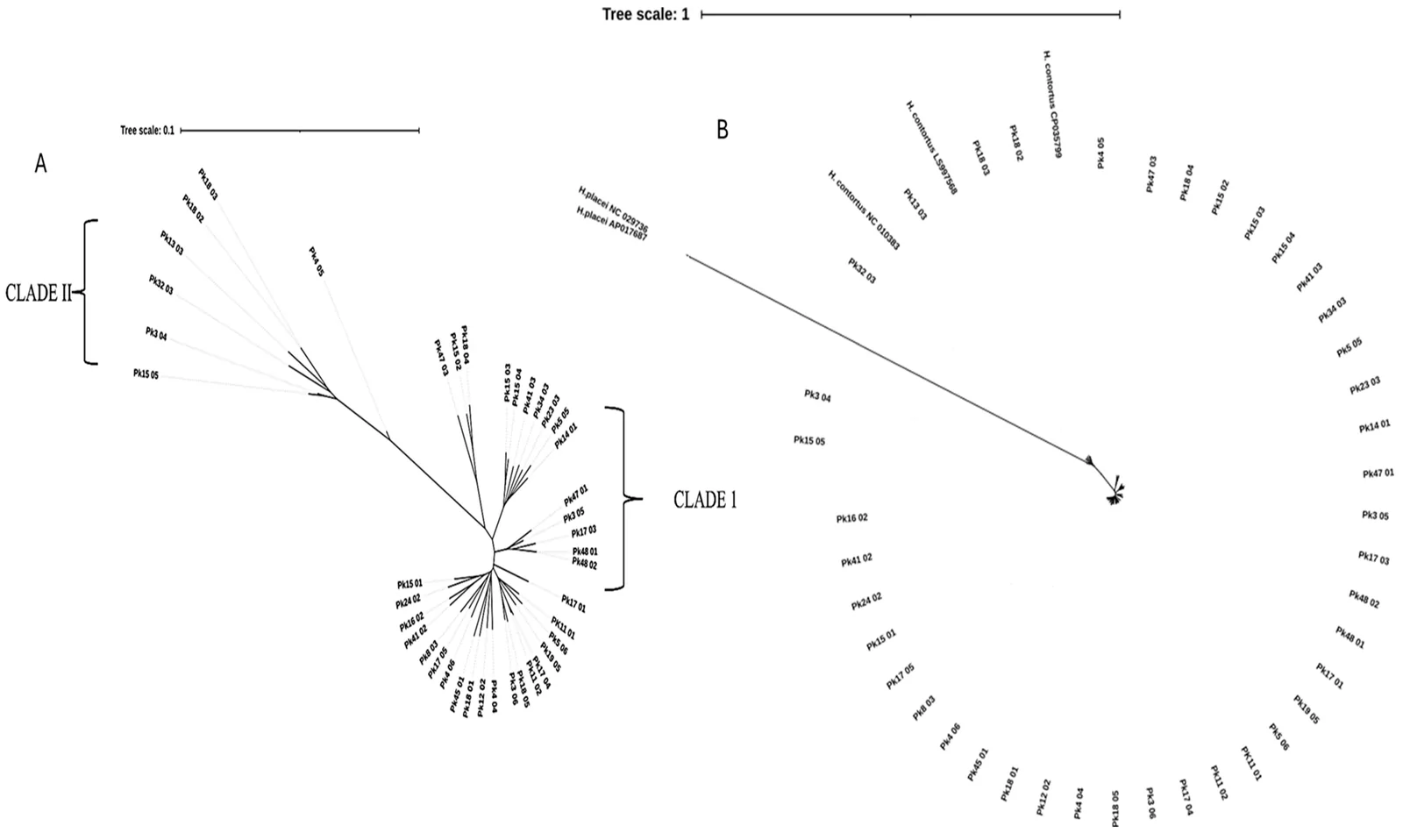
(**A**) Maximum likelihood unrooted tree of present study’s *H. contortus* genome (**B**) with reference sequences of *H. contortus* and *H. placei* from NCBI GenBanK.

The unrooted phylogenetic tree based on mitochondrial genome loci of *H. contortus* shows a clear separation of the analyzed isolates into two distinct genetic groupings (Figure S2), designated as Clade I and Clade II (Figure 3A), indicating structured mitochondrial diversity within the study population. Clade I comprises the majority of isolates and is characterized by short internal branch lengths and tight clustering, suggesting low genetic divergence. In contrast, Clade II includes a smaller number of isolates that are separated from Clade I by comparatively longer branch lengths, indicating greater mitochondrial divergence and the presence of a more differentiated lineage within the population. The observed variation in branch lengths and clustering patterns clearly demonstrates intraspecific mitochondrial variation in *H. contortus*, highlighting the effectiveness of mitochondrial genome loci in resolving genetic structure in the present study.

In the phylogenetic reconstruction (Figure 3B), the Pakistani isolates cluster closely with the *H. contortus* reference genome, confirming their species-level identity and demonstrating high mitochondrial sequence similarity. In contrast, the *H. placei* reference genome is positioned separately from the main cluster, showing clear genetic distinction from *H. contortus* and supporting its validity as a closely related but distinct species. Overall, the comparative placement of Pakistani isolates relative to the *H. contortus* and *H. placei* reference genomes highlights intraspecific variation within *H. contortus* and a clear interspecific boundary between the two *Haemonchus* species at the mitochondrial genome level.

**Figure 4:**
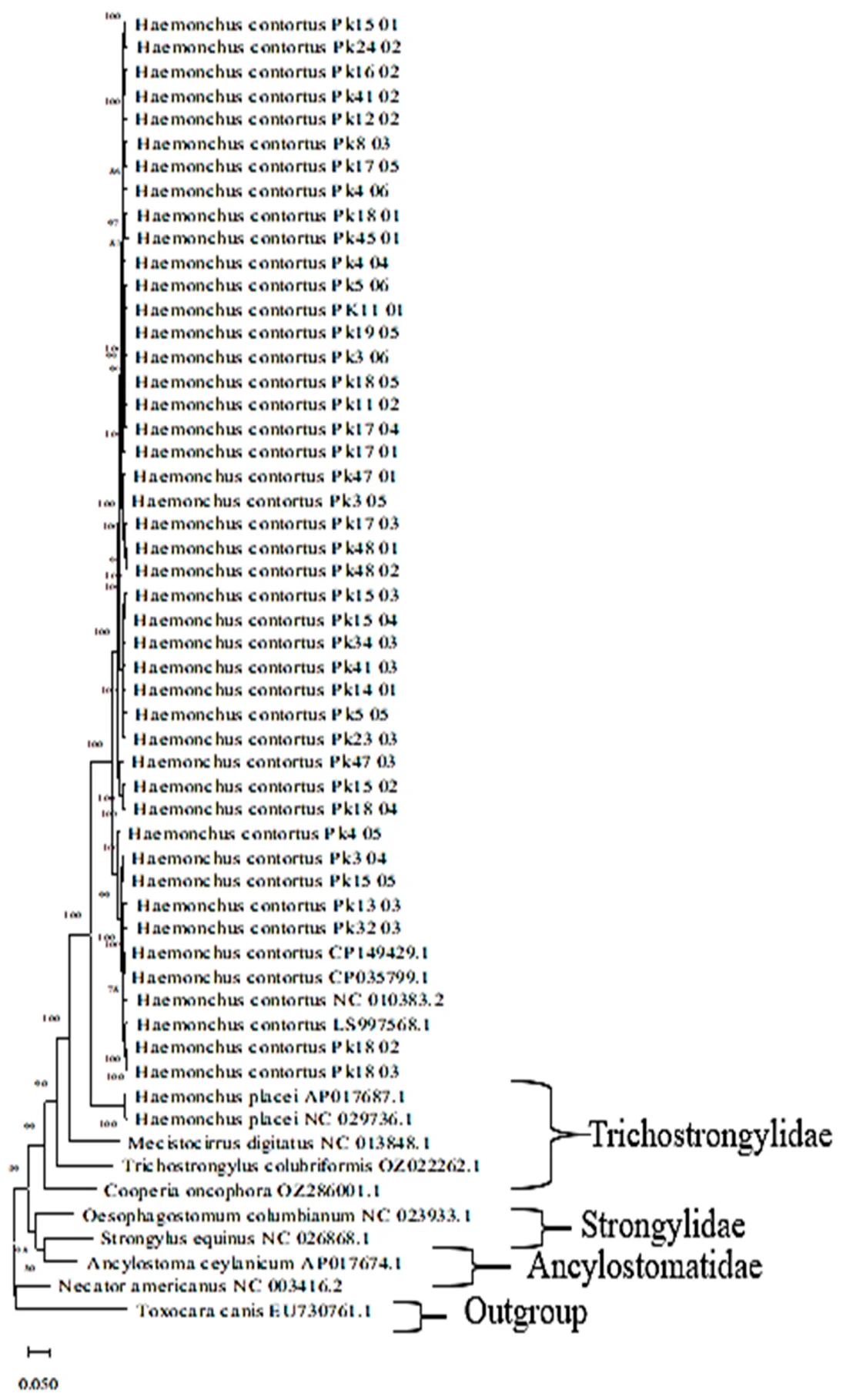
The phylogeny was inferred using the maximum likelihood method and the tree with the highest log-likelihood (−150,689.87) is shown. The percentage of replicate trees in which the associated taxa clustered together (1000 replicates) is shown next to the branches.

The maximum-likelihood phylogenetic analysis (Figure 4) based on mitochondrial genome sequences resolved the Pakistani *Haemonchus contortus* isolates into a single, well-supported clade together with established *H. contortus* reference genomes, with most internal nodes supported by high bootstrap values (≥97–100%), indicating strong confidence in the inferred relationships. In comparison, *Haemonchus placei* reference sequences formed a distinct sister clade that was clearly separated from *H. contortus* with strong bootstrap support (≈99–100%), demonstrating reliable species-level differentiation despite their close phylogenetic relationship. At higher taxonomic levels, members of *Trichostrongylidae*, *Strongylidae*, and *Ancylostomatidae* clustered into their respective lineages with moderate to high bootstrap support, whereas deeper nodes showed relatively lower values, consistent with greater evolutionary divergence and reduced phylogenetic resolution of mitochondrial markers at broader taxonomic scales. The outgroup (*Toxocara canis*) was robustly separated from all ingroup taxa, supporting the overall tree topology.

Pairwise genetic distance analysis under the maximum composite likelihood model (MCL) (Table S15) revealed low levels of divergence (0.00–0.05) among Pakistani *H. contortus* isolates, indicating a high degree of genetic relatedness within the local population. Likewise, comparisons between the Pakistani isolate (PK3_04) and reference *H. contortus* sequences retrieved from GenBank showed low genetic distances (0.01–0.02), confirming their conspecific status and shared evolutionary ancestry. In contrast, genetic distances between Pakistani *H. contortus* isolates and *H. placei* were substantially higher (0.16), reflecting clear interspecific differentiation. The greatest divergence was observed between Pakistani *H. contortus* isolates and more distantly related outgroup taxa, with genetic distance values extending from 0.37 to 1.39, underscoring pronounced evolutionary separation.

### 3.4. Median Joining Network

**Figure 5:**
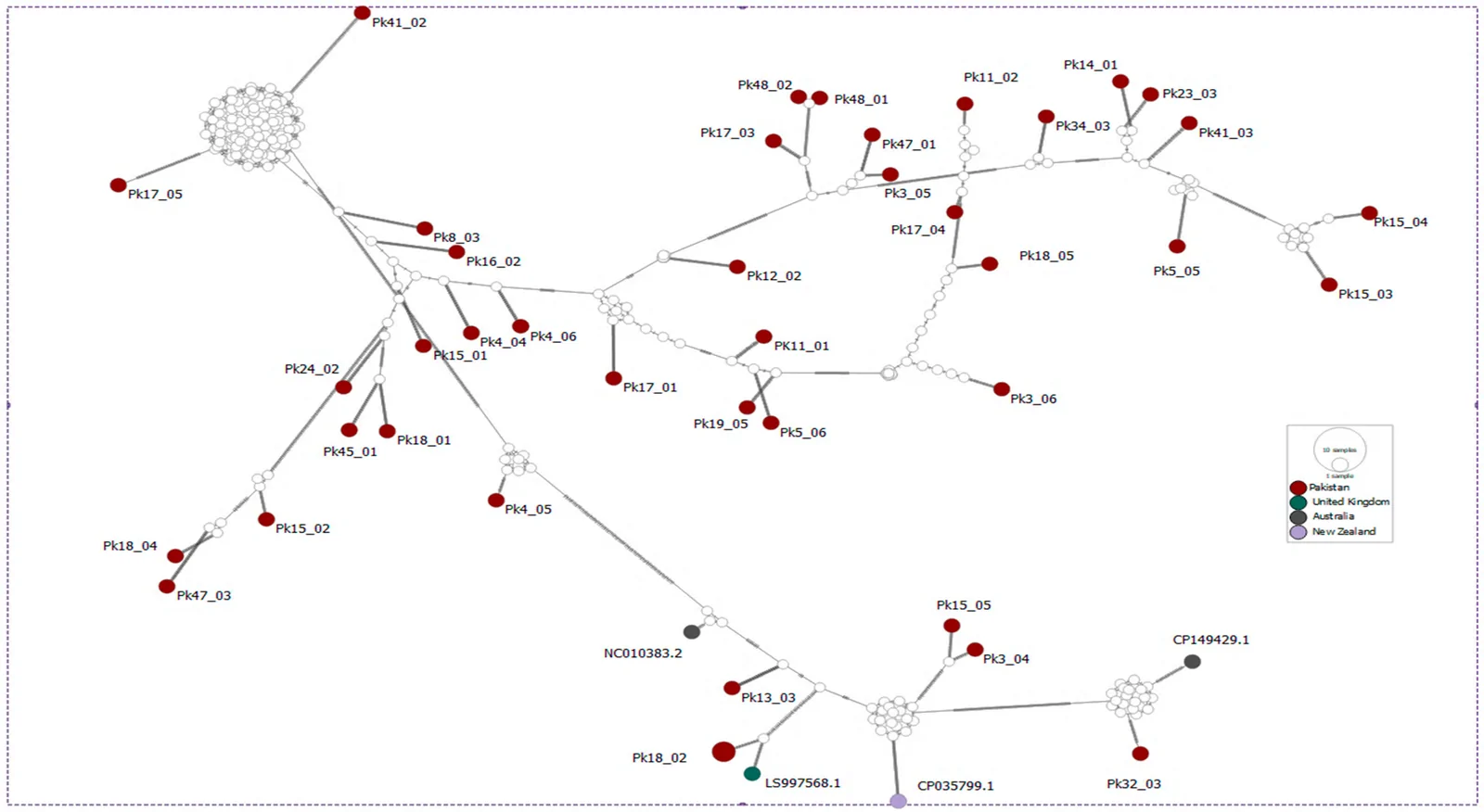
Median joining haplotype network of *H. contortus* mitochondrial genome. This network graph, created using popart v.1.7 (https://popart.maths.otago.ac.nz, accessed on 20 June 2026), represents all the mt sequences generated in this study and reference sequences from NCBI. Each circle represents a unique haplotype, and the size of the circle is proportional to the number of sequences sharing that haplotype (larger circles indicate higher frequency). Lines connecting circles represent mutational steps between haplotypes, while small intermediate nodes and the clusters of circles denote inferred unsampled or ancestral haplotypes (median vectors).

The median-joining haplotype network of *Haemonchus contortus* reveals a highly complex and reticulate genetic structure (Figure 5), indicative of substantial mitochondrial diversity within the species. Pakistani haplotypes dominate the network and are dispersed across multiple branches, rather than forming a single cohesive cluster.

Several Pakistani haplotypes occupy peripheral positions, and some are positioned close to, or directly connected with, reference sequences from the United Kingdom, Australia, and New Zealand. The abundance of median vectors throughout the network points to the existence of unsampled or extinct haplotypes.

Overall, the network topology underscores the evolutionary plasticity of *H. contortus*, emphasizing its capacity for genetic diversification and persistence across regions.

## 4. Discussion

Mitochondrial genomes have emerged as indispensable sources for deciphering the genetic variations and evolutionary links among parasitic nematodes. With the launch of next-generation sequencing technology and advancement in molecular parasitology, the reconstruction of partial and whole mitochondrial genomes has become more affordable, specifically for species identification and population genetics [13,42,43]. Understanding mitochondrial diversity within and across the parasite strains is crucial for unravelling adaptation strategies, population structure, systematics and evolutionary biology in these economically important parasites [13,44,45,46]. For these reasons, the complete mt genome of a Pakistani *H. contortus* field strain was sequenced to investigate the species-level genetic differences in these *Trichostrongyloidea*.

In the present study, the whole mitochondrial genome of *H. contortus* was evaluated, providing insights into its genome diversity and architecture. Substantial nucleotide diversity was found within the L3s population, and 923 nucleotides were identified as variable in the PCGs of the 41 samples under study. The most varied gene was Nad6 (13.6%) with 60 nucleotide variations, whereas Cox3 was the least polymorphic gene (7.41%) with 57 alterations. Based on the BLAST results, the present study’s sequences split into two clades, with one clade exhibiting 95% identity and the other 98% identity with the strain (NZ_Hco_NP) of *H. contortus* (CP035799.1) reported from New Zealand. The nucleotide diversity found in the population under study is in line with the preliminary estimates derived from mitochondrial data [47,48,49]. The high degree of genetic diversity observed in the present study is believed to be the result of great biotic potential, massive population size, brief life cycle, high infection rate coupled with elevated mutation rate present in these highly polymorphic nematodes [47,50,51].

Insights into the mt genetic heterogeneity among geographically diverse strains of *H. contortus* were obtained by evaluating the sequence variance in them. The mitogenomic comparisons between the Pakistani strain (Pk18_02) and Haecon-5 (CP149429.1) from Australia, MHco3(ISE). N1 (LS 997568) from the United Kingdom, McMaster (NC_010383.1) from Australasia, and NZ_Hco_NP (CP035799.1) from New Zealand indicated extensive nucleotide variability in the PCGs. Among the reported strains, the present study’s strain Pk18_02 showed less variability with the NZ_Hco_NP strain (CP035799.1) from New Zealand. With reference to Pk18_02, the majority of polymorphisms were detected in Nad6 (2.95), and the least variations in Nad3 (0.30) of NZ_Hco_NP (CP035799.1). Comparison among Pk18_02, McMaster (NC_010383.2) and MHco3(ISE). N1 (LS997568.1) revealed that Cox3 was the most variable, while Nad2 and Nad3 were the least variable PCGs. In comparison with Haecon-5 (CP149429.1), Nad6 (5.90%) was the most divergent and Nad4l (1.72%) was the least divergent. According to a study by [31], Cox1, Atp6, and Cox2 were the most conserved genes among the *Haemonchus* species/strains, while Nad6 and Nad2 were the most varied. The nucleotide differences across the *Haemonchus* genotypes reveal the underlying genetic heterogeneity that may be caused by adaptation to different environmental stressors or geographic isolation.

*Trichostrongyloidea* mt genomes reported so far range in length from 13,296 bp for *Dictyocaulus sp.* cf. *eckerti* [52] to 15,221 bp for *Mecistocirrus digitatus* [13,43]. The present study’s mt genome for *H. contortus* (Pk 18_02) is 14,002 bp in length, aligning closely with the size of published *H. contortus* strains, i.e., Haecon-5 with 14,107 bp [32], McMaster with 14,055 bp [29], NZ_Hco_NP with 14,001 bp [31], MHco3(ISE). N1 with 14,018 bp [41], and ChinHco with 14,092 bp in length [25], all within the expected range for *Trichostrongyloidea* mt genomes. Homogeneity indicates comparable mitochondrial genome size among the strains of *H. contortus*, suggesting limited structural variation among the populations examined to date. Larger non-coding sections between several transfer RNA genes and expansions of AT-rich regulatory regions are the main causes of the limited size disparities across the *H. contortus* strains [13].

The gene content of *H. contortus* (Pk 18_02) is in accordance with that documented for other chromadorean nematodes [13,29,32,53,54,55]. Twelve protein-coding genes (Cox1, Cox2, Cox3, Cytb, Nad1, Nad2, Nad3, Nad4, Nad4l, Nad5, Nad6, Atp6), two ribosomal RNA (lrRNA and srRNA), and twenty-two tRNA genes make up the circular *H. contortus* (Pk 18_02) mitogenome. In agreement with published mt genomes of nematodes, the Atp8 gene was absent in our data [13,29,32,41,43,56,57]. Arrangement, orientation and composition of the present study’s mitochondrial genes mirror the results observed in other *H. contortus* strains reported in NCBI GenBank from different geographical localities [13,29,32,41]. These features are similar to mt genomes of *Trichostrongyloid* nematodes, such as *C. oncophora* [58]; *T. vitrinus* and *T. axei*; *T. circumcincta*; *M. digitatus* [43,56]; *D. viviparus* and *D. eckerti* [52]; *N. spathiger* and *N. oiratianus* [57]. About 94% of the *H. contortus* (Pk 18_02) mt genome is made up of 36 genes, while non-coding regions make up the remaining ~6% [59,60,61].

Pertaining to the species of *Strongylida* reported thus far [29,54,58], the PCGs found to have the greatest AT content in *H. contortus* (Pk 18_02) were Nad2 (83.4%), Nad4l (83.4%), Nad3 (81.81%), and Nad4 (80.56%). The protein-coding genes with the lowest AT content were Cox1 (70.16%) and Nad1 (72.72%). Similar results were found by [29] while working with the field strain of *H. contortus* (McMaster) from Australia, where the PCGs with the highest AT content were Nad2–4 and Nad4l, while those with the lowest AT content were Nad1 (69.7%) and Cox1 (70.8%). ATA (Cox1, Cox2, Nad1, Nad4), ATT (Nad5, Nad4l, Atp6, Cytb), TTG (Nad2, Nad3), ATG (Cox3), and GTT (Nad6) were the initiation codons of PCGs in *H. contortus* (Pk18_02) mt genome. The predominant termination codon was TAA. Our findings closely match the study by [29], which found that the most commonly used stop codon for the *H. contortus* (McMaster) mt genes was TAA (seven genes), whereas the start codons identified for PCGs were ATT (four genes), ATA (four genes), TTG (two genes), GTT (one gene), and AAT (one gene). In an additional investigation by [13], the translation initiation codons in *H. contortus* NZ_Hco_NP were predicted to be ATT (Atp6, Cytb, Nad4, Nad4l, Nad5, Nad6), TTG (Nad2, Nad3), and ATA (Nad1 and Cox1, Cox2, Cox3) for all 12 PCGs.

*H. contortus* (Pk18_02) is no exception to the fact that all nematode mt genomes reported to date contain 22 tRNA genes. The mt genome sequence of 22 projected trn genes in *H. contortus* (Pk18_02) ranged in length from 52 to 59 bp and was related to those of other nematodes studied to date [53,54,55]. The trn genes’ sizes ranged from 53 to 61 bp in strain McMaster [29] and 54 to 63 bp in strain NZ_Hco_NP [13] of *H. contortus*. The rRNA genes (12*S* and16*S*) identified in the mt genomes of *H. contortus* (PK 18_02) were A + T biased, with 79.8% for rrnS and 82.9% for rrnL. The sizes and localities of the *rrnL* and *rrnS* genes are relatively conserved among all *Trichostrongyloidea* mt genomes analyzed [29].

Well-constructed phylogenies are essential for much population genetic research. Determining precise phylogenies for the phylum Nematoda provides crucial information about the genetic diversity and evolution of this diverse group. The phylogenetic trees of the *H. contortus* population from the present study displayed an intriguing pattern by splitting into two distant clades, signifying genetic diversity. The Pakistani isolates are distributed across multiple, well-separated haplotypes and do not form a single, geographically restricted cluster, indicating a heterogeneous parasite population with multiple maternal lineages circulating within the region. This pattern is consistent with long-term persistence of *H. contortus* in local small-ruminant populations, coupled with repeated introductions and ongoing genetic diversification. Moreover, the present study’s samples clustered with the *H. contortus* reference strain from diverse geographical localities, indicating a close genetic affinity and implying that these strains share analogous genetic traits and evolutionary history. The phylogenetic topology exhibited a high degree of concordance with earlier research [13,29,32], confirming the validity of our results. Such relationships are most plausibly explained by past livestock movements and the transboundary spread of infected animals.

## 5. Conclusions

To the best of our knowledge, this study offers the first characterized complete mitochondrial genome of *H. contortus* from Pakistani livestock. Functional insights were obtained from the thorough annotation of the mitogenome, showing a typical composition of two rRNA genes, 22 tRNA genes, and 12 PCGs. The entire mitochondrial genome of *Haemonchus* sp. described in this study supports the evolutionary stability of mitochondrial gene content and organization within Nematoda by fitting in well with the conserved genomic architecture of parasitic worms. Future comparative mitochondrial genomic research will benefit greatly from these results, which will allow for a thorough analysis of genetic variations among different strains of *H. contortus*. In conclusion, this research contributes to the global understanding of genetic diversity in *H. contortus* and plays a key role in the creation of efficient control strategies for this economically significant parasite.

## Figures and Tables

**Table 1:** Description of mitochondrial genome loci of *H. contortus* larvae.

S. No	Sample ID	Host	Length (bp)	A (%)	T (%)	G (%)	C (%)	GC (%)	AT (%)	PCG	tRNA	rRNA
1	Pk 3_04	Goat	13,997	33.35	45.34	15.00	6.29	21.30	78.70	12	22	2
2	Pk 3_05	Goat	14,016	33.38	45.07	15.05	6.29	21.34	78.45	12	22	2
3	Pk 3_06	Goat	13,997	33.2	45.04	15.17	6.57	21.74	78.26	12	22	2
4	Pk 4_04	Goat	14,007	33.34	45.04	15.02	6.59	21.61	78.38	12	22	2
5	Pk 4_05	Goat	13,999	33.43	45.20	14.96	6.40	21.36	78.63	12	22	2
6	Pk 4_06	Goat	13,991	33.29	45.13	15.08	6.49	21.57	78.42	12	22	2
7	Pk 5_05	Goat	13,990	33.20	45.08	15.13	6.58	21.71	78.28	12	22	2
8	Pk 5_06	Goat	13,990	33.33	45.18	15.02	6.47	21.49	78.51	12	22	2
9	Pk 8_03	Goat	14,000	33.33	45.11	15.04	6.52	21.56	78.44	12	22	2
10	Pk 11_01	Goat	14,000	33.22	45.15	15.12	6.51	21.63	78.37	12	22	2
11	Pk 11_02	Goat	14,005	33.23	45.10	15.11	6.56	21.67	78.33	12	22	2
12	Pk 12_02	Goat	13,997	33.27	45.06	15.13	6.53	21.66	78.33	12	22	2
13	Pk 13_03	Goat	13,999	33.10	45.21	15.23	6.47	21.70	78.31	12	22	2
14	Pk 14_01	Cattle	14,001	33.22	45.13	15.15	6.51	21.66	78.35	12	22	2
15	Pk 15_01	Cattle	13,998	33.24	45.05	15.17	6.55	21.72	78.29	12	22	2
16	Pk 15_02	Cattle	13,995	33.25	45.20	15.10	6.46	21.56	78.45	12	22	2
17	Pk 15_03	Cattle	13,992	33.11	45.11	15.26	6.49	21.75	78.22	12	22	2
18	Pk 15_04	Cattle	14,007	33.16	45.04	15.24	6.56	21.80	78.20	12	22	2
19	Pk 15_05	Cattle	13,997	33.28	45.36	15.06	6.31	21.37	78.64	12	22	2
20	Pk 16_02	Cattle	14,012	33.33	44.96	15.07	6.61	21.68	78.29	12	22	2
21	Pk 17_01	Cattle	13,990	33.24	45.02	15.17	6.54	21.71	78.26	12	22	2
22	Pk 17_03	Cattle	13,991	33.07	45.07	15.31	6.54	21.85	78.14	12	22	2
23	Pk 17_04	Cattle	13,986	33.21	45.12	15.12	6.55	21.67	78.33	12	22	2
24	Pk 17_05	Cattle	13,992	33.17	45.19	15.16	6.48	21.64	78.36	12	22	2
25	Pk 18_01	Cattle	14,011	33.19	45.03	15.17	6.61	21.78	78.22	12	22	2
26	Pk 18_02	Cattle	14,002	33.21	45.16	15.20	6.42	21.62	78.37	12	22	2
27	Pk 18_03	Cattle	14,002	33.21	45.16	15.20	6.42	21.62	78.37	12	22	2
28	Pk 18_04	Cattle	13,999	33.17	45.25	15.21	6.38	21.59	78.42	12	22	2
29	Pk 18_05	Cattle	13,990	33.22	45.06	15.18	6.54	21.72	78.28	12	22	2
30	Pk 19_05	Cattle	14,003	33.25	45.12	15.16	6.47	21.63	78.37	12	22	2
31	Pk 23_03	Goat	13,993	33.24	45.08	15.09	6.56	21.65	78.32	12	22	2
32	Pk 24_02	Goat	14,002	33.29	45.09	15.09	6.52	21.61	78.38	12	22	2
33	Pk 32_03	Goat	13,999	33.02	45.23	15.33	6.38	21.71	78.25	12	22	2
34	Pk 34_03	Goat	13,997	33.16	45.13	15.22	6.49	21.71	78.29	12	22	2
35	Pk 41_02	Goat	13,999	33.37	45.05	15.02	6.55	21.57	78.42	12	22	2
36	Pk 41_03	Goat	13,996	33.32	45.07	15.07	6.53	21.60	78.39	12	22	2
37	Pk 45_01	Goat	14,008	33.26	45.14	15.14	6.46	21.60	78.40	12	22	2
38	Pk 47_01	Goat	14,015	33.23	45.08	15.17	6.53	21.70	78.31	12	22	2
39	Pk 47_03	Goat	14,001	33.31	45.22	15.08	6.39	21.47	78.53	12	22	2
40	Pk 48_01	Goat	14,007	33.15	45.07	15.24	6.54	21.78	78.22	12	22	2
41	Pk 48_02	Goat	14,015	33.15	45.06	15.23	6.54	21.77	78.21	12	22	2

**Table 2:** Nucleotide variations in the protein-coding genes of *H. contortus* mt genome.

S. No	Gene	Length (bp)	Nucleotide Variations	Percent Variations
1	Cox1	1582	130	8.2
2	Cox2	693	59	8.5
3	Nad3	336	27	8.0
4	Nad5	1582	140	8.8
5	Nad6	441	60	13.6
6	Nad4l	232	25	10.8
7	Nad1	873	94	10.8
8	Atp6	600	55	9.2
9	Nad2	846	88	10.4
10	Cytb	1113	96	8.6
11	Cox3	769	57	7.4
12	Nad4	1230	92	7.5

**Table 3:** Comparison of the present study’s mitogenome isolate (Pk18_02) with already published genomes.

Characteristic	McMaster	NZ_Hco_NP	MHco3(ISE). N1	Haecon-5	ChinHco	Present Study
			**General Information**			
Strain	Laboratory	Field	Laboratory	Laboratory	Field	Field
Country	Australia	New Zealand	UK	Australia	China	Pakistan
Sequencing approach	454 technologies	Illumina	Multi sequencing platforms	Oxford Nanopore	Multi sequencing platforms	Illumina
Accession No	NC_010383.2	CP035799.1	LS 997568.1	CP149429.1	CNP0006302	https://data.mendeley.com/datasets/btnvv54mnp/1, accessed on 10 July 2026
			**Genome Attributes**			
Genome size (bp)	14,055	14,001	14,018	14,107	14,092	14,002
Total genes	36	36	36	36	36	36
Protein-coding genes	12	12	12	12	12	12
tRNA genes	22	22	22	22	22	22
rRNA genes	2	2	2	2	2	2
			**Base Composition**			
A (%)	33.4	32.9	33.2	33	33.41	33.21
C (%)	6.5	6.3	6.5	6.4	6.49	6.42
G (%)	15.4	14.8	15.5	15.6	15.27	15.20
T (%)	44.7	44.5	44.9	44.9	44.83	45.16
GC (%)	21.9	21.1	21.9	22	21.76	21.62
AT (%)	78	78.9	78.1	78	78.24	78.37
Predominant start codon	ATA	ATA	ATA	ATA	ATN	ATN (ATT/ATA)
Predominant stop codon	TAA	TAA	TAA	TAA	TAA	TAA
Reference	[29]	[31]	[41]	[32]	[25]	NA

ATN presents the collective designation for AT-starting initiation codons observed in the protein-coding regions.

**Table 4:** Mitochondrial genome variability of Pk (18_02) with references from NCBI.

S. No	Gene	LS997568.1		CP149429.1		CP035799.1		NC_010383.2	
		Variable Sites	Percentage of Variable Sites (%)	Variable Sites	Percentage of Variable Sites (%)	Variable Sites	Percentage of Variable Sites (%)	Variable Sites	Percentage of Variable Sites (%)
1	Cox1	35	2.21	56	3.54	27	1.71	51	3.22
2	Cox2	19	2.74	32	4.62	16	2.31	29	4.18
3	Cox3	36	4.68	39	5.07	9	1.17	45	5.85
4	Nad1	21	2.41	37	4.24	21	2.41	32	3.67
5	Nad2	10	1.18	22	2.60	17	2.01	17	2.01
6	Nad3	7	2.08	11	3.27	1	0.30	7	2.08
7	Nad4	42	3.41	51	4.15	8	0.65	45	3.66
8	Nad4l	6	2.59	4	1.72	2	0.86	6	2.59
9	Nad5	37	2.34	50	3.16	27	1.71	46	2.91
10	Nad6	11	2.49	26	5.90	13	2.95	23	5.22
11	Cytb	29	2.61	43	3.86	10	0.90	35	3.14
12	Atp6	13	2.17	28	4.67	14	2.33	31	5.17

**Table 5:** The mt genome composition of the present study’s (PK 18_02) *H. contortus* isolate.

Gene Name	Description	Start Position	Stop Position	Length (bp)	Start Codon	Stop Codon	G (%)	A (%)	T (%)	C (%)	AT (%)	GC (%)
Cox1	PCG	1	1582	1582	ATA	TAA	20.01	26.74	43.42	9.81	70.16	29.82
trnC	tRNA-Cys	1583	1636	54	_	_	9.26	46.3	40.74	3.7	87.04	12.96
trnM	tRNA-Met	1649	1705	57	_	_	21.05	35.09	31.58	12.28	66.67	33.33
trnD	tRNA-Asp	1728	1784	57	_	_	12.28	47.37	35.09	5.26	82.46	17.54
trnG	tRNA-Gly	1807	1861	55	_	_	7.27	47.27	41.82	3.64	89.09	10.91
Cox2	PCG	1862	2554	693	ATA	TAG	18.2	30.54	43.17	8.07	73.71	26.27
trnH	tRNA-His	2556	2608	53	_	_	17.64	43.13	35.3	3.92	78.43	21.56
rrnL	16S rRNA	2609	3559	951	_	_	11.27	42.34	40.95	5.42	83.3	16.7
Nad3	PCG	3557	3892	336	TTG	TAA	15.75	31.21	50.6	2.42	81.81	18.17
Nad5	PCG	3894	5475	1582	ATT	TAA	15.28	31.71	47.05	5.94	78.76	21.22
trnA	tRNA-Ala	5476	5532	57	_	_	10.53	40.35	42.11	7.02	8.43	17.55
AT rich	NCR	5533	6082	550	_	-	6.61	45.77	44.85	2.75	90.62	9.36
trnP	tRNA-Pro	6083	6138	56	_	_	17.86	41.07	35.71	5.36	76.78	23.22
trnV	tRNA-Val	6177	6231	55	_	_	7.27	49.09	41.82	1.82	90.91	9.09
Nad6	PCG	6238	6678	441	GTT	TAA	20.95	28.01	47.15	3.87	75.16	24.82
Nad4L	PCG	6685	6916	232	ATT	TAA	14.41	29.25	54.14	2.18	83.4	16.6
trnW	tRNA-Trp	6917	6973	57	_	_	7.02	43.86	43.86	5.26	87.72	12.28
trnE	tRNA-Glu	6985	7039	55	_	_	12.73	45.45	38.18	3.64	83.63	16.37
rrnS	12S rRNA	7038	7740	703	_	_	13.5	42.24	38.36	5.9	80.6	19.4
trnS1	tRNA-Ser	7741	7795	55	_	_	9.25	44.45	42.6	3.7	87.05	12.95
trnN	tRNA-Asx	7817	7872	56	_	_	14.81	37.03	42.6	5.56	79.63	20.37
trnY	tRNA-Tyr	7877	7930	54	_	_	11.32	43.39	43.39	1.9	86.78	13.22
Nad1	PCG	7931	8803	873	ATA	TAA	19.9	26.35	46.37	7.36	72.72	27.26
ATP6	PCG	8805	9404	600	ATT	TAA	17.08	29.78	46.7	6.42	76.48	23.5
trnK	tRNA-Lys	9419	9474	56	_	_	10.71	37.5	42.86	8.93	80.36	19.64
trnL1	tRNA-Leu	9483	9537	55	_	_	18.18	43.64	29.09	9.09	72.73	27.27
trnS2	tRNA-Ser	9538	9589	52	_	_	17.64	27.45	39.21	15.68	66.67	33.32
Nad2	PCG	9590	10,435	846	TTG	TAA	12.08	35.54	47.86	4.5	83.4	16.58
trnI	tRNA- Ile	10,447	10,505	59	_	_	13.33	41.67	40	5	81.67	18.33
trnR	tRNA-Arg	10,506	10,561	56	_	_	12.5	41.07	37.5	8.93	78.57	21.43
trnQ	tRNA-Gln	10,562	10,616	55	_	_	16.67	37.03	44.45	1.85	81.48	18.52
trnF	tRNA-Phe	10,658	10,711	54	_	_	9.26	50	40.74	0	90.74	9.26
Cytb	PCG	10,712	11,824	1113	ATT	TAA	16.57	29.3	46.61	7.5	75.91	24.07
trnL2	tRNA-Leu	11,825	11,880	56	_	_	11.32	47.16	35.84	5.66	83	16.98
Cox3	PCG	11,881	12,649	769	ATG	TAA	18.21	25.5	48.45	7.82	73.95	26.03
trnT	tRNA-Thr	12,647	12,701	55	_	_	9.09	43.64	43.64	3.64	87.28	12.73
Nad4	PCG	12,702	13,931	1230	ATA	TAA	11.55	31.15	49.41	7.87	80.56	19.42

The red color indicates that the stop codon of these genes is completed by the addition of A at the 3′ end of mRNA. An underscore (_) indicates that the genes are non-coding and lacks a start and/or stop codon.

## Data Availability

The data that support the findings of this study are available in the manuscript and Supplementary Materials (Tables S1–S15). Further inquiries can be directed to the corresponding author.

## Supplementary Materials

The following supporting information can be downloaded at: https://www.mdpi.com/article/10.3390/vetsci13101018/s1, Table S1: Variable positions in Cox 1 gene; Table S2: Variable sites in Cox 2 gene; Table S3: Variable sites in Cox 3 gene; Table S4: Variable sites in Nad 1 gene; Table S5: Variable sites in the Nad 2 gene; Table S6: Variable sites in Nad 3 gene; Table S7: Variable sites in Nad 4 gene; Table S8: Variable sites in Nad 4L gene; Table S9: Variable site in Nad 5 gene; Table S10: Variable sites in Nad6 gene; Table S11: Variable sites in Cytb gene; Table S12: Variable sites in Atp 6; Table S13: Percent identity (%) of the present study’s *H. contortus* mt genome isolates with the previously reported mt genome of *H. contortus*; Table S14: Comparative analysis of *H. contortus* with the reference mt genome sequences of nematodes retrieved from GenBank; Table S15: Pairwise distance analysis; Figure S1: Map of study area; Figure S2: Maximum likelihood tree of present study’s isolates. Sequence files are available at Mendeley Data: DOI:10.17632/btnvv54mnp.1.

## Abbreviations

The following abbreviations are used in this manuscript:

Mt	Mitochondrial
PCG	Protein coding genes
TE	Tris-EDTA
DTT	Dithiothreitol
qPCR	Quantitative polymerase Chain reaction
NCBI	National center for biotechnology Information
Cox	Cytochrome C oxidase
Nad	Nicotinamide adenine dinucleotide
Atp	Adenosine triphosphate
Cyt b	Cytochrome b
AT	Adenine–Thymine
GC	Guanine–Cytosine
rRNA	Ribosomal ribonucleic acid
tRNA	Transfer ribonucleic acid
BLAST	Basic local alignment search tool
HCL	Hydrochloric acid
Nacl	Sodium chloride
L3	Third stage larvae
