# Supplementary material for: Comparative Mitogenomics Reveals Mitochondrial Diversity of Haemonchus contortus, a Major Gastrointestinal Parasite of Ruminants
Source: Vet Sci. 2026 Sep 26;13(10):1018. doi: 10.3390/vetsci13101018 (PMC13709664; doi:10.3390/vetsci13101018)
Supplement: Supplementary file 1 [file vetsci-13-01018-s001.zip › vetsci-4462202-supplementary.pdf]

Supplementary Table S1: Variable positions in Cox 1 gene

	Samples																																									
S. No	Variable Site	1	2	3	4	5	6	7	8	9	10	11	12	13	14	15	16	17	18	19	20	21	22	23	24	25	26	27	28	29	30	31	32	33	34	35	36	37	38	39	40	41
1	32	G	.	.	.	.	.	A	.	.	.	.	.	.	A	.	.	.	A	.	.	.	.	.	.	.	.	.	.	.	.	A	.	.	A	.	A	.	.	.	.	.
2	33	A	G	G	G	.	G	G	G	G	G	G	G	.	G	G	G	G	G	.	G	G	G	G	G	G	.	.	G	G	G	G	G	.	G	G	G	G	G	G	G	G
3	37	T	.	.	.	.	.	.	.	.	.	.	.	.	.	.	A	.	.	.	.	.	.	.	.	.	.	.	A	.	.	.	.	.	.	.	.	.	.	A	.	.
4	51	A	.	.	.	.	.	.	.	.	.	G	.	.	G	.	.	.	.	.	.	.	.	G	.	.	.	.	.	.	.	.	.	.	.	.	.	.	.	.	.	.
5	60	T	A	A	A	.	A	A	A	A	A	A	.	.	G	A	A	A	A	.	A	A	A	A	A	A	.	.	A	A	A	.	A	.	A	A	A	A	A	A	A	A
6	78	G	A	A	A	A	.	A	A	A	A	A	A	.	A	A	A	.	A	.	A	A	A	A	A	A	.	.	A	A	A	A	A	.	A	A	A	A	A	A	A	A
7	111	A	T	T	T	T	T	T	T	T	T	T	T	.	T	T	G	T	T	.	T	T	T	T	T	T	.	.	.	T	T	T	T	.	T	T	T	T	T	.	T	T
8	117	G	.	A	.	.	.	.	A	.	A	A	A	.	.	.	.	A	.	.	.	A	.	.	A	A	.	.	.	A	A	A	.	.	.	.	A	A	.	A	G	G
9	126	A	.	G	.	G	.	.	G	.	G	G	G	.	.	.	.	.	.	.	.	.	.	G	.	.	.	.	.	G	G	.	.	.	.	.	.	.	.	.	.	.
10	129	G	.	.	.	.	.	.	.	.	.	.	.	G	.	.	.	.	.	G	.	.	.	.	.	.	.	.	.	.	.	.	.	G	.	.	.	.	G	.	.	.
11	159	A	G	G	G	G	G	G	G	G	G	G	G	.	G	G	G	G	G	.	G	G	G	G	G	G	.	.	G	G	G	.	G	.	G	G	G	G	G	G	G	G
12	165	A	T	T	T	T	T	T	T	T	T	T	T	.	T	T	T	T	T	.	T	T	T	T	T	T	.	.	T	T	T	T	T	.	T	T	T	T	T	T	T	T
13	180	T	.	.	.	.	.	.	.	.	.	.	.	.	.	.	C	.	.	.	.	.	.	.	.	.	.	.	C	.	.	.	.	.	.	.	.	.	.	C	.	.
14	207	G	A	A	A	A	A	A	A	A	A	A	A	.	A	A	A	A	A	.	A	A	A	A	A	A	.	.	A	A	A	A	A	.	A	A	A	A	A	A	A	A
15	213	C	T	.	.	T	T	.	T	.	T	.	.	.	T	T	T	T	T	.	T	.	T	.	.	T	.	.	T	T	T	T	T	T	.	T	T	.	T	T	T	T
16	222	A	.	G	.	.	.	.	.	.	.	G	.	G	.	.	.	.	.	G	.	.	.	G	.	.	G	G	.	G	.	.	.	G	.	.	.	.	.	.	.	.
17	240	A	.	.	.	.	.	.	.	.	.	.	.	.	.	.	.	.	.	.	.	.	.	.	G	.	.	.	.	.	.	.	.	.	G	.	.	.	.	G	G	G
18	246	T	.	.	.	.	.	.	.	.	.	.	.	.	.	.	.	.	A	.	.	.	.	.	.	.	.	.	.	.	.	.	.	.	.	.	.	.	.	.	A	A
19	255	T	.	C	.	.	.	.	.	.	.	.	.	.	.	.	.	.	.	.	.	.	.	.	.	.	C	C	.	.	.	.	.	.	.	.	.	.	.	.	.	.
20	258	T	A	A	A	G	A	G	A	G	A	.	G	.	G	G	.	G	G	.	G	G	A	.	A	G	.	.	.	A	A	G	A	.	G	G	G	A	A	.	A	A
21	285	A	G	.	.	.	.	.	.	.	.	.	.	.	.	.	G	.	.	.	G	.	.	.	.	.	.	.	G	.	.	.	.	.	.	G	.	.	.	.	.	.
22	294	G	A	A	A	.	.	.	A	A	A	A	A	.	.	A	.	.	.	.	A	A	.	A	A	A	.	.	.	A	A	.	A	.	.	A	.	.	A	.	.	.
23	306	A	G	.	.	.	.	.	.	.	.	.	.	.	.	.	.	.	.	.	.	G	G	.	.	.	.	.	.	.	.	.	.	.	.	.	.	.	G	.	G	G

24	321	A	.	G	.	G	G	G	G	G	.	G	G	.	G	G	G	G	.	.	G	G	.	G	G	G	.	.	G	G	G	G	G	.	G	.	G	G	G	.	G	G
25	348	T	C	.	C	C	C	.	.	C	C	C	C	.	C	.	.	.	C	.	C	C	.	C	C	.	.	.	.	C	.	C	.	.	.	.	C	.	C	.	C	C
26	357	G	.	.	.	.	.	.	.	.	.	.	.	.	.	.	A	.	.	.	.	.	.	.	.	.	.	.	A	.	.	.	.	.	.	.	.	.	.	A	.	.
27	390	G	A	A	.	A	A	A	A	A	A	A	.	.	A	A	A	A	A	.	A	A	A	A	A	.	.	.	A	A	A	A	A	.	A	A	A	.	A	A	A	A
28	393	G	.	.	.	.	.	.	.	.	.	A	.	.	.	.	.	.	.	.	.	.	.	A	.	.	.	.	A	.	.	.	.	.	.	.	.	.	.	A	A	A
29	396	G	.	A	A	A	.	A	A	A	A	A	A	.	A	A	A	A	A	.	A	A	.	.	A	A	.	.	A	A	A	A	A	.	A	A	A	A	.	.	.	.
30	420	C	G	G	A	G	A	G	G	A	G	G	G	.	A	G	G	G	G	.	A	G	G	A	G	A	.	.	G	A	G	G	G	.	A	G	A	A	G	G	G	G
31	423	A	.	.	.	.	.	.	.	.	.	.	.	.	G	.	.	.	.	.	.	.	.	.	.	.	G	G	.	.	.	.	.	G	.	.	.	.	.	.	.	.
32	429	A	.	.	.	.	.	.	.	.	G	.	.	.	.	G	.	.	.	G	.	.	.	.	.	.	.	.	.	.	.	.	.	.	.	.	.	G	.	.	.	.
33	435	A	.	.	.	.	.	.	.	.	.	.	.	.	G	G	G	.	.	G	.	G	.	.	.	.	.	.	.	.	.	.	.	.	.	.	.	.	.	.	.	.
34	438	T	A	A	A	A	A	A	A	A	A	A	A	.	A	A	A	A	A	.	A	A	A	A	A	A	.	.	A	A	A	A	A	.	A	A	A	A	A	A	A	A
35	441	T	.	.	.	.	.	.	.	.	.	.	.	C	.	.	.	.	.	.	.	C	.	.	.	C	.	.	.	.	.	.	.	.	.	.	C	.	.	.	.	.
36	444	T	G	G	G	G	.	C	G	G	G	G	G	C	.	G	G	G	G	.	G	G	G	G	G	G	C	C	G	G	G	.	G	C	.	G	.	G	G	G	G	G
37	447	A	.	.	.	.	.	.	.	.	.	.	.	G	.	.	G	.	.	.	.	G	.	.	G	G	G	G	G	.	.	.	.	G	.	.	.	G	.	G	.	.
38	450	T	.	.	.	.	.	.	.	.	.	.	.	.	.	.	A	A	.	.	.	.	.	.	.	.	.	.	A	.	.	.	.	.	.	.	.	.	.	.	.	.
39	453	T	A	G	A	A	A	G	A	A	A	A	A	.	A	A	A	A	A	.	.	A	A	A	A	A	.	.	.	A	A	A	A	.	A	.	A	A	A	A	A	A
40	456	G	A	A	A	.	A	.	A	.	A	.	.	.	.	A	A	.	.	.	A	A	A	.	A	.	.	.	.	A	.	.	A	.	.	.	.	A	A	.	A	A
41	474	T	A	G	A	A	A	A	A	A	A	A	A	.	A	A	A	A	A	.	A	A	A	A	A	A	.	.	A	A	A	A	A	.	A	A	A	A	A	A	A	A
42	477	T	.	.	.	.	.	.	.	.	.	.	C	.	.	.	.	.	C	.	.	.	.	.	.	.	.	.	.	.	A	.	.	.	.	.	.	.	.	.	A	A
43	486	G	.	.	.	.	.	.	.	.	.	.	.	.	A	.	A	.	.	.	.	A	.	.	.	.	.	.	A	.	.	.	.	.	.	.	A	.	.	A	.	.
44	495	A	.	.	.	.	.	.	G	.	.	.	.	.	.	.	.	.	.	G	.	.	.	.	.	.	.	.	.	.	.	.	.	.	.	.	.	.	G	.	.	.
45	498	A	.	.	.	.	.	.	.	C	.	.	.	.	.	.	.	.	.	.	.	.	.	.	C	G	.	.	.	.	G	.	.	.	.	G	.	.	.	.	.	.
46	507	G	.	.	.	.	.	.	.	.	.	.	.	.	.	.	.	A	.	A	.	.	.	A	.	.	.	.	.	.	.	A	.	.	.	.	.	.	.	.	.	.
47	522	G	A	A	A	A	A	A	A	A	A	A	A	.	A	A	A	A	A	.	A	A	A	A	A	A	.	.	A	A	A	A	A	.	A	A	A	A	A	A	A	A
48	525	C	T	T	T	T	T	T	T	T	T	T	T	.	T	T	T	T	T	.	T	T	T	T	T	T	T	T	T	T	T	T	T	.	T	T	T	T	T	T	T	T
49	528	A	.	.	.	.	.	.	.	.	.	.	.	.	.	.	T	.	.	.	.	.	.	.	.	.	.	.	T	.	.	.	.	.	.	.	.	.	.	T	.	.
50	549	G	A	A	A	A	C	A	A	A	A	A	A	.	A	.	A	A	A	.	A	A	A	A	A	A	.	.	A	A	A	A	A	.	A	A	A	A	A	A	A	A
51	555	T	C	.	C	.	C	.	C	.	C	C	C	.	.	C	.	.	.	.	C	.	C	C	.	.	.	.	.	.	C	.	.	.	.	C	.	.	.	.	C	C
52	561	A	G	G	G	G	G	G	G	G	G	G	G	.	G	G	G	G	G	.	G	G	G	G	G	G	.	.	G	G	G	G	G	.	G	G	G	G	G	G	G	G
53	570	A	.	G	G	.	G	G	.	.	G	G	.	.	.	G	.	.	.	.	.	G	G	G	G	.	.	.	.	G	G	.	G	.	.	G	.	G	G	.	G	G

54	639	T	.	.	.	.	.	C	.	C	C	.	.	C	.	C	C	C	.	.	.	.	C	C	C	.	.	C	.	.	.	.	.	C	.	C	.	.	C	.	.
55	651	A	G	G	G	G	G	G	G	G	G	G	G	.	G	G	G	G	G	.	G	G	G	G	G	G	.	.	G	G	G	G	G	G	G	G	G	G	G	G	G	G
56	681	A	G	G	G	G	G	G	G	G	G	G	G	.	G	G	G	G	G	.	G	G	G	G	G	G	.	.	G	G	G	G	G	.	T	G	G	G	G	G	G	G
57	690	C	T	T	T	T	T	T	T	T	T	T	T	T	T	T	T	T	T	.	T	T	T	T	T	T	.	.	T	T	T	T	T	.	T	T	T	T	.	T	T	T
58	696	G	A	A	A	A	A	A	A	A	A	A	A	A	A	A	.	A	A	.	A	A	A	A	A	A	.	.	.	A	A	A	A	.	A	A	A	A	A	A	A	A
59	711	G	.	.	.	.	.	A	.	.	.	.	.	.	.	.	A	.	.	.	.	.	.	.	.	.	.	.	.	.	.	.	.	.	A	.	.	.	A	A	A	A
60	723	A	.	.	.	G	.	.	.	.	.	.	.	.	.	.	G	.	.	.	.	.	.	.	.	.	.	.	G	.	.	.	.	.	.	.	.	.	.	G	.	.
61	726	T	C	C	C	.	C	C	C	C	C	C	C	.	C	C	C	C	C	.	C	C	C	C	C	C	.	.	C	C	C	C	C	.	C	C	C	C	C	C	C	C
62	741	T	.	.	.	.	.	.	.	.	.	.	.	.	.	C	.	.	.	.	.	.	.	.	.	C	.	.	.	.	C	.	.	.	.	.	.	.	.	C	.	.
63	747	T	.	.	.	.	.	.	.	.	.	.	.	.	.	.	.	.	.	.	.	.	.	.	.	.	.	.	.	.	.	.	C	C	.	.	.	.	.	.	.	.
64	750	T	C	C	C	C	C	.	C	C	.	C	C	C	.	C	.	.	.	.	C	.	C	C	C	C	.	.	.	.	.	C	C	.	.	.	.	.	C	.	.	.
65	771	A	G	G	G	.	G	G	G	G	G	G	G	.	G	G	G	G	G	.	G	G	G	G	G	G	.	.	G	G	G	G	G	.	G	G	G	G	G	G	G	G
66	783	G	A	A	A	A	A	A	A	A	A	A	A	.	A	A	.	A	A	.	A	.	A	A	A	A	.	.	A	A	A	A	A	.	A	A	A	A	A	A	A	A
67	798	A	G	G	G	.	G	G	G	G	G	G	G	.	.	G	G	G	G	.	G	G	G	G	G	G	.	.	G	G	G	G	G	.	G	.	G	G	.	G	G	G
68	804	A	G	G	G	.	G	G	G	G	G	G	G	.	G	G	G	G	G	.	G	G	G	G	G	G	.	.	G	G	G	G	G	.	G	G	G	G	G	G	G	G
69	813	A	G	.	.	.	.	G	.	.	.	.	.	.	G	.	G	G	G	.	.	.	.	.	.	.	.	.	G	.	.	G	.	.	G	.	G	.	G	.	G	G
70	816	A	G	G	.	.	G	.	G	G	G	G	G	.	.	G	G	.	.	.	G	G	G	G	.	G	.	.	G	G	G	.	G	.	.	G	.	G	G	G	.	.
71	822	G	A	A	A	.	A	A	A	A	A	A	A	.	A	A	A	A	A	.	A	A	.	A	A	A	.	.	A	A	A	A	A	.	A	A	A	A	A	A	A	A
72	825	A	G	G	G	.	G	.	.	G	G	G	.	.	G	G	G	G	G	.	.	G	G	G	.	G	.	.	G	G	G	G	G	.	G	.	G	G	G	G	G	G
73	846	A	G	G	G	G	.	.	G	G	G	G	G	G	G	.	.	.	.	G	.	G	G	G	G	.	.	.	.	G	G	.	G	.	G	.	.	.	.	G	G	G
74	852	T	C	.	.	.	.	.	.	.	.	.	.	C	.	.	C	.	.	.	.	.	.	C	.	.	C	C	C	.	.	.	.	.	.	.	.	.	C	.	C	C
75	870	A	G	G	G	.	G	G	G	G	G	G	G	.	G	G	G	G	G	.	G	G	G	G	G	G	.	.	G	G	.	G	G	.	G	G	G	.	G	G	G	G
76	873	A	G	.	G	.	G	.	.	G	.	.	G	.	.	G	G	.	.	.	G	G	G	.	G	G	.	.	G	.	.	.	.	.	.	G	.	G	.	.	G	G
77	885	G	A	A	A	.	A	A	A	A	A	A	A	.	A	.	A	A	A	.	.	A	A	A	A	A	A	A	A	A	A	A	.	.	A	A	A	A	A	A	A	A
78	888	G	A	.	.	.	.	.	.	.	.	.	.	.	.	.	.	.	.	.	.	.	.	.	.	.	.	.	.	.	.	.	.	.	.	.	.	.	.	.	A	A
79	891	G	.	.	.	.	.	.	.	.	.	.	.	.	.	A	.	.	.	.	.	.	.	.	.	.	.	.	.	.	.	.	A	.	.	.	.	.	A	A	.	.
80	897	T	.	.	C	.	.	.	.	.	.	.	.	C	.	C	.	.	C	.	.	.	.	.	.	.	C	C	.	.	.	C	.	C	C	.	.	.	.	.	.	.
81	900	T	.	C	.	.	.	.	.	.	.	.	.	.	.	.	.	.	.	.	.	.	.	.	.	.	C	C	.	.	.	.	.	.	.	.	.	.	.	C	.	.
82	909	A	.	G	G	.	G	.	.	G	G	G	.	.	G	G	G	G	G	.	.	G	.	G	G	G	.	.	G	G	G	G	G	.	G	.	G	G	.	G	.	.
83	912	G	.	.	A	A	.	T	A	.	.	.	A	A	T	.	.	T	T	.	A	.	.	.	T	.	A	A	.	.	.	T	T	A	T	A	T	.	.	.	A	A

84	915	T	.	.	.	.	.	.	.	.	.	.	.	.	.	.	.	.	.	.	.	.	.	.	.	.	C	C	.	.	.	.	.	.	.	.	C	.	.	.	.	.
85	930	G	.	.	.	.	.	.	.	.	.	.	.	.	A	.	.	.	.	.	.	.	.	.	.	.	.	.	A	.	.	.	.	.	.	.	.	.	.	A	.	.
86	951	T	.	.	C	.	.	.	.	C	.	.	.	C	.	.	.	.	.	.	.	.	.	.	A	.	.	.	.	.	.	.	.	.	.	.	.	.	.	.	.	.
87	960	A	.	.	.	.	.	.	.	.	.	.	.	.	.	.	.	.	.	G	G	.	.	.	.	.	.	.	.	.	.	.	.	.	.	.	.	G	.	.	.	.
88	969	T	.	.	.	.	C	.	.	.	.	.	.	.	.	.	.	.	.	.	.	C	.	.	.	.	.	.	.	.	.	C	.	.	.	.	.	.	.	.	.	.
89	972	T	.	.	C	.	C	C	.	.	.	.	.	.	.	C	C	.	.	.	.	.	C	.	.	.	.	.	.	.	.	.	C	.	.	.	.	.	.	C	C	C
90	975	A	T	T	T	T	T	T	T	C	C	T	T	T	T	.	C	T	T	.	T	T	C	T	T	T	.	.	C	T	T	T	.	.	T	T	T	T	T	T	T	T
91	978	G	.	.	.	.	A	.	A	.	.	.	.	.	.	.	.	A	.	.	.	.	.	.	.	.	A	A	.	.	.	.	.	.	.	.	.	.	.	A	.	.
92	996	A	G	G	G	G	G	G	G	G	.	G	G	G	G	.	G	G	G	.	G	G	G	G	G	G	G	G	G	G	G	G	G	.	G	G	G	G	G	G	G	G
93	1005	T	C	.	.	.	.	.	.	.	.	C	.	.	.	.	.	.	.	.	.	.	.	.	.	.	.	.	.	.	.	.	.	.	C	.	.	.	.	.	.	.
94	1014	A	G	G	G	G	.	G	G	G	G	G	G	G	G	G	G	G	G	.	.	G	.	G	G	G	.	.	G	G	G	G	G	T	G	.	G	G	G	G	G	G
95	1023	A	.	.	.	.	.	.	.	.	.	.	.	G	.	G	.	.	.	.	.	.	.	.	.	.	.	.	.	.	.	.	G	.	.	.	.	.	.	.	.	.
96	1038	G	.	.	.	.	.	.	.	.	.	.	.	.	.	.	.	A	A	.	.	.	.	.	.	.	.	.	.	.	.	.	.	.	.	.	.	.	A	.	.	.
97	1053	G	A	.	.	.	.	.	.	.	.	.	.	.	.	.	A	.	.	.	.	.	.	.	.	.	.	.	A	.	.	A	.	.	.	A	.	.	.	.	.	.
98	1074	A	.	.	.	.	.	.	G	.	.	.	.	.	.	.	.	.	.	.	.	.	.	.	.	.	G	G	.	.	.	.	.	.	.	.	.	.	.	.	.	.
99	1080	A	.	.	G	.	.	.	.	G	.	.	.	.	.	.	.	.	.	.	.	G	.	.	G	.	.	.	G	.	.	.	.	.	G	T	.	.	.	.	.	.
100	1083	A	G	G	G	G	G	G	G	G	G	G	G	.	G	G	.	G	G	.	.	G	G	G	G	G	.	.	.	G	G	G	G	G	G	.	G	G	G	.	G	G
101	1086	A	.	.	.	.	G	.	.	.	.	.	.	.	.	.	G	.	.	.	.	.	.	.	G	.	.	.	G	.	.	.	.	.	.	.	.	.	.	.	.	.
102	1095	G	A	A	A	A	A	A	A	A	A	A	A	A	A	A	A	A	A	.	A	A	A	A	A	A	T	T	A	A	A	A	A	A	A	A	A	.	A	A	A	A
103	1110	A	.	C	.	.	.	.	C	.	C	C	.	.	.	.	.	.	.	.	.	.	G	C	.	.	.	.	.	C	C	.	.	.	.	.	.	.	.	.	.	.
104	1131	T	C	C	C	C	C	C	C	C	.	C	C	C	C	C	C	C	C	.	.	C	C	C	C	C	C	C	C	C	.	C	C	A	C	.	C	C	C	C	C	C
105	1140	T	.	.	.	.	.	.	.	.	.	.	.	.	.	.	C	.	.	.	.	.	.	.	.	.	.	.	C	.	.	.	C	.	.	.	.	.	.	.	.	.
106	1149	T	A	A	A	A	A	A	A	A	A	A	A	.	A	A	A	A	A	.	A	A	A	A	A	A	.	.	A	A	A	A	A	.	A	A	A	A	A	A	A	A
107	1176	G	A	.	.	.	.	A	.	.	.	.	.	.	.	.	A	A	A	.	.	.	A	.	.	.	.	.	A	.	.	A	.	.	A	.	A	.	A	A	A	A
108	1191	G	.	.	.	.	.	.	.	.	A	.	.	A	.	.	A	.	.	.	.	A	.	.	.	.	.	.	A	.	.	.	A	.	.	.	.	.	.	.	.	.
109	1218	G	A	.	A	A	A	A	A	A	A	A	.	.	A	A	A	A	A	.	A	A	A	A	A	A	.	.	A	A	A	A	A	A	A	A	A	A	A	A	A	A
110	1227	A	.	G	.	.	.	.	.	.	.	G	.	.	.	.	.	.	.	.	G	.	.	G	.	.	.	.	.	.	.	.	.	.	.	.	.	.	.	.	.	.
111	1230	G	T	T	T	C	T	T	T	C	T	T	C	.	T	C	T	T	T	.	C	T	T	T	C	C	.	.	T	T	T	T	C	.	T	C	T	C	T	T	T	T
112	1242	T	C	C	C	.	C	C	C	C	C	C	C	.	C	.	.	C	C	.	.	C	C	C	C	C	.	.	.	C	C	C	.	.	C	.	C	C	C	.	C	C
113	1257	A	.	.	.	.	.	.	.	.	G	.	.	.	.	.	.	.	.	G	G	.	.	.	.	.	.	.	.	.	.	.	.	G	.	G	.	.	.	.	.	.

114	1263	A	G	G	G	.	G	G	G	G	G	G	G	.	G	G	G	G	G	.	G	G	G	G	G	G	.	.	G	G	G	G	G	.	G	G	G	G	.	G	G	G
115	1266	A	G	G	G	G	G	G	G	G	G	G	G	.	G	G	G	G	G	.	G	G	G	G	G	G	.	.	G	G	G	G	G	.	G	G	G	G	G	G	G	G
116	1290	A	.	G	G	.	.	.	G	G	G	G	.	.	G	G	G	.	.	.	G	G	G	G	.	G	.	.	.	G	G	G	G	.	G	.	G	.	G	.	.	.
117	1305	T	.	.	.	.	.	.	.	.	.	C	.	.	C	.	.	C	.	.	.	.	.	.	.	.	.	.	.	.	.	C	.	.	C	.	C	.	.	.	.	.
118	1329	T	.	.	.	.	.	.	.	C	C	.	.	.	.	.	.	.	.	C	.	.	.	.	C	.	.	.	C	.	.	.	.	.	.	.	.	.	.	.	.	.
119	1338	G	A	A	A	A	.	A	A	.	A	A	A	.	A	A	A	.	A	.	A	A	A	A	.	A	.	.	A	A	A	A	A	.	A	A	A	.	A	A	A	A
120	1344	A	A	G	A	A	A	A	A	A	A	A	A	A	A	A	A	A	A	A	A	A	A	A	A	A	A	A	A	A	A	A	A	A	A	A	A	A	A	A	A	A
121	1350	A	G	.	.	.	.	G	.	.	.	.	.	.	.	.	.	.	.	.	.	.	G	.	.	.	.	.	.	G	.	.	.	.	G	.	.	.	.	.	G	G
122	1359	C	A	A	A	T	A	A	A	A	A	T	A	.	A	A	A	A	A	.	A	A	A	T	A	A	.	.	A	A	A	A	A	.	A	A	A	A	A	A	A	A
123	1383	A	G	.	.	.	G	.	.	.	.	.	.	.	G	.	.	G	G	.	.	G	G	.	G	.	.	.	.	.	.	G	.	.	G	.	G	.	G	.	G	G
124	1395	T	.	C	C	.	.	.	C	.	C	C	.	.	.	.	C	.	.	C	.	C	.	C	.	C	.	.	C	C	C	C	.	.	.	.	.	.	.	C	.	.
125	1407	A	.	.	.	T	.	.	.	.	.	.	.	T	.	.	.	G	G	T	.	.	.	G	G	.	T	T	.	.	.	.	.	.	.	G	G	.	.	.	G	G
126	1449	G	.	.	.	.	.	.	A	A	.	.	.	.	.	.	.	.	.	.	.	A	A	.	.	.	.	.	.	.	.	.	.	.	.	.	.	.	.	.	.	.
127	1471	G	A	A	A	.	A	A	A	A	A	A	A	.	A	A	A	A	.	A	A	A	A	A	A	A	.	.	A	A	A	A	A	.	A	A	A	A	A	A	A	A
128	1536	T	.	C	.	.	.	.	C	.	C	.	.	.	.	.	.	.	.	.	.	.	.	.	.	.	.	.	.	C	C	.	.	.	.	.	.	.	.	.	.	.
129	1566	C	T	T	T	.	T	T	T	T	T	T	T	.	T	T	T	T	T	.	T	T	T	T	T	T	.	.	T	T	T	T	T	.	T	T	T	T	T	T	T	T
130	1569	G	A	A	A	.	A	A	A	A	A	A	A	.	A	A	A	A	A	.	A	A	A	A	A	A	.	.	A	A	A	A	A	.	A	A	A	A	A	A	A	A

Note: The column numbers (1–41) correspond to the S. No. assigned to the respective isolates in Table 1.

Supplementary Table S2: Variable sites in Cox 2 gene

S. No	Samples																																									
	Vari	1	2	3	4	5	6	7	8	9	10	11	12	13	14	15	16	17	18	19	20	21	22	23	24	25	26	27	28	29	30	31	32	33	34	35	36	37	38	39	40	41
1	24	A	G	.	.	.	G	.	G	.	.	.	.	.	.	.	.	G	G	.	G	.	G	G	.	G	.	.	.	G	.	.	.	.	.	G	.	G	G	G	G	G
2	42	C	T	T	T	.	T	T	T	T	T	T	T	.	T	T	T	T	T	.	T	T	T	T	T	T	.	.	T	T	T	T	T	.	T	T	T	T	T	T	T	T
3	63	A	.	.	.	.	.	.	.	.	.	.	G	.	.	.	G	.	.	.	.	.	.	.	.	.	G	G	.	.	.	.	.	G	.	.	.	.	.	.	.	.
4	93	A	.	.	.	.	.	G	.	.	.	.	.	.	.	.	.	.	.	.	.	.	.	.	.	.	.	.	.	.	.	.	.	G	.	G	.	.	.	.	.	.
5	99	A	G	.	G	.	G	G	.	G	.	.	.	G	G	G	G	G	G	.	G	G	G	.	G	G	.	.	G	.	.	.	G	.	G	G	G	G	G	G	G	G
6	102	G	.	.	.	A	.	A	.	.	.	.	.	.	.	.	.	.	.	.	.	A	.	.	.	.	A	A	.	.	.	.	.	.	.	.	.	.	.	.	.	.
7	106	C	T	T	T	.	T	T	T	T	T	T	T	.	T	T	T	T	T	.	T	T	T	T	T	T	T	T	T	T	T	T	T	T	T	T	T	T	T	T	T	T
8	108	A	.	.	.	.	.	.	.	.	.	.	.	.	.	.	.	.	.	.	.	.	.	.	.	.	G	G	.	.	G	.	.	.	.	.	.	.	.	.	.	.
9	132	A	T	T	T	.	T	T	T	T	T	T	T	.	T	T	T	T	T	.	T	T	T	T	T	T	.	.	T	T	T	T	T	.	T	T	T	T	T	T	T	T
10	138	A	.	G	G	.	.	.	G	.	.	G	G	.	.	G	.	.	.	.	G	G	.	G	G	.	.	.	.	G	G	.	.	.	.	G	.	G	.	.	.	.
11	144	A	.	.	.	.	.	.	G	.	.	.	.	.	.	.	.	.	.	.	.	.	.	.	.	.	G	G	.	.	.	.	.	G	.	.	.	.	.	.	.	.
12	156	C	T	T	T	.	T	T	T	T	T	T	T	.	T	T	T	T	T	.	T	T	T	T	T	T	.	.	T	T	T	T	T	.	T	T	T	T	T	T	T	T
13	162	G	A	A	A	.	A	A	A	A	A	A	A	.	A	A	A	A	A	.	A	A	A	A	A	A	.	.	A	A	A	A	A	.	A	A	A	A	A	A	A	A
14	168	G	A	A	A	.	A	A	A	A	A	A	A	A	A	A	A	A	A	.	A	A	A	A	A	A	A	A	A	A	A	A	A	A	A	A	A	A	A	A	A	A
15	192	G	A	.	A	.	A	A	A	A	A	A	A	.	A	A	A	A	A	.	.	A	A	A	A	A	.	.	A	A	A	A	A	A	A	.	.	A	A	A	A	A
16	213	T	A	A	A	.	A	A	A	A	G	A	A	.	A	A	A	A	A	.	A	A	A	A	A	A	.	.	A	A	A	A	A	.	A	A	A	A	A	A	A	A
17	225	A	G	G	G	.	G	G	G	G	G	G	G	.	G	G	G	G	G	.	G	G	G	G	G	G	.	.	G	G	G	G	G	.	G	G	G	G	G	G	G	G
18	228	G	A	A	A	.	A	A	A	A	A	A	A	.	A	A	A	A	A	.	A	A	A	A	A	A	.	.	.	A	A	A	A	.	A	A	A	A	.	A	A	A
19	255	A	.	.	.	.	.	.	.	.	.	.	.	.	.	G	G	.	.	.	.	.	.	.	.	.	.	.	G	.	.	.	.	.	.	.	.	.	.	G	.	.
20	258	A	.	G	G	.	.	G	.	.	G	G	.	.	G	.	G	G	G	.	.	G	.	G	.	.	G	G	G	G	G	G	.	.	G	.	G	.	.	G	.	.
21	267	T	.	.	.	.	.	C	.	.	.	.	.	.	C	.	.	C	C	.	.	.	.	.	.	.	.	.	C	.	.	C	.	.	C	.	C	.	.	.	.	.
22	270	G	.	.	.	.	.	.	.	.	.	A	.	.	.	.	.	.	.	A	.	.	.	A	.	.	A	A	.	.	.	.	.	A	.	.	.	.	.	.	.	.
23	273	A	.	.	.	G	.	.	.	.	.	.	.	.	.	.	.	.	.	.	.	.	.	.	.	.	G	G	.	.	.	.	.	.	.	.	.	G	.	G	.	.

24	288	T	.	C	C	.	.	C	.	C	C	C	C	.	C	C	C	.	C	.	C	C	C	C	C	C	.	.	C	C	.	C	.	.	C	C	C	C	C	C	C	C
25	294	G	A	A	A	.	A	A	A	A	A	A	A	.	A	A	A	A	A	.	A	A	A	A	A	A	A	A	A	A	A	A	A	.	A	A	A	A	A	A	A	A
26	300	T	C	C	C	.	C	C	C	C	C	C	C	.	C	C	C	C	C	.	.	C	C	C	C	C	.	.	.	C	C	C	C	.	C	.	C	C	C	C	C	C
27	306	C	A	A	A	.	A	A	A	A	A	A	A	T	A	A	A	A	A	.	A	A	A	A	A	A	T	T	A	A	A	A	A	T	A	A	A	A	A	A	A	A
28	312	A	.	G	T	.	G	G	G	G	G	G	G	.	G	G	.	G	G	.	G	G	G	G	G	G	.	.	.	G	.	G	G	G	G	G	G	G	G	.	G	G
29	318	A	.	.	.	.	.	.	.	.	.	.	.	.	.	.	G	.	.	.	.	.	.	.	G	.	.	.	.	.	.	.	.	.	.	.	.	.	.	G	.	.
30	321	A	.	G	G	.	G	.	G	G	G	G	.	.	.	G	.	.	.	.	G	G	.	G	G	G	.	.	.	G	G	.	G	.	.	G	.	G	.	.	.	.
31	339	T	C	C	C	.	C	C	C	C	C	C	C	.	C	C	.	C	.	.	C	.	C	C	C	.	.	.	.	C	.	C	C	.	C	C	C	C	C	.	.	.
32	345	T	.	.	.	.	.	.	.	.	.	.	.	.	.	.	.	.	.	.	.	.	.	.	.	.	C	C	.	.	.	.	.	.	.	.	.	.	.	C	.	.
33	348	T	.	C	.	.	.	.	.	.	.	C	.	.	.	.	.	.	C	.	.	.	.	C	.	.	.	.	.	C	.	.	.	.	.	.	.	.	.	C	.	.
34	351	T	A	.	.	.	.	.	.	.	.	.	.	.	.	.	.	.	C	.	.	.	A	.	.	.	.	.	.	.	.	.	.	.	.	.	.	.	A	.	A	A
35	384	G	.	.	.	.	.	.	.	.	.	.	.	.	.	A	.	.	.	.	.	.	.	.	.	.	.	.	.	.	.	A	.	A	.	.	.	.	.	.	.	.
36	399	G	A	A	A	A	A	A	A	A	A	A	A	.	A	A	A	A	A	.	A	A	A	A	A	A	.	.	A	A	A	A	A	.	.	.	.	A	A	A	A	A
37	417	A	.	.	.	.	.	.	.	.	.	.	.	.	.	.	.	.	.	.	.	.	.	.	.	.	G	G	.	.	.	.	.	.	.	.	.	.	.	G	.	.
38	448	G	.	.	.	.	.	A	.	A	.	.	.	.	.	A	.	.	.	.	.	.	.	A	.	A	.	.	.	.	.	.	.	.	.	.	.	.	.	.	.	.
39	477	T	.	.	.	.	.	.	.	.	.	A	A	C	.	.	.	.	.	.	.	.	.	A	.	.	.	.	.	.	.	.	.	.	.	.	.	.	.	A	.	.
40	480	T	C	C	C	.	C	.	C	.	C	.	C	A	.	C	C	.	.	.	C	C	C	.	.	C	A	A	C	C	.	.	.	A	.	.	C	.	C	.	C	C
41	507	G	.	.	.	.	.	.	.	.	.	.	A	.	.	.	.	.	.	.	.	.	.	.	A	.	.	.	.	.	A	.	.	.	.	A	.	.	T	.	.	.
42	510	G	A	A	A	A	A	A	A	A	A	A	A	.	A	A	A	A	A	.	A	A	A	A	A	A	.	.	A	A	A	A	A	.	A	A	A	A	A	A	A	A
43	525	G	A	A	A	A	A	A	A	A	A	A	.	.	A	A	A	A	A	.	A	A	A	A	A	A	.	.	A	A	A	A	A	.	A	.	A	A	A	A	A	A
44	540	T	G	A	G	.	A	A	A	A	A	A	A	.	A	A	A	G	A	.	A	A	G	G	A	G	.	.	A	A	A	A	A	.	G	A	.	A	G	G	G	G
45	564	A	.	.	.	.	.	.	.	.	.	.	.	.	.	.	.	.	.	.	.	.	.	.	.	.	.	.	.	.	.	.	G	.	.	.	.	.	.	.	.	.
46	571	C	T	T	T	.	T	T	T	T	T	T	T	T	T	T	T	T	T	.	T	T	T	T	T	T	.	.	T	T	T	T	T	.	T	T	T	T	T	T	T	T
47	573	G	A	A	A	.	A	.	A	A	A	A	A	.	.	A	A	.	.	.	A	A	A	A	A	A	.	.	A	A	A	.	A	.	.	A	.	A	A	A	A	A
48	574	A	G	G	G	.	G	G	G	G	G	G	G	.	G	G	.	G	G	.	G	G	G	G	G	G	.	.	.	G	G	G	G	.	G	G	G	G	G	.	G	G
49	576	T	.	.	.	C	A	.	.	C	.	.	.	.	.	.	.	.	.	.	.	.	.	C	.	.	.	.	.	.	.	.	C	.	.	.	.	.	.	.	.	.
50	582	G	.	.	.	.	.	.	.	.	.	.	.	.	.	.	.	.	.	A	.	.	.	.	.	.	.	.	.	.	.	.	.	.	.	.	.	.	.	A	.	.
51	600	G	A	A	A	.	A	A	T	A	A	A	A	.	A	A	A	A	A	.	A	A	A	A	A	A	.	.	A	A	A	A	A	.	A	.	A	A	A	A	A	A
52	618	T	C	.	.	.	.	.	.	.	.	.	.	.	.	.	.	.	.	.	.	.	C	.	C	.	.	.	.	.	C	.	.	.	C	.	.	.	.	.	C	C
53	621	T	.	.	.	.	.	.	.	.	.	.	.	.	.	.	.	C	C	.	C	.	.	.	C	.	.	.	.	.	.	C	.	.	.	.	.	.	.	.	.	.

54	627	T	.	.	.	.	.	.	.	.	.	.	.	.	.	.	.	.	.	.	.	.	.	.	.	C	C	.	.	.	.	.	.	.	.	.	C	.	.	.	.
55	633	T	.	.	.	.	.	.	.	.	.	.	.	.	C	.	.	.	.	.	.	.	.	.	.	.	.	C	.	.	.	.	.	.	C	.	C	.	.	C	C
56	645	G	.	.	.	.	.	.	.	.	.	.	.	.	.	.	.	.	.	.	A	.	.	.	.	.	.	A	.	.	.	.	.	.	.	.	.	A	.	.	.
57	648	A	.	G	.	.	.	.	G	G	.	.	.	.	.	.	.	.	.	G	.	.	.	.	G	G	G	G	.	G	.	.	.	G	.	.	.	.	.	.	.	.
58	675	G	A	A	.	.	.	A	A	A	.	A	A	.	A	A	.	A	A	.	A	A	A	A	A	.	.	.	A	A	A	A	A	.	A	A	A	A	A	A	A	A
59	693	G	A	A	A	.	A	A	A	A	A	A	A	.	A	A	A	A	A	.	A	A	A	A	A	A	.	.	A	A	A	A	A	-	A	A	A	A	A	A	A	A

Note: The column numbers (1–41) correspond to the S. No. assigned to the respective isolates in Table 1.

Supplementary Table S3: Variable sites in Cox 3 gene

	Samples

S. No	Vari	1	2	3	4	5	6	7	8	9	10	11	12	13	14	15	16	17	18	19	20	21	22	23	24	25	26	27	28	29	30	31	32	33	34	35	36	37	38	39	40	41
1	3	A	.	.	.	.	.	.	.	.	.	.	.	.	.	.	G	.	.	.	.	.	.	.	.	.	G	G	G	.	.	.	.	.	.	.	.	.	.	.	.	.
2	30	G	A	A	A	A	A	A	A	A	A	A	A	.	A	A	A	A	A	.	A	A	A	A	A	A	.	.	A	A	A	A	A	.	A	A	A	A	A	A	A	A
3	72	A	G	G	.	G	.	G	G	.	G	G	.	.	G	G	G	G	G	.	G	G	G	G	.	G	.	.	G	G	G	G	.	.	G	.	G	G	.	G	G	G
4	102	T	.	.	.	.	.	C	C	.	.	.	.	.	.	.	C	C	C	.	.	.	.	.	.	.	.	.	C	.	.	C	.	.	C	.	C	.	.	.	.	.
5	120	T	.	.	.	.	.	.	.	.	.	.	.	.	.	.	.	.	.	.	.	.	.	.	.	.	.	.	.	.	A	.	.	.	.	A	A	A	.	.	.	.
6	132	A	G	G	G	G	G	G	G	G	G	G	G	.	G	G	G	G	G	.	G	G	G	G	G	G	.	.	G	G	G	G	G	.	G	G	G	G	G	G	G	G
7	133	A	T	T	T	T	T	T	T	T	T	T	T	.	T	T	T	T	T	.	T	T	T	T	T	T	G	G	T	T	T	T	T	.	T	T	T	T	T	T	T	T
8	135	A	.	.	G	.	G	.	.	G	.	.	G	.	.	G	.	.	.	.	G	G	.	.	G	G	.	.	.	.	.	.	G	.	.	G	.	.	.	.	.	.
9	177	A	.	.	.	.	.	.	G	.	.	.	.	.	.	.	.	.	.	.	.	.	.	.	.	G	.	.	.	.	.	.	.	.	.	G	.	.	.	.	.	.
10	201	C	T	.	T	T	T	T	.	T	.	.	T	.	T	T	T	T	T	.	T	T	T	.	T	T	.	.	T	.	.	T	T	.	T	T	T	T	T	T	T	T
11	213	A	T	T	T	T	T	T	T	T	T	T	T	.	T	T	G	T	T	.	T	T	T	T	T	T	.	.	G	T	T	T	T	.	T	T	T	T	T	G	T	T
12	216	G	A	A	A	A	A	A	A	A	A	A	A	.	A	A	A	A	A	.	A	A	A	A	A	A	.	.	A	.	A	A	A	.	A	A	A	A	A	A	A	A
13	219	T	.	C	.	.	.	.	.	.	C	C	.	.	.	.	.	.	.	.	.	.	.	C	.	.	.	.	.	C	.	.	.	.	.	.	.	.	.	.	.	.
14	237	A	G	.	.	.	.	.	.	.	.	.	.	.	.	.	.	.	.	.	.	.	G	.	.	G	.	.	.	.	.	.	.	.	.	.	.	.	G	.	G	G
15	255	A	G	T	T	.	T	G	T	T	T	T	T	.	G	T	G	G	.	.	T	T	G	T	T	.	.	.	G	T	T	G	T	.	G	T	.	T	G	G	G	G
16	300	G	A	A	A	A	A	A	A	A	A	A	A	.	A	A	A	A	A	.	A	A	A	A	A	A	.	.	A	A	A	A	A	.	A	A	A	A	A	A	A	A
17	315	A	.	.	.	.	.	.	.	.	.	.	.	G	.	.	.	.	.	.	.	G	.	.	.	.	.	.	.	.	.	.	G	.	.	.	.	.	G	.	.	.
18	345	A	G	.	.	.	G	G	.	.	.	.	G	.	.	.	G	.	G	.	.	G	.	.	.	.	.	.	.	.	.	.	.	.	.	.	.	.	.	.	G	G
19	375	A	.	.	.	.	.	.	.	.	.	.	.	.	.	.	G	G	.	.	.	.	.	.	.	.	.	.	G	.	.	.	.	.	.	.	.	.	.	G	.	.
20	384	T	.	C	.	.	.	C	A	C	.	.	.	.	.	.	.	.	.	.	.	.	.	.	.	.	.	.	C	C	.	.	.	.	.	.	.	.	.	.	.	.
21	408	T	C	.	C	C	C	C	C	C	C	C	C	.	C	C	.	C	C	.	C	C	C	C	C	.	.	.	.	C	C	C	C	.	C	C	C	C	C	.	C	C
22	414	G	.	A	A	.	A	A	A	A	A	A	A	.	A	A	A	A	A	.	A	A	.	A	A	A	.	.	.	A	A	A	.	.	A	A	A	A	.	A	.	.
23	417	G	.	A	T	.	.	.	A	.	.	.	.	.	.	.	A	.	.	.	A	.	.	.	.	.	.	.	A	.	.	.	.	.	A	.	.	.	.	.	.	.
24	435	C	.	.	.	.	.	.	.	.	.	.	.	.	.	.	.	.	.	.	.	.	.	.	.	.	.	.	.	.	.	.	T	.	.	.	.	.	.	.	.	.
25	442	C	T	T	T	.	T	T	T	T	T	T	T	T	T	T	T	T	T	.	T	T	T	T	T	T	T	T	T	T	T	T	T	.	T	T	T	T	T	T	T	T
26	462	T	.	.	.	.	.	C	.	.	.	.	.	.	C	.	.	.	.	.	.	.	.	.	.	.	.	.	.	.	.	C	.	.	.	.	.	.	.	.	.	.
27	465	C	.	.	.	.	.	T	.	T	.	.	.	.	T	.	.	T	T	.	.	.	.	.	.	.	.	.	.	.	T	T	.	.	T	.	T	.	.	.	.	.
28	471	A	.	.	.	.	.	.	.	G	.	.	.	.	.	.	.	.	.	.	.	.	.	.	.	.	.	.	.	.	.	.	.	.	G	.	.	.	.	.	.	.

29	472	G	.	.	A	.	.	.	.	.	A	A	.	.	.	.	.	.	.	.	A	A	.	.	.	A	.	.	.	A	.	.	A	.	.	.	.	A	A	.	.	.
30	486	A	G	G	G	.	G	G	G	G	G	G	G	G	G	G	G	G	G	.	G	G	G	G	G	G	.	.	G	G	G	G	G	.	G	G	G	G	G	G	G	G
31	489	G	A	A	A	.	A	A	A	A	A	A	A	.	A	A	A	A	A	.	A	A	A	A	A	A	.	.	A	A	A	A	A	.	A	A	A	A	A	A	A	A
32	522	G	.	A	A	A	A	A	A	A	A	A	A	.	.	A	A	A	A	.	A	.	.	A	A	A	.	.	A	A	A	A	A	.	A	A	.	A	.	A	.	.
33	528	A	G	G	G	G	G	G	G	G	G	G	G	.	.	G	.	G	G	.	G	G	G	G	G	G	.	.	.	G	G	G	G	.	G	G	G	G	G	.	G	G
34	537	G	A	A	A	A	A	A	A	A	A	A	A	.	A	A	A	A	A	.	A	A	A	A	A	A	.	.	A	A	A	A	A	.	A	A	A	A	A	A	A	A
35	552	T	.	.	.	.	.	.	.	.	.	.	.	C	.	.	C	C	.	.	.	.	.	.	.	.	C	C	C	.	.	.	.	.	.	.	.	.	.	.	.	.
36	555	T	.	.	.	.	.	C	.	.	.	A	.	.	.	.	.	.	.	.	.	.	.	A	.	.	.	.	.	.	.	.	.	.	.	.	.	A	.	.	.	.
37	561	T	.	.	.	.	.	.	.	.	.	.	.	.	.	.	.	C	.	.	.	.	.	.	.	.	C	C	C	.	.	.	.	.	.	.	.	.	.	.	.	.
38	564	A	G	.	.	.	.	.	.	.	.	.	.	.	.	.	T	.	.	.	.	.	.	.	.	.	.	.	T	.	.	.	.	.	G	.	.	.	.	T	G	G
39	585	A	G	.	.	.	.	G	.	.	.	.	.	.	G	.	G	G	G	.	.	.	G	.	.	.	.	.	G	.	.	G	.	.	G	.	G	.	G	G	G	G
40	591	T	.	.	.	.	.	C	.	.	.	.	.	.	C	.	.	.	.	.	.	.	.	.	.	.	.	.	.	.	.	.	.	.	C	.	C	.	.	.	.	.
41	594	C	.	.	.	.	.	.	.	.	.	.	T	T	T	.	.	.	.	.	.	T	.	.	.	.	.	.	.	.	.	.	.	.	.	.	.	.	.	.	.	.
42	600	T	.	.	.	.	.	.	.	.	.	.	.	.	.	.	.	.	.	.	.	.	.	.	.	.	.	.	.	.	.	.	.	C	C	C	.	.	.	.	.	.
43	615	G	.	.	.	.	.	.	.	.	.	.	A	.	.	.	.	.	.	.	.	.	.	.	.	.	.	.	A	.	.	A	.	.	.	.	T	A	.	.	.	.
44	618	T	.	.	.	G	.	G	.	.	.	.	.	.	G	G	.	G	G	.	.	.	.	.	.	.	.	.	.	.	.	G	.	.	G	.	G	.	.	G	.	.
45	645	G	A	A	A	A	A	A	A	A	A	A	A	.	A	A	A	A	A	.	A	A	A	A	A	A	.	.	A	A	A	A	A	.	A	A	A	A	A	A	A	A
46	656	A	.	.	.	.	.	.	.	.	.	.	.	.	.	.	.	.	.	.	.	.	.	.	.	.	T	T	.	.	.	.	.	.	.	.	.	.	.	.	.	.
47	678	T	.	.	.	.	C	C	.	.	.	.	.	.	C	.	.	C	C	.	C	.	.	.	.	C	.	.	.	.	.	C	.	.	C	.	C	.	.	.	C	C
48	679	T	C	C	.	.	C	C	C	.	C	C	C	.	C	C	.	C	C	.	.	.	C	C	C	.	.	.	.	C	.	C	C	C	C	.	C	C	C	.	C	C
49	681	A	.	G	.	.	G	.	.	.	.	.	.	.	.	.	.	.	.	.	.	.	.	.	.	.	.	.	.	.	.	.	G	.	.	.	.	.	.	.	.	.
50	714	T	C	.	.	.	C	.	.	C	.	.	C	.	.	.	.	.	.	.	.	.	C	.	.	.	.	.	.	.	.	.	.	.	.	.	.	C	.	.	C	C
51	717	T	.	.	.	.	.	.	.	.	.	.	.	.	.	C	C	.	.	.	.	.	.	.	.	.	.	.	.	.	.	.	C	.	.	.	.	C	.	C	.	.
52	723	G	A	A	.	A	A	A	A	A	A	A	A	.	A	A	A	A	A	.	A	A	A	A	A	A	A	A	A	A	.	A	.	A	.	A	A	A	A	A	A	A
53	729	A	G	G	G	G	G	.	G	G	G	G	G	.	G	G	G	G	G	.	G	G	G	G	G	G	.	.	G	G	G	G	G	.	G	G	G	G	G	G	G	G
54	732	A	.	G	.	.	.	.	G	.	G	G	.	.	.	.	G	.	.	.	.	.	.	G	.	.	.	.	G	G	G	.	.	.	.	.	.	.	.	G	.	.
55	741	T	.	.	.	.	.	.	.	.	.	.	.	.	.	.	.	C	.	.	.	.	.	.	.	C	.	.	.	.	.	.	.	.	.	.	.	.	.	.	.	.
56	747	T	.	.	.	.	.	C	.	.	.	.	.	.	.	.	.	.	.	.	.	.	.	.	.	.	.	.	.	.	.	C	.	.	C	.	.	.	.	.	.	.
57	759	A	G	.	.	.	.	.	.	.	.	.	.	.	.	.	.	.	.	.	.	G	G	.	.	.	.	.	.	.	.	.	.	G	.	.	.	.	.	.	.	.

Note: The column numbers (1–41) correspond to the S. No. assigned to the respective isolates in Table 1.

Supplementary Table S4: Variable sites in Nad 1 gene

S. No	Samples																																									
	Vari	1	2	3	4	5	6	7	8	9	10	11	12	13	14	15	16	17	18	19	20	21	22	23	24	25	26	27	28	29	30	31	32	33	34	35	36	37	38	39	40	41
1	1	A	.	.	.	.	.	.	.	.	.	G	.	.	.	.	.	.	G	G	.	.	.	G	.	.	.	.	.	G	.	.	.	.	.	.	.	.	.	.	.	.
2	7	A	.	G	.	.	.	.	.	.	.	.	.	G	.	.	.	.	.	.	.	.	.	.	.	.	G	G	.	G	.	.	.	G	.	.	.	.	.	.	.	.
3	8	T	C	.	.	.	.	.	.	.	.	.	.	.	.	.	.	.	.	.	.	.	C	.	.	.	.	.	.	.	.	.	.	.	.	.	.	.	C	.	C	C
4	9	A	G	G	.	G	G	.	.	G	G	.	G	.	.	.	.	.	.	.	G	G	G	.	G	G	.	.	.	G	G	G	G	.	G	G	.	G	G	.	G	G
5	13	G	A	A	A	A	A	A	A	A	A	A	A	.	A	A	A	A	A	.	A	A	A	A	A	A	.	.	A	A	A	A	A	.	A	A	A	A	A	A	A	A
6	15	A	.	.	.	.	.	.	.	.	.	.	.	.	.	.	.	.	.	.	G	.	.	.	.	.	.	.	.	.	.	G	G	.	.	.	.	.	.	.	.	.
7	36	A	.	.	.	.	.	.	.	.	.	.	.	.	.	.	G	.	.	.	.	.	.	.	.	.	G	G	G	.	.	.	.	.	.	.	.	.	.	G	.	.
8	66	T	.	.	.	.	.	C	.	.	.	C	.	.	.	.	.	C	.	.	.	.	.	.	.	C	.	.	.	.	.	.	.	.	.	.	.	.	.	.	.	.
9	69	G	A	A	A	A	A	A	A	A	A	A	A	.	A	A	A	A	A	.	A	A	A	A	A	A	.	.	A	A	A	A	A	.	A	A	A	A	A	A	A	A
10	78	A	.	G	G	.	G	.	G	G	G	G	.	.	.	G	.	.	.	.	G	.	.	G	G	G	.	.	.	G	G	.	G	.	.	G	.	G	.	G	.	.
11	93	T	C	C	C	.	.	C	.	.	C	C	C	C	C	.	C	C	C	.	.	C	C	C	C	C	.	.	C	.	C	C	C	.	C	.	C	.	C	.	C	C
12	96	T	.	C	C	.	C	C	C	.	C	C	C	.	C	.	.	C	C	.	.	C	C	C	C	C	.	.	.	C	C	C	.	.	.	.	C	C	C	.	C	C
13	102	A	G	G	G	G	G	G	G	G	G	G	G	G	G	G	.	.	G	.	G	G	G	G	G	G	.	.	.	G	G	G	G	.	G	G	G	G	G	G	G	G
14	117	A	.	.	.	.	G	.	G	.	.	.	.	.	.	.	.	.	.	.	T	G	G	.	.	G	.	.	.	.	G	G	G	.	G	.	T	.	.	G	G	G
15	123	A	G	G	G	G	G	G	G	G	G	G	G	.	G	G	G	G	G	.	G	G	G	G	G	.	.	.	G	G	G	G	G	.	G	.	G	.	.	G	G	G
16	126	A	.	.	.	.	.	.	.	.	.	.	G	.	.	.	.	.	.	.	G	.	.	G	.	.	.	.	.	.	.	.	.	.	.	.	.	.	.	.	.	.
17	138	G	A	A	A	.	A	A	A	A	T	T	A	.	A	A	.	A	A	.	A	A	.	T	A	.	.	.	.	A	A	.	A	A	A	A	A	.	A	A	A	A
18	156	A	.	.	.	.	.	.	.	.	.	.	G	.	.	G	G	.	.	.	.	.	.	.	.	G	.	.	G	.	.	.	.	.	G	.	.	G	.	.	.	.
19	162	G	T	T	T	T	T	T	T	T	T	T	T	.	T	T	T	T	T	.	T	T	T	T	T	T	.	.	T	T	T	T	T	.	T	T	T	A	T	T	T	T
20	165	T	A	A	G	A	A	A	A	G	A	A	A	.	G	A	A	.	G	.	A	A	A	A	A	A	.	.	A	A	A	A	A	.	G	A	A	A	A	A	.	.
21	177	G	A	A	A	.	A	A	A	A	A	A	A	.	A	A	A	A	A	.	A	A	A	A	A	A	.	.	A	A	A	A	A	.	A	A	A	A	A	A	A	A
22	186	A	.	.	G	.	.	.	.	.	.	.	.	.	G	.	.	G	G	.	.	.	.	.	.	.	.	.	.	.	.	G	.	.	G	.	G	.	.	G	.	.

23	192	G	.	.	.	.	A	.	.	.	.	.	.	.	A	.	.	.	.	.	.	.	.	.	.	.	.	.	.	.	.	.	.	.	.	.	.	A	.	.	.	.
24	195	G	A	A	A	.	A	A	A	A	A	A	A	.	A	A	A	A	A	.	A	A	A	A	A	A	.	.	A	A	A	A	.	.	A	A	A	A	A	A	A	A
25	204	G	A	A	A	.	A	A	A	A	A	A	A	.	A	A	A	A	A	.	A	.	A	A	.	A	.	.	A	A	A	A	.	A	A	A	A	A	A	A	A	A
26	207	A	G	G	G	.	G	G	G	G	G	.	G	.	G	G	G	G	G	.	G	G	G	G	G	.	.	.	G	G	G	.	G	.	G	G	G	G	G	G	G	G
27	216	A	G	G	G	.	G	G	G	G	G	G	G	.	G	G	G	G	G	.	G	G	G	G	G	G	.	.	G	G	G	G	G	.	G	G	G	G	G	G	G	G
28	231	T	.	C	C	C	.	.	C	C	C	C	C	C	.	.	C	.	.	.	C	.	C	C	C	.	C	C	.	C	C	.	.	.	.	C	.	C	.	.	C	C
29	240	G	A	A	A	.	A	A	A	A	A	A	A	.	A	A	A	A	A	.	A	A	A	A	A	A	.	.	A	A	A	A	A	.	A	A	A	A	A	A	A	A
30	246	G	.	.	.	A	.	.	.	.	.	.	.	A	.	.	.	.	.	.	.	.	.	.	.	.	A	A	.	.	.	.	.	.	.	.	.	.	.	.	.	.
31	261	A	.	.	.	G	.	.	.	.	.	.	.	G	.	.	.	.	.	.	.	.	.	.	.	.	G	G	.	.	.	.	.	G	.	.	.	.	.	.	.	.
32	264	G	A	A	A	A	A	A	A	A	A	A	A	A	A	.	.	A	A	A	.	A	A	A	A	A	A	A	.	A	A	A	A	A	A	.	A	A	A	.	A	A
33	267	T	C	C	C	.	C	C	C	.	C	C	C	.	C	C	C	.	C	.	C	C	C	C	.	C	.	.	C	C	C	.	C	.	.	C	C	C	C	.	C	C
34	273	G	A	A	A	.	A	A	A	A	A	A	A	.	A	A	A	A	A	.	.	A	A	A	A	A	.	.	A	A	A	A	A	.	A	A	A	A	A	A	A	A
35	287	G	A	A	A	.	A	A	A	A	A	A	A	.	A	A	A	A	A	.	A	A	A	A	A	A	.	.	A	A	A	A	A	.	A	A	A	A	A	A	A	A
36	292	G	A	A	A	.	A	A	A	A	A	A	A	.	A	A	A	A	A	.	A	A	A	A	A	A	.	.	A	A	A	A	A	.	A	A	A	A	A	A	A	A
37	330	A	.	.	.	G	.	.	.	.	.	.	.	G	.	.	.	.	.	.	.	.	.	.	.	.	.	.	.	.	.	.	.	G	.	.	.	.	.	.	.	.
38	336	G	A	A	A	.	A	A	A	A	A	A	A	.	A	A	.	A	A	.	A	A	A	A	A	A	.	.	.	A	A	A	A	.	A	A	A	A	A	.	A	A
39	339	T	.	.	.	C	.	.	.	.	.	.	.	.	.	.	.	.	.	.	.	.	.	.	.	.	C	C	.	.	.	.	.	.	.	.	.	.	.	.	.	.
40	348	C	.	T	.	.	.	T	.	T	T	.	.	.	T	.	T	T	T	.	.	.	T	.	T	.	T	T	.	T	.	T	.	.	T	.	T	.	.	.	.	.
41	351	T	.	.	.	.	.	.	.	.	.	.	.	.	.	.	.	.	.	.	.	.	.	.	.	C	.	.	.	.	.	.	C	.	.	C	.	.	.	C	.	.
42	366	G	.	.	.	.	.	.	.	.	.	.	.	A	.	.	.	.	.	.	.	.	.	.	.	.	.	.	.	.	.	.	.	.	.	.	.	.	.	.	.	.
43	369	G	.	.	.	.	.	A	A	.	.	A	.	A	A	.	.	A	A	.	.	.	.	.	.	.	A	A	A	.	.	A	.	.	.	.	A	.	.	.	.	.
44	372	G	.	A	A	.	A	A	A	A	A	A	.	.	A	A	A	.	.	A	A	.	.	A	A	.	A	A	.	A	.	A	A	A	A	.	.	.	.	A	A	A
45	378	A	G	G	G	G	G	G	G	G	G	.	G	.	.	G	G	G	G	.	G	G	G	.	G	G	G	G	G	G	G	G	G	.	G	.	G	G	G	G	G	G

46	390	A	.	.	.	.	G	G	.	.	.	G	G	.	.	G	.	.	G	.	.	.	G	G	.	G	.	.	G	.	G	G	.	.	.	.	.	.	.	.	.	.
47	420	A	G	G	G	G	G	.	G	G	G	G	G	.	G	G	.	G	G	.	G	G	G	G	G	G	.	.	G	G	G	G	.	.	G	G	G	G	G	.	G	G
48	429	T	A	A	A	A	A	A	A	A	A	A	A	.	A	A	A	A	A	.	A	A	A	A	A	A	.	.	A	A	A	A	A	.	A	A	A	A	A	A	A	A
49	457	T	.	.	.	.	.	.	.	.	.	.	.	C	.	.	.	.	.	C	.	.	.	.	.	.	.	.	.	.	.	.	.	C	.	.	.	.	.	.	.	.
50	460	T	A	A	A	A	A	A	A	A	A	A	A	.	A	A	A	A	A	.	A	A	A	A	A	A	.	.	A	A	A	A	A	.	A	A	A	A	A	A	A	A
51	462	T	.	.	.	.	.	.	.	.	.	.	.	G	.	.	.	.	.	.	.	.	.	.	.	.	A	A	.	.	.	.	.	A	.	.	.	.	.	.	.	.
52	468	G	A	A	A	A	A	A	A	A	A	A	A	.	A	A	A	A	A	.	A	A	A	A	A	A	.	.	A	A	A	A	A	.	A	A	A	A	A	A	A	A
53	471	A	T	T	T	T	T	T	T	T	T	T	T	.	T	T	.	T	T	G	T	T	T	T	T	.	G	G	.	T	T	T	T	G	T	T	.	.	T	.	T	T
54	474	C	T	T	T	T	T	T	T	T	T	T	T	.	T	T	T	T	.	.	T	.	T	T	T	T	T	T	T	T	T	T	T	T	T	T	T	T	T	T	T	T
55	480	A	G	G	G	G	G	G	G	G	G	G	G	.	G	G	G	G	G	.	G	G	G	G	G	G	.	.	G	G	G	G	G	G	G	G	G	.	G	.	G	G
56	513	A	T	T	T	T	T	T	T	T	T	T	T	.	T	T	T	T	T	.	T	T	T	T	T	T	.	.	T	T	T	T	T	.	T	T	T	T	T	T	T	T
57	516	G	.	.	.	.	.	.	.	.	.	.	.	A	.	.	.	.	.	.	.	.	.	.	.	.	A	A	.	.	.	.	.	A	.	.	.	.	.	.	.	.
58	543	A	.	.	G	G	.	.	.	.	.	.	G	.	.	.	G	.	.	G	G	.	G	.	.	G	G	G	.	.	.	G	.	.	.	.	.	.	G	G	.	.
59	546	A	.	G	G	.	G	G	G	G	G	G	G	.	G	.	.	G	.	.	.	G	.	.	G	G	.	.	.	G	.	G	G	.	.	G	.	G	.	G	.	.
60	547	A	.	G	.	.	.	.	.	.	.	.	.	.	.	.	.	.	.	.	.	.	.	.	.	.	G	G	.	.	.	.	.	.	.	.	.	.	.	.	.	.
61	552	T	.	.	.	C	.	.	.	.	.	.	.	C	.	.	.	.	.	.	.	.	.	.	.	.	C	C	.	.	.	.	.	.	.	.	.	.	.	.	.	.
62	561	A	G	G	G	.	G	G	G	G	G	G	G	.	G	G	G	G	G	.	G	G	G	G	G	G	.	.	G	G	G	G	G	.	G	.	G	G	.	G	G	G
63	585	T	.	C	.	C	.	C	.	.	C	C	.	.	C	.	.	.	.	.	.	.	.	C	C	.	.	.	C	C	C	C	C	C	C	C	C	.	.	C	.	.
64	588	A	G	G	G	.	.	G	G	G	G	G	G	.	G	G	G	G	G	.	G	G	G	G	G	G	.	.	.	G	G	G	G	.	G	G	G	.	G	.	G	G
65	603	A	G	G	G	.	.	G	G	G	G	G	.	.	G	.	G	G	G	.	G	G	G	G	G	G	.	.	G	G	G	G	G	.	G	G	G	.	G	.	G	G
66	606	T	.	.	.	.	.	.	.	.	.	.	.	.	.	.	.	.	C	.	.	.	.	.	C	.	C	C	.	.	.	.	.	.	.	.	.	.	.	.	.	.
67	609	G	A	A	A	.	A	A	A	A	A	A	A	.	A	A	A	A	A	.	A	A	A	A	A	A	.	.	A	A	A	A	A	.	A	A	A	A	A	A	A	A
68	621	A	.	.	.	G	.	G	.	G	.	.	G	G	G	.	G	G	G	G	.	.	G	.	G	G	G	G	G	.	.	G	G	G	G	.	G	.	.	G	.	.

69	630	A	G	G	G	G	.	G	G	.	.	G	G	G	G	G	.	G	G	.	.	G	.	G	G	.	.	.	.	G	.	.	G	G	G	.	G	G	.	G	.	.
70	633	G	A	A	A	.	A	A	A	A	A	A	A	A	A	A	.	A	A	.	A	A	A	A	A	A	.	.	.	A	A	A	A	.	A	A	A	A	A	A	A	A
71	639	T	G	G	G	.	G	G	G	G	G	G	G	.	G	G	.	G	G	.	G	G	G	G	G	G	.	.	.	G	G	G	G	.	G	G	G	G	G	.	G	G
72	651	G	A	A	A	.	A	A	A	A	A	A	A	.	A	A	A	A	A	.	A	A	A	A	A	A	.	.	A	A	A	A	A	.	A	A	A	A	A	A	A	A
73	657	A	.	.	.	.	.	.	.	.	.	.	.	.	.	G	G	.	.	.	.	.	.	.	.	.	.	.	G	.	G	.	.	.	G	.	G	.	.	G	.	.
74	660	A	.	G	.	G	.	.	.	.	.	G	.	G	.	.	.	.	.	G	.	.	.	G	.	.	G	G	.	G	.	.	.	G	.	.	.	.	.	.	.	.
75	663	G	A	A	A	.	A	A	A	A	A	A	A	.	A	A	A	A	A	.	A	A	A	A	A	A	.	.	A	A	.	A	A	.	A	A	A	A	A	A	A	A
76	669	G	A	.	.	.	.	.	A	.	.	.	.	.	A	A	.	.	.	A	.	.	.	.	.	A	.	.	A	.	.	A	.	.	A	A	.	.	.	.	A	A
77	670	A	.	.	.	.	.	.	.	.	.	.	G	.	.	.	.	G	G	.	G	.	G	.	.	.	.	.	.	.	.	.	.	.	.	.	.	.	.	.	G	G
78	699	A	.	.	.	.	.	.	.	.	.	.	.	G	.	.	.	.	.	.	.	.	.	.	.	.	.	.	.	.	.	G	.	.	.	.	.	.	.	G	.	.
79	702	A	G	G	G	.	G	.	.	G	G	G	.	.	.	G	G	.	.	.	.	G	.	.	.	.	.	.	G	G	.	.	G	.	.	.	.	G	G	.	G	G
80	714	T	A	A	A	.	A	A	A	A	A	A	A	.	A	A	A	A	A	.	A	A	A	A	A	A	.	.	A	A	A	A	A	.	A	A	A	A	A	A	A	A
81	724	G	A	A	A	.	A	A	A	A	A	A	A	.	A	A	A	A	A	.	A	A	A	A	A	A	.	.	A	A	A	A	A	.	A	A	A	A	A	A	A	A
82	729	A	.	.	.	G	.	.	G	.	.	.	.	G	.	G	.	.	.	.	.	.	.	.	.	.	G	G	.	.	.	.	.	G	.	.	.	.	.	.	.	.
83	730	A	.	.	.	.	.	.	.	.	.	.	.	G	.	.	.	.	.	.	.	.	.	.	.	.	G	G	.	.	.	.	.	.	.	.	.	.	.	.	.	.
84	732	G	.	.	.	.	.	.	.	.	.	.	.	.	.	.	A	.	.	.	.	.	.	.	.	.	.	.	A	.	.	.	.	.	.	.	.	.	.	A	.	.
85	765	A	T	T	T	.	T	T	T	T	T	T	T	.	T	T	T	T	T	.	T	T	T	T	T	T	.	.	T	T	T	T	T	.	T	T	T	T	T	T	T	T
86	777	G	T	T	T	.	T	T	T	T	T	T	T	T	T	T	T	T	T	.	T	T	T	T	T	T	A	A	T	T	T	T	T	.	T	T	T	T	T	T	T	T
87	780	A	.	G	.	.	.	.	G	.	G	G	.	.	.	.	.	.	.	.	.	.	.	G	.	.	.	.	.	G	G	.	.	.	.	.	.	.	G	.	.	.
88	789	C	T	T	T	.	T	.	T	T	T	T	T	T	.	T	T	.	.	.	T	T	T	T	T	T	T	T	T	T	T	.	T	.	.	T	.	.	T	T	T	T
89	795	A	.	.	.	.	G	.	.	.	.	.	.	.	.	.	.	.	.	.	.	.	.	.	.	.	G	G	G	.	.	.	.	.	.	.	.	.	.	.	.	.
90	798	A	G	.	.	.	G	.	.	.	.	.	.	.	.	.	.	.	.	.	.	.	.	.	.	.	.	.	.	.	.	.	.	.	.	.	.	.	.	.	G	G
91	804	A	.	.	.	.	.	.	.	.	.	.	.	.	.	.	.	.	.	.	.	.	.	.	.	.	.	.	.	.	.	G	.	G	.	.	.	.	.	G	.	.

92	864	G	A	A	A	A	A	A	A	A	A	A	A	.	A	A	.	A	A	.	A	A	A	A	A	A	.	.	A	A	A	A	A	.	A	A	A	A	A	A	A	A
93	867	G	A	A	A	A	A	A	A	A	A	A	A	.	A	A	A	A	A	.	A	A	A	A	A	A	.	.	A	A	A	A	A	.	A	A	A	A	A	A	A	A
94	873	A	.	.	.	.	.	G	.	.	.	.	.	G	G	.	.	.	G	.	.	.	.	.	.	.	.	.	.	.	.	G	.	.	.	.	.	.	.	.	.	.

Note: The column numbers (1–41) correspond to the S. No. assigned to the respective isolates in Table 1.

Supplementary Table S5: Variable sites in the Nad 2 gene

S. No	Samples																																									
	Vari	1	2	3	4	5	6	7	8	9	10	11	12	13	14	15	16	17	18	19	20	21	22	23	24	25	26	27	28	29	30	31	32	33	34	35	36	37	38	39	40	41
1	45	A	G	G	G	.	G	G	G	G	G	G	G	.	G	G	G	G	G	.	G	G	G	G	G	G	.	.	G	G	G	G	G	.	G	G	G	.	G	G	G	G
2	49	A	G	G	G	.	G	G	G	.	G	G	G	.	G	G	G	G	G	.	G	G	.	G	.	G	.	.	G	.	G	G	G	G	G	.	G	G	G	.	.	.
3	50	C	T	T	T	.	T	T	T	T	T	T	T	T	T	T	T	T	T	.	T	T	T	T	T	T	.	.	T	T	T	T	T	T	T	T	T	T	T	T	T	T
4	54	A	G	.	.	.	.	G	.	G	.	.	.	.	G	.	.	G	G	.	.	.	G	.	.	.	.	.	.	.	.	G	.	.	G	.	G	.	G	G	G	G
5	57	A	.	.	G	.	.	T	.	G	.	G	.	.	.	G	.	.	.	.	.	.	.	G	G	.	.	.	.	.	.	.	.	.	.	.	G	G	.	.	G	G
6	66	A	.	.	G	.	.	.	.	.	.	.	.	.	.	G	.	G	G	.	.	G	.	.	.	.	.	.	.	.	.	.	.	.	.	.	.	.	.	.	.	.
7	67	A	.	.	.	.	.	T	.	.	.	.	.	.	T	.	.	T	T	.	.	.	.	.	.	.	.	.	.	.	.	T	.	.	T	.	T	.	.	.	T	T
8	75	G	.	A	.	.	.	.	.	A	A	.	.	A	.	.	.	.	.	.	.	.	.	.	.	A	A	A	.	.	.	.	A	A	.	.	.	.	.	.	.	.
9	78	A	.	.	.	.	.	.	.	.	G	.	.	.	.	.	.	.	.	.	.	.	.	.	.	G	G	G	.	.	.	.	.	G	.	.	.	.	.	.	.	.
10	93	G	A	A	.	A	A	A	A	.	A	A	A	.	A	A	A	A	A	.	A	A	A	A	A	A	A	A	A	A	A	A	A	A	A	A	A	A	A	A	A	A
11	96	A	.	.	.	.	.	G	.	.	.	.	.	.	G	.	.	G	.	.	.	.	.	.	.	.	.	.	G	.	.	G	.	.	G	.	G	.	.	.	.	.
12	102	G	A	A	A	A	A	A	A	A	A	A	A	.	A	A	.	A	A	.	A	A	A	A	A	A	.	.	.	A	A	A	A	.	A	A	A	.	A	.	A	A
13	109	G	.	.	.	.	.	.	.	.	.	.	.	.	.	.	A	A	A	.	.	.	.	.	.	.	.	.	A	.	.	A	.	.	.	.	.	.	.	A	.	.
14	111	T	.	.	.	.	.	.	.	.	.	.	.	A	.	.	.	.	.	.	.	A	.	.	.	.	A	A	.	.	.	.	.	A	C	.	C	.	.	.	.	.
15	117	T	.	.	.	.	.	.	.	.	.	.	.	.	A	.	.	.	.	.	.	.	.	.	.	.	.	.	.	.	.	.	.	.	.	.	.	A	.	.	.	.
16	130	A	.	.	.	.	.	.	.	.	.	.	.	.	.	.	.	.	.	.	.	.	.	.	.	.	G	G	.	.	.	.	.	G	.	.	.	.	.	.	.	.
17	141	A	.	G	T	G	G	.	G	.	G	.	.	G	.	G	.	.	.	T	.	.	G	.	.	.	T	T	.	.	G	.	.	T	.	.	G	.	.	T	.	.
18	147	A	.	.	.	.	.	.	.	.	.	.	.	.	.	.	.	.	.	G	.	.	.	.	G	G	.	.	.	.	.	.	.	G	.	.	.	.	.	G	.	.
19	171	A	.	.	.	.	.	.	.	G	.	.	.	.	.	.	G	.	.	.	.	.	.	.	.	.	.	.	G	.	.	.	.	.	.	.	.	.	.	.	.	.
20	177	A	.	.	.	.	.	.	.	.	.	.	.	.	.	.	.	.	.	.	.	.	.	.	.	.	.	.	.	G	.	.	.	.	.	G	.	G	.	.	.	.
21	184	A	.	.	.	T	.	.	.	.	.	.	.	.	.	.	.	.	.	.	T	.	.	.	.	.	.	.	.	.	.	.	.	.	.	T	.	.	.	.	.	.

22	195	A	.	.	G	.	.	.	.	.	.	.	.	.	.	.	.	.	.	.	.	.	.	.	.	G	.	.	.	.	.	.	.	.	.	.	.	.	.	.	.	.
23	198	A	.	.	.	.	G	.	.	G	.	.	.	.	.	.	.	.	G	.	.	G	.	.	.	.	.	.	G	.	.	.	G	.	.	.	.	.	.	G	.	.
24	199	A	.	.	.	.	.	G	.	.	.	.	.	.	.	.	G	.	.	.	.	.	.	.	.	.	.	.	G	.	.	.	.	.	.	.	.	.	.	G	.	.
25	211	A	.	.	G	.	.	.	.	.	.	.	.	.	G	.	.	.	.	.	.	.	.	.	.	.	.	.	G	.	.	.	.	.	.	.	.	.	.	.	.	.
26	219	A	.	.	.	G	.	.	.	.	.	.	.	.	.	.	.	.	.	.	G	.	.	.	.	.	.	.	.	.	.	.	.	.	.	G	.	.	.	.	.	.
27	222	A	G	.	G	.	G	.	.	G	G	.	G	.	.	.	.	.	.	.	.	.	G	.	G	G	.	.	G	.	.	T	G	.	.	.	.	G	.	G	G	G
28	231	A	G	G	.	G	.	.	G	.	G	G	.	.	.	G	.	.	.	.	G	G	.	G	G	G	.	.	.	G	G	.	G	.	.	G	.	G	.	.	G	G
29	240	T	.	.	.	.	.	.	.	.	.	.	.	.	.	.	C	C	.	.	.	.	.	.	.	.	C	C	.	.	.	.	.	.	.	.	.	.	.	.	.	.
30	243	G	A	A	A	A	A	A	A	.	A	A	A	T	A	A	A	.	.	.	.	A	A	A	A	A	.	.	A	A	A	A	A	.	A	A	A	A	A	A	A	A
31	282	T	.	.	.	.	.	.	.	.	.	.	.	.	.	C	.	.	.	.	C	.	.	.	.	.	.	.	.	.	.	.	C	.	.	C	.	.	.	.	.	.
32	284	A	.	.	.	.	.	G	.	.	.	.	.	.	.	.	.	.	.	.	.	G	.	.	.	.	.	.	.	.	.	.	.	.	.	.	.	G	.	G	G	G
33	288	T	.	.	.	.	.	C	.	.	.	.	.	.	.	.	C	.	.	.	.	.	.	.	.	.	.	.	C	.	.	.	.	.	.	.	.	.	.	.	.	.
34	291	C	.	.	.	.	T	T	.	.	T	.	.	T	.	.	.	T	T	.	.	.	.	T	.	T	T	T	.	.	.	.	.	.	.	.	.	T	.	.	.	.
35	295	A	T	T	T	T	T	T	T	T	T	T	T	.	T	T	T	T	T	.	T	T	T	T	T	T	.	.	T	T	T	T	T	.	T	T	T	T	T	T	T	T
36	297	G	.	.	A	.	.	A	.	.	A	A	.	A	.	.	A	.	.	.	.	.	A	A	A	A	A	A	A	.	.	A	.	A	.	.	.	.	.	A	A	A
37	309	A	G	G	G	G	G	G	G	G	G	G	G	.	G	G	G	G	G	.	G	G	G	G	G	G	.	.	G	G	G	G	.	.	G	G	G	G	G	G	G	G
38	327	G	A	A	A	A	A	A	A	A	A	A	A	.	A	A	A	A	A	.	A	A	A	A	A	A	.	.	A	A	A	A	A	.	A	A	A	A	A	.	A	A
39	346	T	.	.	.	.	.	.	.	.	.	.	.	.	C	.	.	C	C	.	.	.	.	.	.	.	.	.	.	.	.	.	.	.	C	.	C	.	.	.	.	.
40	351	A	.	.	.	.	.	.	.	.	.	.	.	.	G	.	G	G	.	.	.	.	.	.	.	.	.	.	.	.	.	G	.	.	G	.	G	.	G	.	.	.
41	368	A	G	.	.	.	.	G	.	.	.	.	.	.	G	.	G	G	G	.	.	.	G	.	.	.	.	.	G	.	.	G	.	.	G	.	G	.	G	G	.	.
42	370	A	G	G	G	G	G	.	G	G	G	G	G	.	G	G	.	G	G	.	G	G	G	G	G	G	.	.	.	G	G	G	G	.	G	G	G	G	G	.	.	.
43	375	A	G	.	.	.	.	.	.	.	.	.	.	G	.	.	.	.	.	.	.	.	.	.	.	.	G	G	.	.	.	.	.	G	.	G	.	.	.	.	.	.
44	387	A	G	G	G	G	.	G	G	G	G	G	G	.	G	G	.	G	G	.	G	G	G	G	G	G	.	.	.	G	G	G	G	.	G	G	G	.	G	.	G	G
45	388	A	G	G	G	G	.	G	G	G	.	G	G	.	G	G	G	G	G	.	G	G	G	G	G	G	.	.	G	G	G	G	G	.	G	G	G	G	G	G	G	G
46	389	T	.	.	.	C	.	C	.	C	.	.	.	.	C	C	C	C	C	.	C	.	C	.	.	.	.	.	C	.	.	C	C	.	C	C	C	.	C	C	C	C
47	390	G	.	.	.	.	.	A	.	A	.	.	.	.	A	.	.	A	A	.	.	.	.	.	.	.	.	.	A	.	.	.	.	.	A	.	.	.	.	A	A	A
48	399	A	G	G	G	G	G	G	G	G	G	G	G	.	G	G	G	G	G	.	G	G	G	G	G	G	.	.	G	G	G	G	G	G	G	G	G	G	G	G	G	G
49	405	T	C	.	C	.	C	C	C	C	C	C	C	.	C	C	C	C	C	.	.	C	.	C	C	C	.	.	C	.	.	C	.	.	C	.	C	C	C	.	.	.
50	420	A	G	G	.	.	G	.	G	G	G	G	G	G	.	G	G	.	.	.	.	G	.	G	G	G	.	.	.	G	G	.	G	.	.	.	.	G	G	G	G	G
51	425	C	T	T	T	.	T	T	T	T	T	T	T	.	T	T	T	T	T	.	T	T	T	T	T	T	.	.	T	T	T	T	T	.	T	T	T	T	T	T	T	T

52	459	A	T	T	T	.	T	T	C	T	C	T	T	T	T	T	T	T	T	.	T	T	T	T	T	T	.	.	T	T	C	T	T	.	T	T	T	T	T	T	T	T
53	462	T	A	A	A	.	A	A	A	A	A	A	A	.	A	A	A	A	A	.	A	A	A	A	A	A	.	.	A	A	A	A	A	.	G	A	A	A	A	A	A	A
54	483	A	G	G	G	.	G	G	G	G	G	G	G	.	G	.	G	G	G	.	G	.	G	G	G	G	.	.	G	G	G	.	.	.	G	G	G	G	G	.	G	G
55	486	A	G	G	G	.	G	G	G	G	G	G	G	G	G	.	.	G	.	.	.	G	G	G	G	G	.	.	G	G	G	G	.	G	.	.	.	.	.	.	G	G
56	513	A	.	.	.	.	.	.	.	.	.	.	.	G	.	.	G	.	.	.	.	.	.	.	.	.	.	.	G	.	.	.	.	.	.	.	.	.	.	G	.	.
57	514	A	G	G	G	.	G	G	G	G	G	G	G	.	G	G	.	G	G	.	G	G	G	G	G	G	.	.	.	G	G	G	G	.	G	G	G	G	G	G	G	G
58	522	G	T	T	T	.	T	T	T	T	T	T	T	.	T	T	T	T	T	.	T	T	T	T	T	T	.	.	T	T	T	T	T	.	T	T	T	T	T	T	T	T
59	549	A	G	.	.	.	.	G	.	.	.	.	.	G	G	G	G	G	G	.	G	.	G	.	.	.	.	.	G	.	.	G	G	G	G	G	G	.	G	G	G	G
60	568	A	G	G	G	.	G	G	G	G	G	G	G	.	G	G	G	G	G	.	G	G	G	G	G	G	.	.	G	G	G	G	G	.	G	G	G	G	G	G	G	G
61	569	G	C	C	C	.	C	C	C	C	C	C	C	.	C	C	T	C	C	.	C	C	C	C	C	C	.	.	T	C	C	C	C	.	C	T	T	C	C	T	C	C
62	570	A	.	.	.	.	.	.	.	.	.	.	.	.	G	.	.	.	.	.	T	.	.	.	.	.	.	.	.	.	.	.	.	.	.	.	G	.	.	.	.	.
63	603	G	A	A	A	.	A	A	A	A	A	A	A	.	A	A	A	A	A	.	A	A	A	A	A	A	.	.	A	A	A	A	A	.	A	A	A	A	A	A	A	A
64	612	A	.	.	.	.	.	G	.	G	.	.	.	.	G	.	.	G	G	.	.	.	.	.	.	G	.	.	.	.	.	G	.	.	G	.	G	G	.	.	.	.
65	613	G	A	A	A	.	A	.	A	A	A	.	A	.	.	A	.	.	.	.	A	A	A	A	A	A	.	.	.	A	A	.	A	.	.	A	.	A	A	.	A	A
66	627	A	G	G	G	.	G	G	G	G	G	G	G	.	G	G	G	G	G	.	G	G	G	G	G	G	.	.	G	G	G	G	G	.	G	G	G	G	G	G	G	G
67	630	G	.	.	.	.	A	.	A	.	.	.	.	.	.	T	.	.	.	.	.	A	.	.	.	.	.	.	.	.	.	.	A	A	.	.	.	.	.	.	.	.
68	633	G	A	A	A	.	A	A	A	A	A	A	A	.	A	A	A	A	A	.	A	A	A	A	A	A	.	.	A	A	A	A	A	.	A	A	A	A	A	A	A	A
69	636	A	.	G	G	.	G	.	G	G	G	G	G	.	.	G	.	.	.	.	G	G	.	G	G	G	.	.	.	G	G	.	G	.	.	G	.	G	.	.	.	.
70	673	G	A	A	A	.	A	A	A	A	A	A	A	.	A	A	A	A	A	.	A	A	A	A	A	A	.	.	A	A	A	A	A	.	A	A	A	A	A	A	A	A
71	678	A	.	.	.	.	.	.	.	.	.	.	.	.	G	.	.	.	G	.	.	G	.	.	.	.	G	G	.	.	.	.	.	.	.	.	.	.	.	.	.	.
72	697	A	G	G	G	.	G	G	G	G	G	G	G	.	G	G	G	G	G	.	G	G	G	G	G	G	.	.	G	G	G	G	G	.	G	G	G	G	G	G	G	G
73	756	T	.	.	C	C	C	.	.	.	.	.	.	.	.	.	C	.	.	.	C	C	.	.	.	.	.	.	C	.	.	.	C	.	C	C	C	C	.	.	.	.
74	778	A	.	.	.	.	.	.	.	.	G	.	G	G	.	.	.	.	.	.	.	.	.	.	.	.	.	.	.	.	.	.	.	.	.	.	.	.	.	.	.	.
75	786	C	T	.	T	T	T	T	T	T	T	T	T	T	T	T	T	T	T	.	T	T	T	T	T	T	.	.	T	T	T	T	T	.	T	T	T	T	T	T	T	T
76	794	A	G	.	.	G	G	G	G	G	G	G	G	.	G	G	G	G	G	.	G	G	G	G	G	G	.	.	G	G	G	G	G	.	G	G	G	.	G	.	G	G
77	795	T	C	C	C	C	C	C	C	C	C	C	.	.	C	C	C	C	C	.	C	C	C	C	C	C	.	.	C	C	C	C	C	.	C	C	C	C	C	C	C	C
78	806	A	.	.	.	.	.	G	.	.	.	.	.	.	.	.	.	.	.	.	.	.	.	.	.	.	.	.	.	.	.	.	T	.	.	.	.	.	.	.	.	.
79	808	T	A	A	A	A	A	A	A	A	A	A	A	.	A	A	A	A	A	.	A	A	A	A	A	A	.	.	A	A	A	A	A	.	A	A	A	A	A	A	A	A
80	810	A	G	G	G	G	G	G	G	G	G	G	G	.	G	G	G	G	G	.	G	G	G	G	G	G	.	.	G	G	G	G	G	.	G	G	G	G	G	G	G	G
81	813	T	.	.	.	.	A	A	.	.	.	.	.	.	.	A	A	.	.	.	.	.	.	.	.	.	.	.	.	.	.	.	.	.	.	.	.	.	.	.	.	.

82	814	A	G	.	G	G	G	G	G	G	G	G	G	.	G	G	G	G	G	.	G	G	G	G	G	G	.	.	G	.	G	G	G	.	G	G	G	G	G	.	G	G
83	819	G	.	.	.	.	.	.	.	.	.	.	.	.	.	.	.	.	.	A	.	A	.	.	.	.	.	.	.	.	.	.	.	.	A	.	.	.	.	.	.	.
84	828	A	G	G	.	.	.	G	G	.	G	G	.	.	G	.	.	G	G	.	.	G	G	G	.	.	.	.	G	G	G	G	.	.	G	.	G	.	G	G	G	G
85	829	A	.	G	.	.	.	.	.	.	.	.	.	.	.	G	.	G	G	.	.	.	.	.	.	.	.	.	.	.	G	.	G	.	.	.	.	.	.	.	.	.
86	830	C	.	T	.	.	.	.	.	.	.	.	.	.	.	T	.	T	T	.	.	.	.	.	.	.	.	.	T	.	.	.	.	.	.	T	.	.	.	T	.	.
87	831	A	.	.	.	.	.	.	.	.	.	.	.	.	.	.	.	G	G	.	.	.	.	G	.	.	.	.	.	.	G	.	.	.	.	.	.	.	.	.	.	.
88	837	A	.	.	.	.	.	.	.	.	.	.	G	.	.	.	G	.	.	.	.	.	.	.	.	.	.	.	.	G	.	.	.	.	.	.	.	.	.	.	.	.

Note: The column numbers (1–41) correspond to the S. No. assigned to the respective isolates in Table 1.

Supplementary Table S6: variable sites in Nad 3 gene

S. No	Samples																																									
	Vari	1	2	3	4	5	6	7	8	9	10	11	12	13	14	15	16	17	18	19	20	21	22	23	24	25	26	27	28	29	30	31	32	33	34	35	36	37	38	39	40	41
1	4	G	A	A	A	.	A	A	A	A	A	A	A	.	A	.	A	.	A	.	A	A	A	A	A	A	.	.	A	A	A	A	A	.	A	A	A	A	A	A	A	A
2	5	T	.	C	C	.	C	C	.	C	C	C	C	.	C	C	C	C	C	.	C	C	.	C	C	C	.	.	C	C	C	C	.	.	C	C	C	C	.	C	.	.
3	6	A	.	.	.	.	.	.	.	.	.	.	.	.	.	.	.	.	.	.	.	.	G	.	.	.	.	.	.	.	.	.	.	.	.	.	.	.	.	.	G	G
4	9	A	G	.	.	.	.	.	.	.	.	.	.	.	G	.	.	.	.	.	.	.	G	.	.	.	.	.	.	G	.	.	.	.	.	.	.	.	G	.	G	G
5	12	G	.	A	A	.	A	.	A	A	A	A	A	.	.	A	.	.	.	.	A	A	.	A	A	A	.	.	.	A	A	.	A	.	.	A	.	A	.	.	.	.
6	48	T	.	.	.	.	.	.	.	.	.	.	.	.	.	A	A	.	.	.	.	.	.	.	.	.	.	.	A	.	.	.	.	.	.	.	.	.	.	A	.	.
7	58	A	.	G	.	.	.	.	.	.	.	G	.	.	.	.	.	.	.	.	.	.	.	G	.	G	.	.	.	G	.	.	.	.	.	.	.	.	.	.	.	.
8	61	T	.	.	.	.	A	.	.	.	.	.	.	.	.	.	A	.	.	.	.	.	.	.	.	.	.	.	A	.	.	.	.	.	.	.	.	A	.	A	.	.
9	63	A	.	.	.	.	.	.	.	.	.	.	.	.	.	.	.	.	.	G	.	.	.	.	.	.	.	.	.	.	.	.	.	G	.	.	.	.	.	.	.	.
10	81	A	.	.	.	.	.	.	.	.	G	.	.	.	.	.	.	.	.	.	.	.	.	.	.	.	.	.	.	.	.	.	.	.	G	.	.	.	.	G	G	G
11	105	T	.	.	.	.	.	.	.	.	.	.	.	.	.	.	.	.	.	.	.	C	.	.	.	C	.	.	.	.	.	.	.	.	C	.	.	.	.	.	.	.
12	132	G	A	A	A	A	A	A	A	A	A	A	A	.	A	A	A	A	A	.	A	A	A	A	A	A	.	.	A	A	A	A	A	.	A	A	A	A	A	A	A	A
13	180	A	.	.	.	.	.	.	.	.	.	.	.	.	.	.	.	.	.	.	.	.	.	.	.	.	.	.	.	.	.	.	.	G	.	.	.	.	.	.	G	G
14	183	G	A	A	A	A	.	A	A	A	.	A	A	.	A	A	.	A	.	.	A	.	A	.	A	A	A	A	A	.	A	.	A	A	A	A	A	A	A	A	A	A
15	213	A	.	G	.	.	.	.	.	.	.	G	.	.	G	.	G	.	.	.	.	.	.	G	.	.	.	.	G	G	.	.	.	.	.	.	.	.	.	.	.	.
16	228	A	.	.	.	.	.	.	.	.	.	.	.	.	.	G	.	G	.	.	G	.	.	.	.	.	.	.	.	.	.	.	G	.	.	G	.	.	.	.	.	.
17	231	A	G	.	.	.	.	G	G	.	G	G	.	.	G	.	G	G	G	.	.	.	G	G	.	.	.	.	G	.	G	G	.	.	G	.	G	.	G	.	G	G

18	237	A	.	.	.	.	.	.	.	.	.	G	.	G	.	.	.	.	.	.	.	.	.	G	.	.	.	.	.	.	.	.	.	.	.	.	.	G	.	.	.	.
19	252	A	G	.	.	.	.	.	.	.	.	.	G	.	G	.	.	.	.	.	.	.	G	.	.	.	.	.	.	.	.	G	.	.	G	.	G	.	.	G	G	G
20	285	T	A	A	A	.	A	A	A	A	A	A	A	.	A	A	A	A	A	.	A	A	A	A	A	A	.	.	A	A	A	A	A	.	A	A	A	A	A	A	A	A
21	286	G	A	A	A	.	A	A	A	A	A	A	A	.	A	A	A	A	A	.	A	A	A	A	A	.	.	.	.	A	A	A	A	.	A	A	A	A	A	A	A	A
22	287	T	C	C	C	.	C	C	C	C	C	C	C	.	C	C	C	C	C	.	.	C	C	C	C	C	.	.	C	C	C	C	C	.	C	C	C	C	C	C	C	C
23	288	A	.	.	.	.	G	.	.	T	.	.	.	.	.	.	.	.	.	.	.	.	.	.	.	G	.	.	.	.	.	.	.	.	.	.	G	.	.	.	.	.
24	291	T	G	G	G	.	G	G	G	G	G	G	G	.	G	G	G	G	G	.	G	G	G	G	G	G	.	.	G	G	G	G	G	.	G	G	G	G	G	G	G	G
25	306	A	.	.	.	.	G	G	.	.	.	.	.	.	G	.	G	G	.	.	.	.	.	.	.	.	.	.	G	G	.	G	.	G	G	.	G	.	.	.	.	.
26	309	T	.	.	.	.	.	.	.	.	C	.	.	.	.	.	.	.	.	.	.	.	.	.	.	C	.	.	.	.	.	.	.	.	.	.	.	.	.	.	.	.
27	315	G	A	.	A	A	A	A	A	A	A	.	A	A	A	A	A	A	A	.	A	.	A	.	A	.	A	A	.	.	A	A	A	A	A	A	A	.	A	A	A	A

Note: The column numbers (1–41) correspond to the S. No. assigned to the respective isolates in Table 1.

Supplementary Table S7: Variable sites in Nad 4 gene

S. No	Samples																																									
	Vari	1	2	3	4	5	6	7	8	9	10	11	12	13	14	15	16	17	18	19	20	21	22	23	24	25	26	27	28	29	30	31	32	33	34	35	36	37	38	39	40	41
1	8	A	T	T	T	T	T	T	T	T	T	T	T	.	T	T	.	T	T	.	T	T	T	T	T	T	.	.	.	T	T	T	T	.	T	T	T	T	T	.	T	T
2	18	T	A	A	A	A	A	A	A	A	A	A	A	.	A	A	A	A	A	.	A	A	A	A	A	A	.	.	A	A	A	A	A	.	A	A	A	A	A	A	A	A
3	19	T	A	A	A	A	A	A	A	A	A	A	A	.	A	A	A	A	A	.	A	A	A	A	A	A	.	.	A	A	A	A	A	.	A	A	A	A	A	A	A	A
4	24	T	A	A	A	A	A	A	A	A	A	A	A	.	A	A	.	A	A	.	A	A	A	A	A	A	.	.	.	A	A	A	A	.	A	A	A	A	A	.	A	A
5	25	A	.	.	.	.	.	.	.	.	.	.	.	G	.	.	.	.	.	.	.	.	.	.	.	.	G	G	.	.	.	.	.	G	.	.	.	.	.	.	.	.
6	51	T	.	.	C	C	C	.	C	C	.	C	C	.	.	C	.	.	.	.	.	C	.	.	C	.	.	.	.	.	C	.	C	.	.	A	.	C	.	.	.	.
7	58	C	T	T	T	T	T	T	T	T	T	T	T	T	T	T	T	T	T	.	T	T	T	T	T	T	.	.	T	T	T	T	T	.	T	T	T	T	T	T	T	T
8	60	G	A	.	.	.	.	.	.	.	.	.	.	.	.	.	.	.	.	.	.	.	A	.	.	.	.	.	.	.	.	.	.	.	.	.	.	.	A	A	A	.
9	62	T	.	C	C	.	C	C	C	C	C	C	C	.	C	C	.	C	C	.	C	C	.	C	C	C	.	.	.	C	C	C	C	.	C	C	C	.	C	.	C	C
10	64	A	T	T	T	.	T	T	T	T	T	T	T	.	T	T	T	T	T	.	T	T	T	T	T	T	.	.	T	T	T	T	T	.	T	T	T	T	T	T	T	T
11	66	A	T	T	T	.	T	T	T	T	T	T	T	.	T	T	T	T	T	.	T	T	T	T	T	T	.	.	T	T	T	T	T	.	T	T	T	T	T	T	T	T
12	72	A	G	G	G	.	G	G	G	G	.	G	G	.	G	G	G	G	G	.	G	G	G	G	G	G	.	.	G	G	G	G	G	.	G	G	G	G	G	G	G	G
13	73	T	.	.	.	.	.	.	.	C	.	.	.	.	.	.	.	.	.	.	.	.	.	.	.	.	.	.	.	.	.	.	.	.	C	.	C	.	.	.	.	.
14	75	G	A	A	.	A	.	A	A	.	.	.	.	A	A	.	A	A	A	.	A	A	A	.	.	.	A	A	A	A	A	A	.	A	A	.	A	.	A	A	A	A

15	88	C	.	.	.	.	T	.	.	.	.	.	.	.	.	.	T	.	.	.	.	.	.	.	.	.	.	.	T	.	.	.	.	T	.	.	.	.	.	T	.	.
16	102	G	A	A	.	.	.	.	A	A	.	.	A	.	.	A	.	.	.	.	A	.	A	.	A	A	.	.	A	A	A	.	A	.	.	A	.	A	A	A	A	A
17	105	A	.	.	.	.	G	.	.	.	.	.	.	.	.	.	.	.	.	.	.	.	.	.	.	G	.	.	G	.	.	.	.	.	.	.	.	G	.	G	.	.
18	108	A	.	.	.	.	.	.	.	G	.	.	.	.	.	.	.	.	.	.	.	.	.	.	G	G	.	.	.	.	.	.	.	.	.	.	.	.	.	.	.	.
19	114	A	G	G	G	.	G	G	G	G	G	G	G	.	G	G	.	G	G	.	G	G	G	G	G	G	.	.	.	G	G	G	G	.	G	G	G	G	G	.	G	G
20	135	C	.	.	.	.	.	.	.	.	.	.	.	T	.	T	.	.	.	.	.	.	.	.	.	.	.	.	.	.	T	.	T	.	.	.	.	.	.	.	.	.
21	141	T	.	C	.	.	.	.	.	.	.	C	.	.	.	.	.	.	.	.	.	.	.	C	.	.	.	.	.	C	C	.	.	.	.	.	.	.	.	.	.	.
22	144	A	G	G	G	.	G	G	G	G	G	G	G	.	G	G	.	G	G	.	.	G	G	G	G	G	.	.	.	G	G	G	G	.	G	G	G	G	G	.	G	G
23	145	T	.	.	.	C	.	.	.	.	.	.	.	.	.	.	.	.	.	.	.	.	.	.	.	.	C	C	.	.	.	.	.	C	.	.	.	.	.	.	.	.
24	159	A	.	.	G	.	.	G	.	G	.	.	.	.	.	.	.	.	.	.	.	.	.	.	.	.	.	.	.	.	.	.	.	.	.	.	.	.	.	.	G	G
25	162	A	.	.	.	.	.	.	.	.	.	.	.	.	.	.	.	.	.	.	.	.	G	.	.	G	.	.	.	.	.	.	.	G	.	.	.	.	.	.	.	.
26	192	G	A	A	A	A	A	A	A	A	A	A	A	.	A	A	A	A	A	.	A	A	A	A	A	A	.	.	A	A	A	A	A	.	A	A	A	A	A	A	A	A
27	213	A	.	.	.	.	.	G	.	.	.	.	.	.	G	.	.	G	G	.	.	.	.	.	.	.	.	.	.	.	.	.	.	.	G	.	G	.	.	.	.	.
28	223	A	.	.	.	.	.	.	.	.	.	.	.	G	.	.	G	.	.	.	.	.	G	.	.	.	G	G	G	.	.	.	.	G	.	.	.	.	.	.	.	.
29	224	G	.	.	.	.	.	.	.	.	.	A	.	.	.	.	.	.	.	.	.	.	.	A	.	.	.	.	.	.	.	.	.	.	.	.	.	A	.	.	.	.
30	229	T	C	.	.	.	.	.	.	.	.	.	.	.	.	.	.	.	.	C	.	.	C	.	.	.	.	.	.	.	.	.	.	.	.	.	.	.	C	.	C	C
31	252	T	.	.	.	.	.	.	.	.	.	.	.	.	.	.	C	.	.	.	.	.	.	.	.	.	.	.	C	.	.	.	.	.	.	.	.	.	.	C	.	.
32	261	T	A	A	.	A	A	A	A	A	A	A	A	.	A	A	A	A	A	.	A	A	A	A	A	A	.	.	G	A	A	A	A	.	A	A	A	A	A	G	A	A
33	276	A	.	.	.	.	.	.	.	.	.	.	.	.	.	.	.	.	.	G	.	.	.	.	G	.	.	.	.	.	.	.	.	G	.	.	.	.	.	.	.	.
34	285	G	T	T	T	T	T	T	T	T	T	T	T	.	T	T	T	T	T	.	T	T	T	T	T	T	.	.	T	T	T	T	T	.	T	T	T	T	T	T	T	T
35	294	G	A	A	A	A	A	A	A	A	A	A	A	A	A	A	A	A	A	.	A	A	A	A	A	A	.	.	A	A	A	A	A	.	A	A	A	A	A	A	A	A
36	306	T	.	C	C	.	.	.	.	.	.	C	C	C	C	.	.	C	C	.	C	.	.	C	.	.	.	.	.	C	C	C	.	.	.	.	C	C	.	.	.	.
37	309	A	.	.	.	.	.	G	.	.	.	.	G	G	G	.	.	G	G	.	.	.	.	.	G	.	.	.	.	.	.	G	.	.	G	.	G	.	.	.	G	G
38	318	A	.	.	G	G	.	.	G	.	.	G	G	.	G	G	.	G	G	.	.	G	.	G	.	.	.	.	.	.	G	G	.	.	.	.	G	G	.	.	.	.
39	321	A	.	.	.	.	.	.	.	.	.	.	.	.	.	.	.	.	.	.	.	.	.	.	.	G	.	.	.	.	.	.	.	.	.	.	G	.	.	.	.	.
40	330	A	.	.	.	.	.	.	.	.	.	.	.	.	.	.	G	.	.	.	.	.	.	.	.	.	.	.	.	.	.	.	.	.	.	.	.	.	.	.	G	G
41	423	A	.	G	G	G	G	G	G	G	.	G	.	.	G	.	G	G	G	.	.	.	.	G	G	G	.	.	G	G	.	G	.	.	G	.	G	G	.	.	.	.
42	465	A	.	.	.	.	.	.	.	.	G	.	.	.	.	.	.	.	.	.	.	.	.	.	.	.	.	.	G	.	.	.	.	.	.	.	.	G	.	G	.	.
43	471	A	.	.	.	.	.	.	.	.	.	.	.	.	.	.	.	.	.	G	.	.	G	.	.	.	.	.	.	.	.	.	.	.	.	.	G	.	.	.	G	G
44	474	A	G	.	G	.	G	.	.	G	.	G	G	.	G	G	.	G	G	G	G	.	G	G	G	G	.	.	.	G	G	G	.	.	.	G	G	.	G	.	G	G

45	493	T	C	C	.	.	.	C	C	.	C	C	.	.	.	.	.	C	.	.	.	C	C	C	.	.	.	.	.	C	.	C	.	.	.	.	C	.	C	.	C	C
46	549	A	.	.	.	.	.	.	.	.	.	.	.	G	.	.	.	G	G	.	.	.	.	.	.	.	.	.	.	.	.	G	.	.	G	.	G	.	.	.	.	.
47	561	C	T	T	T	.	T	T	T	T	T	T	T	.	T	T	T	T	T	.	T	T	T	T	T	T	.	.	T	T	T	T	T	.	T	T	T	T	T	T	T	T
48	573	C	.	T	.	.	.	.	T	T	T	T	.	.	.	.	T	.	.	.	.	.	.	T	.	.	.	.	T	T	T	.	.	.	.	.	.	.	.	.	.	.
49	579	A	.	.	.	.	.	.	.	.	.	.	.	G	.	.	G	.	.	.	.	.	.	.	.	.	.	.	G	.	.	.	.	.	.	.	.	.	.	G	.	.
50	609	G	.	.	.	.	A	.	.	.	.	.	.	.	.	A	A	A	.	.	.	.	.	.	.	.	.	.	A	.	.	.	.	.	.	.	.	.	.	A	A	A
51	663	A	G	G	G	G	G	G	G	G	G	G	G	.	G	G	G	G	G	.	G	G	G	G	G	G	.	.	G	G	G	G	G	.	G	G	G	G	G	G	G	G
52	666	A	.	.	G	.	G	.	G	G	G	G	G	.	.	G	.	.	.	.	G	G	.	G	G	G	.	.	G	G	G	.	G	.	.	.	.	.	G	G	G	G
53	669	T	.	.	.	.	.	C	.	.	.	.	.	.	C	.	.	.	.	.	.	.	.	.	.	.	.	.	.	.	.	C	.	.	C	.	.	.	.	.	.	.
54	678	G	A	A	A	A	A	.	A	A	A	A	A	.	.	A	.	.	.	.	A	A	A	A	A	A	.	.	.	A	A	.	A	.	.	A	A	A	A	.	A	A
55	679	T	C	.	.	.	.	.	.	.	.	.	.	.	.	.	.	.	.	.	.	.	C	.	.	.	.	.	.	.	.	.	.	.	.	.	.	.	C	.	C	C
56	682	T	.	C	.	.	.	.	.	.	.	.	.	.	.	.	.	.	.	.	.	.	.	.	.	.	.	.	.	C	.	.	.	.	.	.	.	.	.	.	.	.
57	684	G	.	.	.	A	.	A	.	A	.	.	.	.	A	.	.	A	.	.	.	.	.	.	.	.	.	.	.	.	.	.	.	.	.	.	.	.	.	.	.	.
58	765	A	.	.	.	.	.	.	.	.	.	.	.	.	.	.	.	.	.	G	.	.	.	.	.	.	.	.	.	.	G	.	.	G	.	.	.	.	.	.	.	.
59	777	A	G	.	G	G	G	G	G	G	G	.	G	G	G	G	G	G	G	.	G	G	G	.	G	G	.	.	G	.	G	G	G	.	G	G	G	G	G	G	G	G
60	780	G	A	A	A	A	A	A	A	A	A	A	A	.	A	A	A	A	A	.	A	A	A	A	A	A	.	.	A	A	A	A	A	A	A	A	A	A	A	A	A	A
61	783	T	C	C	C	C	C	.	C	C	C	C	C	.	.	C	.	.	.	.	C	C	C	C	C	C	.	.	.	C	C	.	C	.	.	C	.	C	C	.	C	C
62	786	A	.	.	.	.	.	.	.	.	.	.	.	.	.	.	.	.	.	.	.	.	G	.	.	.	.	.	.	.	.	.	.	.	.	.	.	.	G	.	G	G
63	798	A	.	.	.	.	.	.	G	.	.	.	.	.	.	.	.	.	.	.	.	.	.	G	.	.	.	.	.	.	.	.	.	G	.	.	.	.	.	.	.	.
64	804	C	T	T	T	T	T	T	T	T	T	T	T	T	T	T	.	T	T	.	T	T	T	T	T	T	.	.	.	T	T	T	T	.	T	T	T	A	T	.	T	T
65	834	G	.	.	.	.	.	.	.	.	.	.	.	.	.	.	.	.	.	.	.	.	A	.	.	.	.	.	.	A	.	.	.	.	.	.	.	.	.	.	A	A
66	843	C	.	.	.	T	.	T	.	.	.	.	.	T	.	.	T	.	.	.	.	.	.	.	.	.	T	T	.	.	.	.	.	T	T	.	T	.	.	.	.	.
67	846	T	.	.	.	.	C	.	.	C	.	.	.	.	.	.	.	.	.	.	C	C	.	.	.	C	.	.	.	.	.	.	.	.	.	C	.	.	.	.	.	.
68	855	A	G	G	G	.	.	G	G	G	G	G	T	.	G	G	G	G	G	.	G	G	G	G	.	G	.	.	G	G	.	G	G	.	G	G	G	G	G	.	G	G
69	864	T	.	.	.	.	.	.	.	.	.	.	.	.	.	.	.	.	.	.	C	.	.	.	.	.	C	C	.	.	.	.	.	.	.	.	.	.	.	.	.	.
70	888	T	.	.	.	.	.	.	.	.	.	.	.	.	.	.	C	.	.	.	.	.	.	.	.	.	.	.	.	.	.	C	.	.	.	.	.	.	.	.	C	C
71	894	A	.	.	.	.	.	.	.	.	.	.	.	.	.	.	.	.	.	.	.	.	.	.	.	.	.	.	.	.	.	.	.	G	.	.	.	.	.	.	.	.
72	900	A	.	.	.	.	.	.	.	.	.	.	.	.	.	.	G	.	.	.	.	.	.	.	.	.	.	.	G	.	.	G	.	.	.	.	.	.	G	G	.	.
73	942	.	.	.	.	.	.	G	.	.	.	.	.	.	G	.	.	.	.	.	.	.	.	.	.	.	.	.	.	.	.	G	.	.	G	.	G	.	.	.	.	.
74	990	A	.	.	.	.	.	.	.	.	.	.	.	.	.	.	.	G	G	.	.	.	.	.	.	.	.	.	.	.	.	.	.	.	G	.	G	.	.	.	.	.

75	996	T	C	.	C	.	.	.	.	.	.	.	.	.	.	.	.	.	.	.	.	.	C	.	.	.	.	.	.	.	.	.	.	.	.	.	.	.	C	.	C	C
76	1014	G	A	.	A	A	A	A	A	A	.	A	A	A	A	.	.	A	.	.	A	A	.	A	.	A	A	A	A	A	A	.	.	.	T	A	A	.	.	.	.	.
77	1029	T	.	C	.	C	.	.	.	.	.	.	.	C	.	.	.	.	.	.	.	.	C	.	.	.	C	C	.	.	.	C	.	C	.	.	.	.	.	.	.	.
78	1032	T	.	.	.	.	.	.	A	.	.	.	.	.	.	.	.	C	C	.	.	.	.	.	.	.	.	.	.	C	.	.	.	.	.	.	.	.	.	.	.	.
79	1071	A	.	.	.	.	.	.	.	.	.	.	.	.	.	.	G	.	.	.	.	.	.	.	.	.	.	.	G	.	.	.	.	.	.	.	.	.	.	G	.	.
80	1083	T	C	.	C	.	.	C	.	C	C	.	.	.	C	C	.	.	.	.	C	C	C	.	C	C	.	.	.	.	.	C	C	.	.	C	.	C	C	C	C	C
81	1105	T	.	C	C	.	C	C	C	C	C	C	C	.	C	C	C	C	C	.	C	C	.	C	.	C	.	.	C	C	.	C	.	.	C	C	C	.	.	C	.	.
82	1111	T	C	C	.	.	.	.	.	.	C	C	.	.	C	.	.	C	C	.	.	C	C	C	.	.	.	.	.	C	C	.	.	.	C	.	.	.	C	.	.	.
83	1134	C	T	T	T	.	T	T	T	T	T	T	T	.	T	T	T	T	T	.	T	T	T	T	T	T	.	.	T	T	T	T	T	.	T	T	T	T	T	T	T	T
84	1138	G	.	.	.	.	.	.	.	.	.	.	A	.	.	.	.	A	A	.	.	.	.	.	.	.	.	.	.	.	A	.	.	.	.	.	.	.	.	.	.	.
85	1145	T	.	.	.	.	C	.	.	.	.	.	.	.	.	.	.	.	.	.	.	.	.	.	.	.	A	A	.	.	.	.	.	C	.	.	.	.	.	.	.	.
86	1149	T	C	C	C	.	C	C	C	C	C	C	C	.	C	C	C	C	C	.	C	C	C	C	.	C	.	.	.	.	C	C	C	.	C	C	C	C	C	C	C	C
87	1171	A	.	.	.	.	.	G	.	.	.	.	G	.	G	.	.	G	G	.	.	.	G	.	.	.	.	.	.	.	.	G	.	.	G	.	G	.	.	.	.	.
88	1185	G	A	A	A	.	A	A	A	A	A	A	A	.	A	A	A	A	A	.	A	A	A	A	A	A	.	.	A	A	A	A	A	.	A	A	A	A	A	A	A	A
89	1188	A	G	G	G	.	G	G	G	G	G	G	G	.	G	G	G	G	.	.	G	G	G	G	G	G	.	.	G	G	G	G	G	.	G	G	G	G	G	.	G	G
90	1209	C	T	T	T	.	T	T	T	T	T	T	T	T	T	T	T	T	T	.	T	T	T	T	T	T	.	.	T	T	T	T	T	.	T	T	T	T	T	T	T	T
91	1215	C	G	A	A	.	A	.	A	A	A	G	.	.	.	A	.	.	.	.	A	A	G	A	A	A	.	.	.	A	A	.	.	A	.	A	.	.	G	.	G	G
92	1218	A	.	.	.	.	.	.	G	G	.	.	.	G	.	.	.	.	.	.	G	.	G	.	.	.	.	.	.	.	.	.	.	.	.	G	.	.	.	.	.	.

Note: The column numbers (1–41) correspond to the S. No. assigned to the respective isolates in Table 1.

Supplementary Table S8: Variable sites in Nad 4L gene

S. No	Samples																																									
	Vari	1	2	3	4	5	6	7	8	9	10	11	12	13	14	15	16	17	18	19	20	21	22	23	24	25	26	27	28	29	30	31	32	33	34	35	36	37	38	39	40	41
1	19	A	G	G	G	.	G	G	.	G	G	G	G	.	G	G	.	G	G	.	G	G	G	G	G	G	G	G	G	G	G	G	G	.	G	G	G	G	G	G	G	G
2	24	A	G	G	G	.	G	G	G	G	G	G	G	.	G	G	.	G	G	.	G	G	G	G	G	G	.	.	G	G	G	G	G	.	G	G	G	G	G	G	G	G
3	42	G	A	A	A	.	A	A	A	A	A	A	A	.	A	A	.	A	A	.	A	A	A	A	A	A	.	.	A	A	A	A	A	.	A	A	A	A	A	A	A	A
4	66	A	G	G	G	.	G	G	G	.	G	G	G	.	G	G	G	G	G	.	G	G	G	G	G	G	.	.	G	G	G	G	.	.	G	G	.	G	G	G	G	G
5	78	G	A	A	A	.	A	A	A	A	A	A	A	A	A	A	A	A	A	.	A	A	A	A	A	A	.	.	A	A	A	A	A	.	A	A	A	A	A	A	A	A
6	96	A	G	G	G	.	G	G	G	G	G	G	G	.	G	G	G	G	G	.	G	G	G	G	G	G	.	.	G	G	G	G	G	.	G	G	G	G	G	G	G	G

7	109	A	T	T	T	.	T	.	T	T	T	T	T	.	.	T	.	.	.	.	T	T	T	T	T	T	.	.	.	T	T	.	T	.	.	T	.	T	T	.	T	T
8	112	A	G	.	.	.	.	G	.	.	.	.	.	G	G	.	G	G	G	.	.	.	G	.	.	.	.	.	G	.	.	G	.	.	G	.	G	.	G	G	G	G
9	118	G	A	A	A	.	A	A	A	A	A	A	A	.	A	A	A	A	A	.	A	A	A	A	A	A	.	.	A	A	A	A	A	.	A	A	A	A	A	A	A	A
10	121	A	G	G	G	.	G	G	G	G	G	G	G	.	G	G	G	G	G	.	G	G	G	G	G	G	.	.	G	G	G	G	G	G	G	G	G	G	G	G	G	G
11	123	A/C	.	.	.	T	.	.	.	.	.	.	.	T	.	.	.	.	.	T	.	.	.	.	.	.	T	T	.	.	.	.	.	T	.	.	.	.	.	.	.	.
12	147	A	G	G	G	.	G	G	G	G	G	G	G	G	G	G	G	G	G	.	G	G	G	G	G	G	.	.	G	G	G	G	G	.	G	G	G	G	G	G	G	G
13	156	A	G	G	G	.	G	G	.	G	G	G	G	.	G	G	G	G	G	.	G	G	G	G	G	G	.	.	G	G	G	G	G	.	G	G	G	G	G	G	G	G
14	159	T	A	A	A	.	A	A	A	A	A	A	A	.	A	A	A	A	A	.	A	A	A	A	A	A	.	.	A	A	A	A	A	.	A	A	A	A	A	A	A	A
15	160	G	A	A	A	.	A	A	A	A	A	A	A	.	A	A	A	A	A	.	A	A	A	A	A	A	.	.	A	A	A	A	A	.	A	A	A	A	A	A	A	A
16	174	G	A	A	A	.	A	.	A	A	A	A	A	.	A	A	A	A	A	.	A	A	.	A	A	A	.	.	A	A	A	A	A	.	A	A	A	A	A	A	A	A
17	177	T	.	.	.	.	.	.	.	.	.	A	.	.	.	.	.	.	.	.	.	.	.	A	.	.	.	.	.	.	A	.	.	.	.	.	.	.	.	.	.	.
18	178	A	.	.	.	.	.	.	.	.	.	.	.	.	.	.	.	.	.	.	.	.	.	.	.	G	.	.	.	.	.	.	.	.	.	.	.	.	.	.	.	.
19	180	A	G	G	G	.	G	G	G	G	G	G	G	.	G	G	G	G	G	.	G	G	G	G	G	G	.	.	G	G	G	G	G	.	G	G	G	G	G	.	G	G
20	186	A	.	.	.	.	.	.	.	.	.	.	.	G	.	.	.	.	.	.	.	.	.	.	.	.	.	.	.	.	.	.	.	.	.	G	.	.	G	.	.	.
21	195	A	.	.	.	.	.	.	G	.	.	.	.	T	.	.	T	.	.	.	.	.	.	.	.	.	.	.	.	.	.	.	.	T	.	.	.	.	.	T	.	.
22	198	T	C	C	C	.	C	C	.	C	.	C	C	.	C	C	.	.	.	.	C	C	C	C	C	C	.	.	.	.	C	C	C	.	C	C	C	C	C	.	C	C
23	201	A	.	.	.	.	G	.	.	.	.	.	.	G	.	.	.	.	.	.	.	.	G	.	.	.	.	.	.	.	.	.	.	.	.	.	.	.	.	.	.	.
24	210	C	.	T	.	.	T	T	.	T	T	.	.	.	.	.	.	.	.	.	T	.	T	.	T	.	T	T	.	T	.	.	.	T	T	T	.	T	.	T	T	T
25	216	C	T	T	T	.	T	T	T	T	T	T	T	T	T	.	T	T	T	.	T	.	T	T	T	T	.	.	T	T	T	T	.	.	T	.	T	T	.	T	T	T

Note: The column numbers (1–41) correspond to the S. No. assigned to the respective isolates in Table 1.

Supplementary Table S9: Variable site in Nad 5 gene

S. No	Samples																																									
	Vari	1	2	3	4	5	6	7	8	9	10	11	12	13	14	15	16	17	18	19	20	21	22	23	24	25	26	27	28	29	30	31	32	33	34	35	36	37	38	39	40	41

1	27	A	T	T	T	.	T	T	T	T	T	T	T	.	T	T	T	T	T	.	T	T	T	T	T	T	.	.	T	T	T	T	T	.	T	T	T	T	T	T	T	T
2	30	A	T	T	T	.	T	T	T	T	T	.	T	.	T	T	T	T	T	.	T	T	T	.	T	T	.	.	C	T	T	T	T	.	T	T	T	T	T	T	T	T
3	31	A	G	.	.	.	.	.	.	.	.	.	G	.	.	.	.	.	.	.	.	.	G	.	.	.	.	.	.	.	.	.	G	.	.	.	.	.	G	.	G	G
4	37	T	.	.	.	.	.	C	.	.	.	.	.	.	C	.	C	C	C	.	.	.	.	.	.	.	.	.	C	.	.	.	.	.	C	.	.	.	.	C	.	.
5	39	A	G	.	G	.	G	G	G	.	G	G	G	G	G	G	G	G	G	.	G	G	G	G	G	G	.	.	G	.	G	G	G	G	G	G	G	G	G	G	G	G
6	42	A	.	.	.	.	.	.	.	.	.	.	T	T	.	.	.	.	.	.	.	G	.	.	.	.	.	.	.	.	.	.	.	.	.	.	G	.	.	.	.	.
7	49	T	.	.	.	.	.	.	.	.	.	.	.	.	.	C	.	.	.	.	C	.	.	.	.	.	.	.	.	.	.	.	C	.	.	.	.	.	.	.	.	.
8	51	G	A	A	A	.	A	A	A	.	A	A	A	A	A	A	A	A	A	.	A	A	A	A	A	.	A	A	A	A	A	A	A	.	A	A	A	.	A	A	A	A
9	60	A	.	.	.	G	.	.	.	G	.	.	.	.	.	.	.	.	.	.	.	.	.	.	.	.	.	.	.	.	G	.	.	.	.	.	.	.	.	.	.	.
10	65	T	C	C	C	.	C	C	C	C	C	C	C	.	C	C	C	C	C	.	C	C	C	C	C	C	.	.	C	.	.	C	C	.	C	C	C	C	C	C	C	C
11	96	G	.	.	.	.	.	.	.	.	.	.	.	.	.	.	.	.	.	A	.	.	.	.	.	.	A	A	.	.	.	.	.	.	.	.	.	.	.	.	.	.
12	129	T	C	.	.	.	.	.	.	C	.	.	C	.	.	.	.	.	.	.	.	.	C	.	.	.	.	.	.	.	.	.	.	.	.	.	.	C	C	.	C	C
13	138	T	C	.	.	.	.	.	.	.	.	.	.	.	.	.	C	.	.	.	.	.	.	.	.	.	.	.	.	.	.	.	.	.	.	.	.	.	.	.	.	.
14	144	G	.	.	.	.	.	A	.	.	.	.	.	.	A	.	.	A	A	.	.	.	.	.	.	.	.	.	.	.	.	A	.	.	A	.	.	.	.	.	.	.
15	145	T	.	.	.	.	.	A	.	.	.	.	.	.	A	.	.	A	A	.	.	.	.	.	.	.	.	.	.	.	.	A	.	.	A	.	A	.	.	.	.	.
16	162	A	G	G	G	.	G	G	G	G	G	G	G	.	G	G	G	G	G	.	G	G	G	G	G	G	.	.	G	G	G	G	G	.	G	.	G	G	G	G	G	G
17	165	A	.	.	.	.	.	.	.	.	.	.	.	C	.	.	.	.	.	.	.	.	.	.	.	.	.	.	G	.	.	.	.	G	.	G	G	.	.	.	.	.
18	171	T	A	A	A	.	A	A	A	G	A	A	A	.	G	A	.	A	A	.	G	A	A	A	G	A	.	.	.	A	A	A	A	.	A	A	A	A	G	.	A	A
19	183	A	.	G	G	.	G	.	G	.	G	G	G	.	.	G	.	.	.	.	G	G	.	G	G	G	.	.	.	G	G	.	G	.	.	G	.	G	.	.	.	.
20	186	A	G	G	G	.	G	G	G	G	G	G	G	.	G	G	G	G	G	.	.	G	G	G	G	G	.	.	G	G	G	G	G	.	G	.	G	.	G	G	G	G
21	187	A	.	.	.	.	.	.	.	.	.	.	.	.	.	.	G	.	.	.	.	.	.	.	.	.	.	.	G	.	.	.	.	.	.	.	.	.	.	G	.	.
22	234	T	C	C	C	.	.	C	.	.	C	C	.	.	C	C	C	C	C	.	C	C	C	C	.	C	.	.	.	.	C	C	C	.	C	C	C	C	C	C	C	C
23	243	A	.	.	.	.	.	.	.	.	.	.	.	.	.	.	G	.	.	.	.	.	.	.	.	.	.	.	G	.	.	.	.	.	.	.	.	G	T	G	.	.
24	258	G	.	.	A	.	.	.	.	.	.	.	.	.	.	.	T	.	.	.	.	.	.	.	.	.	.	.	T	.	.	.	.	.	.	.	.	.	.	T	.	.
25	285	T	.	.	.	.	.	.	.	.	.	.	.	C	.	.	.	.	.	.	.	.	.	.	.	.	C	C	.	.	.	.	.	C	.	.	.	.	.	.	.	.
26	297	A	.	.	.	.	.	T	.	.	.	.	.	.	.	.	T	.	.	T	.	.	.	.	.	.	.	.	C	.	.	.	.	.	.	.	.	.	.	T	.	.
27	303	A	G	G	G	.	.	G	G	.	G	G	G	.	G	G	G	G	G	.	G	G	G	G	G	G	.	.	G	G	G	G	G	.	G	G	G	G	G	G	G	G
28	315	A	.	.	.	.	.	.	.	.	.	.	.	.	.	.	G	.	.	.	.	.	.	.	G	.	.	.	.	.	.	.	.	.	.	.	.	.	.	G	.	.
29	324	A	.	G	.	.	.	.	.	.	.	.	.	.	.	.	.	.	G	.	.	G	G	.	.	.	.	.	.	.	.	.	.	.	.	.	.	.	.	.	.	.
30	351	A	.	.	.	.	.	.	.	.	.	G	.	.	.	.	.	G	G	.	.	.	.	G	.	.	.	.	.	.	.	.	.	.	.	.	.	.	.	.	.	.

31	366	A	G	G	G	G	G	G	G	G	G	G	G	.	G	G	G	G	G	.	G	G	G	G	G	.	.	.	G	G	G	G	G	.	G	G	G	G	G	G	G	G
32	381	G	.	.	.	.	.	.	.	T	.	.	.	.	.	.	T	.	.	.	.	.	.	.	.	.	.	.	T	.	.	.	.	.	.	.	.	.	.	T	.	.
33	402	T	C	C	C	.	C	C	.	.	C	C	C	.	C	C	C	A	C	.	C	C	C	C	C	C	.	.	.	C	C	.	.	.	C	C	C	C	C	.	C	C
34	405	T	.	C	.	C	.	.	.	.	C	C	.	C	.	.	.	.	.	.	.	.	.	.	.	.	C	C	.	C	C	.	.	C	.	.	.	.	.	.	.	.
35	414	T	.	.	.	C	.	.	.	.	.	.	A	C	.	.	.	.	.	.	.	.	.	.	.	.	C	C	.	.	.	.	.	C	.	.	.	.	.	.	.	.
36	426	G	A	.	.	.	.	.	.	.	.	.	.	.	.	.	.	.	.	.	.	.	A	.	.	.	.	.	.	.	.	.	.	.	.	.	.	.	A	.	A	A
37	444	G	A	.	A	.	A	A	A	A	A	.	A	.	A	A	.	A	A	.	.	A	A	.	A	A	.	.	.	.	A	A	A	.	A	.	A	A	A	.	A	A
38	456	T	.	.	C	.	.	.	.	.	.	C	.	.	C	.	.	.	.	.	.	.	.	C	.	.	.	.	.	.	.	.	.	.	.	.	.	.	.	.	.	.
39	471	T	.	C	C	.	C	.	.	.	C	.	.	.	.	C	.	.	.	.	.	C	.	.	C	C	.	.	.	C	C	.	C	.	.	C	.	.	.	.	.	.
40	473	G	A	A	A	.	A	A	A	A	A	A	A	.	A	A	A	A	A	.	A	A	A	A	A	A	.	.	A	A	A	A	A	.	A	A	A	A	A	A	A	A
41	477	T	.	.	.	.	.	.	.	C	.	.	C	.	.	C	.	.	.	.	.	.	.	.	.	.	.	.	.	.	.	.	C	.	.	.	.	.	.	.	.	.
42	483	T	C	C	C	.	C	C	C	C	.	C	.	.	C	.	.	C	C	.	C	C	C	C	.	C	.	.	.	C	C	.	.	.	.	C	.	C	C	.	C	C
43	513	A	.	.	.	.	.	G	.	.	.	.	.	.	.	.	.	.	.	.	.	.	.	.	.	.	.	.	.	.	.	.	.	.	.	.	.	.	.	.	G	G
44	546	G	.	.	.	.	.	.	.	.	.	.	.	.	.	.	A	.	.	.	.	.	.	.	.	.	.	.	A	.	.	.	.	.	.	.	.	.	.	A	.	.
45	558	G	A	A	A	.	A	A	A	A	A	A	A	.	A	A	A	A	A	.	A	A	A	A	A	A	.	.	A	A	A	A	A	.	A	A	A	A	A	A	A	A
46	579	T	C	C	C	.	C	C	C	C	C	C	.	.	C	.	C	C	C	.	C	C	C	C	C	C	.	.	C	C	C	C	C	.	C	C	C	C	C	C	C	C
47	600	A	G	G	G	.	G	G	G	G	G	G	G	.	G	G	G	G	G	.	G	G	G	G	G	G	.	.	G	G	G	G	G	.	G	G	.	G	G	G	G	G
48	603	G	T	T	C	.	C	T	C	C	T	C	T	.	T	C	C	T	T	.	C	T	T	C	T	C	.	.	T	C	T	T	C	.	T	C	T	T	T	T	T	T
49	615	G	A	A	A	.	A	A	A	A	A	A	A	.	A	A	A	A	A	.	A	A	A	A	A	A	.	.	A	A	A	A	A	.	A	A	A	A	A	A	A	A
50	618	A	.	.	.	.	.	.	.	G	.	.	.	.	.	G	.	.	.	.	G	.	.	.	.	.	.	.	.	.	.	.	G	.	.	G	.	.	.	.	.	.
51	633	G	A	.	.	.	.	A	.	A	A	.	A	.	A	A	.	A	A	.	.	.	A	.	.	A	A	A	.	.	.	A	A	.	A	.	A	.	A	A	A	A
52	642	A	G	G	G	.	G	G	.	G	G	G	G	.	G	.	G	G	G	.	G	.	G	G	G	G	.	.	.	G	G	G	G	.	G	G	.	.	G	G	G	G
53	658	T	.	.	.	.	.	C	.	.	.	.	.	.	C	.	.	C	C	.	.	.	.	.	.	.	.	.	.	.	.	C	.	.	C	.	C	.	.	.	.	.
54	663	G	.	.	.	.	.	A	.	.	.	.	.	.	A	.	.	A	A	.	.	.	.	.	.	.	.	.	.	.	.	A	.	.	A	.	A	.	.	.	.	.
55	687	G	A	A	A	.	A	A	A	A	A	A	A	.	A	A	A	A	A	.	A	A	A	A	A	A	.	.	A	A	A	A	A	A	A	A	A	A	A	A	A	A
56	699	A	.	.	.	.	.	.	.	.	.	.	G	.	.	.	G	.	.	.	.	.	.	.	.	.	.	.	G	.	.	.	.	.	.	.	.	.	.	G	.	.
57	729	A	G	G	G	.	G	G	G	G	.	G	G	.	G	G	G	G	G	.	G	G	G	G	G	G	.	.	G	G	G	G	G	.	G	G	G	G	G	G	G	G
58	738	A	.	.	.	.	.	.	.	.	.	.	.	.	.	.	.	G	G	.	.	.	G	.	G	.	.	.	.	.	.	.	.	.	.	.	.	.	.	.	.	.
59	748	G	A	A	.	.	A	.	.	.	A	A	.	.	.	A	A	.	.	.	A	A	A	A	.	A	.	.	A	A	A	.	A	.	A	A	.	A	A	A	A	A
60	750	T	C	C	C	.	C	C	C	C	C	C	.	.	C	.	.	C	C	.	C	C	C	C	C	C	.	.	.	C	C	C	C	.	C	C	C	C	C	.	C	C

61	759	A	.	.	.	.	.	G	.	.	.	.	.	.	G	.	.	G	G	.	.	.	.	.	.	.	.	.	.	.	.	G	.	.	G	.	G	.	.	.	.	.
62	771	T	C	.	A	.	.	.	.	C	.	.	.	.	C	.	A	C	C	.	.	.	.	.	.	.	.	.	.	.	.	C	.	.	C	C	C	.	.	.	.	.
63	774	C	T	T	T	.	T	T	T	T	T	T	T	.	T	T	T	T	T	.	T	T	T	T	T	T	.	.	T	T	T	T	T	.	T	T	T	T	T	T	T	T
64	789	G	.	.	.	.	.	.	.	.	.	.	.	.	.	.	A	.	.	.	.	A	.	.	.	.	.	.	A	.	.	.	.	.	.	.	.	.	A	.	.	.
65	804	A	.	.	G	.	G	.	.	G	.	.	G	G	.	G	.	.	.	.	.	.	.	.	G	G	G	G	.	.	.	.	G	.	.	G	.	G	G	.	.	.
66	807	T	.	.	C	.	.	.	.	.	.	.	.	.	.	.	.	.	.	.	.	.	.	.	.	.	.	.	.	.	C	.	.	.	.	.	.	.	.	.	.	.
67	810	A	.	.	.	.	.	.	G	.	G	.	.	.	.	.	.	.	.	.	.	G	.	.	.	.	.	.	.	.	.	.	.	.	.	.	.	.	.	.	.	.
68	816	A	.	.	.	.	.	.	.	.	.	.	.	.	.	G	.	G	.	.	.	.	.	.	.	.	.	.	.	.	.	.	G	.	.	.	.	.	.	G	.	.
69	819	A	.	G	.	.	.	.	G	.	G	G	.	G	G	.	G	.	.	.	.	.	G	G	.	.	.	.	G	G	G	.	.	.	.	.	.	.	.	.	.	.
70	822	A	.	G	.	.	.	.	.	.	.	.	.	.	G	.	G	G	.	.	.	.	.	.	.	.	.	.	G	G	.	.	.	G	.	.	.	.	.	.	.	.
71	846	A	.	.	.	.	.	.	.	.	.	G	.	.	.	.	.	.	.	.	.	.	.	G	.	.	.	.	.	.	.	.	.	.	G	.	.	.	.	.	.	.
72	849	A	G	G	G	.	G	G	G	G	G	G	G	.	.	.	.	G	G	.	G	.	G	G	.	.	.	.	.	G	G	.	.	.	G	G	G	.	G	.	G	G
73	855	T	C	C	C	.	C	C	C	C	C	.	C	C	C	C	C	C	C	.	C	C	C	.	C	C	.	.	.	C	C	C	C	.	C	C	C	C	C	.	C	C
74	885	G	A	A	A	.	A	A	A	A	A	A	A	.	A	A	A	A	A	.	A	A	A	A	A	A	.	.	A	A	A	A	A	.	A	A	A	A	A	A	A	A
75	888	A	.	G	G	.	G	G	G	G	G	G	G	.	G	G	G	G	G	.	G	G	.	G	G	G	.	.	G	G	G	G	G	.	G	G	G	G	.	G	.	.
76	897	A	.	.	.	.	.	.	.	.	.	.	.	.	.	.	.	.	.	.	.	.	.	.	.	.	.	.	G	.	.	.	.	G	.	.	.	.	.	G	.	.
77	900	T	C	C	C	.	C	C	C	C	C	.	.	.	C	C	.	C	C	.	.	.	.	.	C	C	.	.	.	C	C	.	.	.	.	C	C	C	C	C	.	.
78	903	T	C	C	C	.	C	C	C	.	C	.	C	.	C	C	C	C	C	.	C	.	C	.	C	C	.	.	C	C	C	C	.	.	C	C	.	.	.	C	C	C
79	927	G	.	.	.	.	.	.	.	.	.	.	.	.	.	.	A	.	.	A	.	.	.	.	.	.	.	.	A	.	.	.	.	.	.	.	.	.	.	A	.	.
80	930	A	T	T	T	.	T	T	T	T	T	T	T	.	T	T	T	T	T	.	T	T	T	T	T	T	.	.	T	T	T	T	T	.	T	T	T	T	T	T	T	T
81	936	C	.	.	T	.	.	T	.	.	T	.	.	T	T	.	.	.	T	T	.	.	T	.	.	.	T	T	.	.	T	T	T	T	T	.	T	.	.	T	.	.
82	939	G	.	.	A	A	.	A	.	.	.	.	.	A	.	.	A	.	.	.	.	A	.	.	.	.	A	A	.	.	.	.	.	A	.	.	.	.	.	A	.	.
83	945	C	.	.	.	.	.	.	T	.	T	.	.	.	.	.	.	.	.	.	.	.	.	.	T	.	.	.	.	.	T	.	.	T	T	.	T	.	.	.	.	.
84	972	G	A	A	A	.	A	A	A	A	A	A	A	.	A	A	A	A	A	.	A	A	A	A	A	A	.	.	A	A	A	A	A	.	A	A	A	A	A	A	A	A
85	975	G	A	A	A	.	A	A	A	A	A	A	A	.	A	A	A	A	A	A	A	A	A	A	A	A	.	.	A	A	A	A	A	.	A	A	A	A	A	A	A	A
86	984	A	T	T	T	.	T	T	T	T	T	T	T	.	T	T	T	T	T	.	T	T	T	T	T	T	.	.	T	T	T	T	T	.	T	T	T	T	T	T	T	T
87	1005	G	.	.	.	.	.	A	.	.	.	.	.	.	A	.	.	A	A	.	.	.	.	.	.	.	.	.	.	.	.	A	.	.	A	.	A	.	.	.	.	.
88	1008	A	.	.	.	.	G	.	.	.	.	.	.	.	.	.	.	.	.	.	.	.	.	.	.	.	.	.	G	.	.	.	.	.	G	.	.	.	.	.	.	.
89	1023	A	G	G	.	.	.	.	G	.	G	G	G	.	.	G	G	G	.	.	.	G	G	G	.	G	.	.	G	G	G	.	G	.	.	G	.	G	G	G	G	G

90	1026	G	A	A	A	.	A	A	A	A	A	A	A	A	A	A	A	A	A	.	A	A	A	A	A	A	A	A	A	A	A	A	A	A	A	A	A	A	A	A	A	A
91	1029	A	G	G	G	.	.	G	.	.	.	.	G	.	.	.	.	.	.	.	.	G	G	.	.	G	G	G	G	G	G	.	G	G	G	G	.	G	G	.	G	G
92	1032	A	.	.	.	.	.	G	.	.	.	.	.	.	G	.	G	.	.	.	.	.	.	.	.	.	.	.	G	.	.	G	.	.	G	.	G	.	.	.	.	.
93	1041	A	G	G	.	.	G	G	.	G	G	G	G	.	G	G	.	.	.	.	G	G	G	.	G	G	.	.	.	G	G	.	G	.	.	G	.	.	G	.	G	G
94	1042	G	A	A	A	.	A	A	A	A	A	A	A	.	A	A	A	A	A	.	A	A	A	A	.	A	.	.	A	A	A	A	A	.	A	A	A	A	A	A	A	A
95	1053	G	A	A	A	.	A	A	A	A	A	A	A	.	A	A	A	A	A	.	A	A	A	A	A	A	.	.	.	A	A	A	A	.	A	A	A	A	A	A	A	A
96	1065	T	.	.	C	.	.	C	.	.	.	.	.	.	.	.	.	C	C	.	.	.	.	.	.	.	.	.	.	.	.	C	.	.	C	.	C	.	.	C	.	.
97	1066	A	G	G	G	.	G	G	G	G	G	G	G	.	G	G	G	G	G	.	G	G	G	G	G	G	.	.	G	G	G	G	G	.	G	G	G	.	G	G	G	G
98	1068	A	G	G	G	.	G	G	G	G	G	G	G	.	G	.	.	G	G	.	G	G	.	G	G	G	.	.	G	G	G	G	G	.	G	G	G	G	G	G	G	G
99	1069	G	.	.	A	.	.	.	A	.	.	.	.	.	.	.	.	.	A	.	.	.	.	.	.	.	.	.	.	.	.	.	.	.	.	.	.	.	.	.	.	.
100	1070	A	T	T	T	.	T	T	T	T	T	T	T	.	T	T	T	T	T	.	T	T	T	T	T	T	.	.	T	T	T	T	T	.	T	T	T	T	T	T	T	T
101	1071	A	G	.	G	.	.	.	.	.	G	.	.	.	.	G	.	G	.	.	.	G	G	.	.	.	.	.	.	.	.	.	.	.	.	.	.	.	G	G	G	G
102	1077	A	.	G	.	.	.	.	.	.	.	.	.	.	.	.	G	.	.	.	.	.	G	.	.	.	.	.	G	.	.	.	.	.	.	.	.	.	.	G	.	.
103	1086	T	.	A	.	.	.	.	.	.	.	.	.	.	.	.	.	.	.	.	.	.	.	.	.	.	.	.	.	.	.	.	.	.	.	.	.	.	.	.	.	.
104	1089	T	A	A	A	.	A	A	A	A	A	A	A	.	A	A	A	A	A	.	A	A	A	A	A	A	.	.	A	A	A	A	A	.	A	A	A	A	A	A	A	A
105	1092	A	G	G	.	.	.	.	G	.	G	G	.	.	.	G	G	G	G	.	.	G	.	G	G	G	.	.	G	G	G	G	G	.	G	.	G	.	G	.	.	.
106	1113	G	A	A	A	.	A	A	A	A	A	A	A	.	A	A	A	A	A	.	A	A	A	A	A	A	.	.	A	A	A	A	A	.	A	A	A	A	A	A	A	A
107	1140	A	.	.	.	.	.	G	.	.	.	G	.	.	G	.	.	.	.	.	.	.	.	G	.	.	.	.	.	.	.	G	.	.	G	.	G	.	.	.	G	G
108	1188	A	.	.	.	.	.	.	.	.	.	.	.	.	G	G	.	.	.	.	.	.	.	.	.	.	.	.	.	.	.	.	G	.	.	.	.	.	.	.	.	.
109	1189	A	G	G	G	.	G	G	G	.	G	G	G	.	G	G	G	G	G	.	G	G	G	G	G	G	.	.	G	G	G	G	G	.	G	G	G	G	G	G	G	G
110	1191	A	.	.	.	.	.	T	.	G	.	.	G	.	.	.	.	G	G	.	G	.	.	.	.	T	.	.	.	.	.	.	.	.	.	.	.	.	.	.	.	.
111	1192	A	.	.	.	.	.	.	.	.	.	.	.	.	.	.	.	G	G	.	.	.	.	.	.	.	.	.	.	.	.	.	.	.	.	.	.	G	.	.	.	.
112	1194	A	.	.	.	.	.	.	.	.	.	.	.	G	.	.	.	.	.	.	.	.	.	.	.	.	G	G	.	.	G	.	.	.	.	.	.	.	.	.	.	.

113	1197	A	.	.	.	.	.	.	.	.	.	.	G	G	.	.	.	.	.	.	.	.	.	.	G	.	.	.	.	.	.	.	.	.	G	G	.	G	.	G	.	.
114	1209	A	.	.	.	.	.	.	.	.	.	.	.	.	.	.	G	.	.	.	.	.	.	.	.	.	.	.	G	.	.	.	.	.	.	.	.	.	.	G	.	.
115	1215	A	.	.	.	G	.	.	.	.	.	.	.	G	.	.	.	.	.	.	.	.	.	.	.	.	G	G	.	.	.	.	.	G	.	.	.	.	.	.	.	.
116	1233	T	C	C	C	.	C	.	C	C	C	C	C	.	.	C	.	.	.	.	C	C	C	C	C	C	.	.	.	C	C	.	C	.	.	C	.	C	C	.	C	C
117	1243	T	A	A	A	.	A	.	A	A	A	A	A	.	.	A	.	.	.	.	A	A	A	A	A	A	.	.	.	A	A	.	A	.	.	A	.	A	A	.	A	A
118	1245	G	.	.	.	.	.	A	.	.	.	.	.	.	A	.	.	A	A	.	.	.	.	.	.	.	.	.	.	.	.	A	.	.	A	.	A	.	.	.	.	.
119	1278	T	.	.	.	C	.	.	.	.	.	.	.	C	.	.	.	.	.	.	.	.	.	.	.	.	C	C	.	.	.	.	.	.	.	.	C	.	.	.	.	.
120	1290	G	A	.	A	.	A	A	A	A	A	A	A	.	A	A	A	A	A	.	A	A	A	A	A	A	.	.	A	.	A	A	A	.	A	A	A	A	A	.	A	A
121	1296	A	.	.	G	.	.	.	.	.	.	.	T	.	.	.	G	.	.	.	.	.	G	.	.	.	.	.	.	.	.	.	.	.	G	G	G	.	.	G	.	.
122	1300	T	G	G	G	.	G	G	G	G	G	G	G	.	G	G	G	G	G	.	G	G	G	G	G	G	.	.	G	G	G	G	G	.	G	G	G	G	G	G	G	G
123	1308	A	G	G	G	.	G	G	G	G	G	G	G	.	G	G	G	G	G	.	G	G	G	G	G	G	.	.	G	G	G	G	G	.	G	G	G	G	G	G	G	G
124	1320	T	C	C	C	.	C	C	C	C	C	C	C	.	C	C	.	C	C	.	C	C	C	C	C	C	.	.	.	C	C	C	C	.	C	C	C	C	C	.	C	C
125	1321	A	G	G	G	.	G	G	G	G	G	G	G	.	G	G	.	G	G	.	G	G	G	G	G	G	.	.	.	G	G	G	G	.	G	G	G	G	G	G	G	G
126	1323	C	T	T	T	.	T	T	T	T	T	T	T	T	T	T	T	T	T	.	T	T	T	T	T	T	T	T	T	T	T	T	T	.	.	T	T	T	T	T	T	T
127	1324	A	G	G	G	.	G	G	G	G	G	G	G	.	G	G	G	G	G	.	G	G	G	G	G	G	.	.	G	G	G	G	G	.	G	G	G	G	G	G	G	G
128	1339	G	A	A	A	.	A	A	A	A	A	A	A	A	A	A	A	A	A	.	A	A	A	A	A	A	.	.	A	A	A	A	A	.	A	A	A	A	A	A	A	A
129	1341	T	A	A	A	.	A	A	A	A	A	A	A	.	A	A	A	A	A	.	A	A	A	A	A	A	.	.	A	A	A	A	A	.	A	A	A	A	A	A	A	A
130	1343	G	A	A	A	.	A	A	A	A	A	A	A	.	A	A	A	A	A	.	A	A	A	A	A	A	.	.	A	A	A	A	A	.	A	A	A	A	A	A	A	A
131	1360	A	.	G	.	.	.	.	G	.	G	G	.	.	.	.	.	.	.	.	.	.	.	G	.	.	.	.	.	G	G	.	.	.	.	.	.	.	.	.	.	.
132	1362	A	G	.	.	.	.	.	.	.	.	.	.	.	.	.	.	.	.	.	.	.	.	.	.	.	.	.	.	.	.	.	.	.	.	.	.	.	G	.	G	G
133	1368	T	.	A	A	.	A	.	A	A	A	A	A	.	.	A	.	.	.	.	A	A	.	A	A	A	.	.	.	A	A	.	A	.	.	A	.	A	.	A	.	.
134	1377	A	.	.	.	G	.	G	.	.	.	.	.	.	G	.	.	.	G	.	.	.	.	.	.	.	.	.	.	.	.	G	.	G	G	.	G	G	.	.	.	.
135	1395	T	C	C	C	.	C	.	.	.	C	.	.	.	.	C	C	.	.	.	C	C	C	C	A	C	.	.	C	C	C	.	C	.	.	.	.	.	A	.	C	C

136	1416	A	.	.	.	.	.	.	G	.	.	.	.	.	.	.	.	G	G	.	.	.	.	.	.	.	.	.	.	.	.	.	.	.	.	.	.	.	.	.	.	.
137	1452	A	.	.	.	.	.	.	.	.	.	.	G	.	.	.	G	.	.	G	.	.	.	.	.	.	.	.	.	.	.	.	.	.	.	.	.	.	.	.	.	.
138	1458	A	G	G	G	.	G	G	G	G	G	G	G	.	.	G	.	.	.	.	G	G	G	G	G	G	.	.	.	G	G	G	G	.	G	G	G	G	.	.	G	G
139	1476	T	.	.	.	.	.	C	.	.	.	.	.	.	C	C	.	C	C	.	.	.	.	.	.	.	.	.	.	.	.	C	.	.	C	.	C	.	.	.	.	.
140	1548	G	A	A	A	.	A	A	A	A	A	A	A	.	A	A	A	A	A	A	A	A	A	A	.	A	.	.	A	A	A	A	A	.	A	A	A	A	A	A	A	A

Note: The column numbers (1–41) correspond to the S. No. assigned to the respective isolates in Table 1.

Supplementary Table S10: Variable sites in Nad6 gene

S. No	Samples																																									
	Vari	1	2	3	4	5	6	7	8	9	10	11	12	13	14	15	16	17	18	19	20	21	22	23	24	25	26	27	28	29	30	31	32	33	34	35	36	37	38	39	40	41
1	1	A	G	G	G	.	G	G	G	G	G	G	G	.	G	G	G	G	G	.	G	G	G	G	G	G	G	G	G	G	G	G	G	.	G	G	G	G	G	G	G	G
2	6	T	A	A	A	.	A	A	A	A	A	A	A	.	A	A	A	A	A	.	A	A	A	A	A	A	.	.	A	A	A	A	A	.	A	A	A	A	A	A	A	A
3	13	A	G	G	.	.	G	G	G	G	G	G	G	.	G	G	.	G	G	.	G	G	G	G	G	G	.	.	G	G	G	G	G	.	G	G	.	G	G	.	G	G
4	15	G	A	A	A	.	A	A	A	A	A	A	A	.	A	A	A	A	A	.	A	A	A	A	A	A	.	.	A	A	A	A	A	.	.	A	A	A	A	A	.	A
5	18	A	T	T	.	.	T	T	T	T	T	T	T	.	T	T	T	T	T	.	T	T	T	T	T	T	.	.	T	T	T	T	T	G	T	T	T	T	T	T	.	.
6	27	A	.	.	G	.	.	.	.	.	.	.	.	G	.	.	.	.	.	.	.	.	.	.	.	.	G	G	G	.	.	.	.	.	.	.	.	.	.	G	.	.
7	39	G	.	.	.	.	.	.	.	.	.	.	.	.	A	.	.	.	.	A	.	.	.	.	.	.	.	.	.	.	.	.	.	.	A	.	.	.	.	.	.	.
8	48	A	.	.	.	.	.	.	.	.	.	.	.	.	.	.	.	.	.	.	.	.	G	.	.	.	G	G	.	.	.	.	.	.	.	.	.	.	.	.	.	.
9	54	G	T	T	T	.	T	T	T	T	T	T	T	.	T	T	T	T	T	.	T	T	T	T	T	T	.	.	T	T	T	T	T	.	T	T	T	T	T	T	T	T
10	57	T	.	.	C	C	.	.	.	.	.	.	.	C	.	.	.	C	.	.	.	.	C	.	.	.	C	C	.	.	.	.	.	C	.	.	.	.	.	.	.	.
11	72	G	A	A	A	.	A	A	A	A	A	A	A	.	A	A	A	A	A	.	A	A	A	A	A	A	.	.	A	A	A	A	A	.	A	A	A	A	A	A	.	.
12	81	A	.	.	.	G	.	.	.	.	.	.	.	G	.	.	.	.	.	.	.	.	.	.	.	.	G	G	.	.	.	.	.	G	.	.	.	.	.	.	.	.
13	99	A	.	G	G	.	.	G	G	G	G	G	G	.	.	G	.	.	.	.	G	G	.	G	G	.	.	.	.	G	G	G	.	.	.	G	.	G	G	.	.	.
14	102	A	G	G	G	.	G	G	G	.	G	G	G	.	G	G	G	G	G	.	G	G	G	G	G	G	.	.	G	G	G	G	G	.	G	G	G	G	G	G	G	G
15	117	G	A	A	.	.	A	A	A	A	A	A	A	.	A	A	A	.	.	.	A	A	A	A	A	A	.	.	A	A	A	A	A	.	A	A	A	.	A	A	A	A
16	123	G	.	.	A	.	A	A	.	A	.	A	.	.	A	.	.	A	A	A	A	.	.	A	A	.	.	.	.	A	A	A	.	A	.	A	A	.	.	.	.	.
17	126	A	.	G	G	.	G	G	G	G	G	G	G	.	.	G	.	G	G	.	G	G	.	G	G	G	.	.	G	G	G	G	G	G	G	.	G	.	G	G	.	.
18	132	G	A	.	.	.	A	.	A	A	.	A	.	.	A	.	A	.	.	A	A	.	A	.	.	A	A	A	A	.	.	A	A	.	.	A	A	A	A	.	.	.

19	135	A	.	.	.	.	.	.	.	.	.	.	.	.	.	G	.	G	G	.	.	.	.	.	.	.	.	.	.	G	.	.	.	.	.	.	.	.	.	.	.	.
20	165	G	.	.	.	.	.	A	.	.	A	.	.	.	A	.	.	A	A	.	.	.	.	.	.	.	.	.	.	.	.	A	.	.	A	.	A	.	.	.	.	.
21	171	G	.	.	.	A	.	A	.	.	.	.	.	A	.	.	.	A	A	.	.	.	.	.	.	.	A	A	.	.	.	A	.	A	A	.	A	.	.	.	.	.
22	186	G	.	.	A	.	.	.	.	.	.	.	.	.	.	.	.	.	.	.	.	A	.	.	.	.	.	.	.	.	.	.	.	.	.	A	.	.	.	.	.	.
23	189	A	G	G	.	.	G	G	.	G	G	.	G	G	G	G	G	G	G	.	G	G	G	.	G	G	.	.	G	G	G	G	.	G	G	G	G	G	G	G	G	G
24	207	A	.	.	.	.	.	.	.	.	.	.	.	.	.	.	.	.	.	G	.	.	.	.	.	.	.	.	.	.	.	.	.	.	.	G	.	.	.	.	G	G
25	210	A	.	.	.	.	.	.	.	G	.	.	.	.	.	.	.	G	G	.	.	.	G	.	G	.	.	.	.	.	.	.	.	.	.	.	.	.	G	.	G	G
26	228	A	.	.	G	.	.	.	.	.	.	.	.	.	.	.	.	.	.	.	.	G	.	.	.	.	G	G	.	.	.	.	.	.	.	.	.	.	.	.	.	.
27	238	T	.	.	.	A	.	.	.	.	.	.	.	.	.	.	.	.	.	.	.	.	.	.	.	.	A	A	.	.	.	.	.	.	.	.	.	.	.	.	.	.
28	246	G	A	A	.	.	A	.	A	A	A	A	A	.	A	.	A	A	A	.	.	.	A	A	A	A	.	.	A	A	A	A	A	.	A	A	A	A	A	A	A	A
29	247	G	A	A	A	.	A	A	A	A	A	A	A	.	A	A	A	A	A	.	A	A	A	A	A	A	.	.	A	A	A	A	A	.	A	A	A	A	A	A	A	A
30	252	G	A	A	A	A	A	A	A	A	A	A	A	T	A	A	A	A	A	.	A	A	A	A	A	A	A	A	A	A	A	A	A	.	A	A	A	.	A	A	A	A
31	253	G	A	A	A	A	A	A	A	A	A	A	A	A	A	A	A	A	A	.	A	A	A	A	.	A	.	.	A	A	.	A	.	A	A	A	A	A	A	A	A	A
32	256	T	.	.	.	C	.	.	.	.	.	.	.	.	.	.	C	C	.	.	.	.	.	.	.	.	.	.	.	.	.	.	.	.	.	.	.	.	C	.	.	.
33	258	A	G	G	G	.	G	G	G	G	G	G	G	.	G	G	.	.	G	.	G	G	G	G	G	G	.	.	G	G	G	G	G	.	G	G	G	G	.	G	G	G
34	264	A	G	G	G	.	G	G	G	G	G	G	G	.	G	G	.	.	.	.	G	.	G	G	G	.	.	.	G	G	G	G	G	.	G	G	.	G	.	G	.	.
35	265	A	T	T	T	.	T	T	T	T	T	T	T	.	T	T	.	.	.	.	T	.	T	T	T	.	.	.	T	T	T	T	T	.	T	T	.	T	.	T	.	.
36	267	T	.	.	.	.	.	.	G	G	G	G	G	.	.	G	.	.	.	.	G	.	.	G	.	.	.	.	.	G	G	G	G	.	.	G	.	G	.	G	.	.
37	270	G	A	.	.	A	.	.	.	.	.	.	.	.	.	.	A	A	A	.	.	A	A	.	.	A	.	.	.	.	.	.	.	A	.	.	A	.	A	.	A	A
38	271	G	A	.	.	A	.	.	.	.	.	.	.	.	.	.	A	A	A	.	.	A	A	.	.	A	.	.	.	.	.	.	.	A	.	.	A	.	A	.	A	A
39	273	G	A	.	A	A	A	.	A	A	.	A	A	A	A	A	A	A	A	.	A	A	A	.	A	A	A	A	A	A	A	A	A	A	A	A	A	.	A	A	A	A
40	280	G	.	A	A	.	A	A	A	A	A	A	A	A	A	A	.	.	.	.	A	.	.	A	A	.	.	.	A	A	A	A	A	.	A	A	.	A	.	A	.	.
41	291	A	.	T	T	.	.	T	T	T	T	T	.	.	T	T	.	.	.	.	.	.	.	T	T	.	.	.	.	T	.	T	T	.	T	.	.	T	.	.	.	.
42	295	G	.	A	A	.	.	A	A	A	A	A	.	.	A	A	.	.	.	.	.	.	.	A	A	.	.	.	.	A	.	A	A	.	A	.	.	A	.	.	.	.
43	301	G	.	.	.	.	.	.	A	A	.	A	.	A	.	A	.	.	.	.	.	.	.	A	.	.	.	.	.	A	A	.	A	.	.	.	.	A	.	.	.	.
44	302	G	A	A	A	A	A	A	.	.	A	.	A	A	.	.	A	A	A	.	A	A	A	.	A	A	A	A	A	.	A	A	A	A	A	A	A	A	A	A	A	A
45	308	T	C	C	C	C	C	C	.	.	C	.	C	.	.	.	C	C	C	.	C	C	C	.	C	C	.	.	C	.	.	C	.	C	C	C	C	C	C	C	C	C
46	315	G	.	.	.	.	.	.	T	.	.	T	.	.	T	.	.	.	.	.	.	.	.	T	.	.	T	T	.	T	.	.	.	.	.	.	T	.	.	.	.	.
47	327	A	G	G	.	.	G	G	G	G	G	G	G	.	G	G	.	.	.	.	G	.	G	G	G	G	.	.	.	G	G	G	G	.	.	G	G	G	G	.	.	.
48	338	A	G	G	G	.	G	G	G	G	G	G	G	.	G	G	.	.	.	.	G	.	G	G	G	G	G	G	.	G	G	G	G	.	.	G	G	G	G	G	.	.

49	339	T	C	C	C	.	C	C	C	.	C	C	C	.	C	C	.	.	.	.	C	.	C	C	C	C	.	.	.	C	C	C	C	.	.	C	C	C	C	.	.	.
50	340	G	A	A	.	.	A	A	A	A	A	A	A	.	.	A	.	.	.	.	A	.	A	A	A	A	.	.	.	A	A	A	.	.	.	A	A	A	A	A	.	.
51	351	G	.	.	.	A	.	.	.	.	.	.	.	A	A	.	A	A	.	.	.	.	.	.	.	.	A	A	A	.	.	.	.	A	.	.	.	.	.	.	A	A
52	352	A	G	G	G	.	G	.	G	G	G	.	G	.	.	G	.	.	G	.	G	G	G	G	.	G	.	.	.	G	G	.	G	.	.	G	G	G	G	G	.	.
53	372	G	.	.	A	.	.	.	.	.	.	.	.	A	.	.	.	.	.	.	.	.	.	.	.	.	.	.	.	.	.	.	.	A	.	.	.	.	.	.	.	.
54	382	T	C	C	.	.	C	C	.	.	C	C	C	.	C	C	.	.	C	.	C	C	C	C	C	C	.	.	.	C	C	C	C	.	C	C	.	.	C	.	C	C
55	384	A	G	G	G	.	G	G	G	G	G	G	G	.	G	G	.	G	G	.	G	G	G	G	G	G	.	.	.	G	G	G	G	.	G	G	G	G	G	G	G	G
56	393	A	G	G	G	.	G	G	G	G	G	.	G	.	G	G	.	G	G	.	G	G	G	G	G	G	.	.	.	G	G	G	G	.	G	G	G	G	G	G	G	G
57	399	T	A	A	A	.	A	A	A	A	A	A	A	.	A	A	.	A	A	.	A	A	A	A	A	A	.	.	.	A	A	A	A	.	A	A	A	A	A	.	A	A
58	411	A	G	G	G	.	G	G	.	.	G	.	G	.	G	G	.	G	G	.	G	G	G	.	G	G	.	.	.	G	.	G	G	.	G	G	G	G	.	.	G	G
59	420	A	T	T	T	.	T	T	T	T	T	T	T	.	T	T	.	T	T	.	T	T	T	T	T	T	.	.	T	T	T	T	T	.	T	T	T	T	T	T	T	T
60	429	G	A	A	A	.	A	A	A	A	A	A	A	.	A	A	.	A	A	.	A	A	A	A	A	A	.	.	A	A	A	A	A	.	A	A	A	A	A	A	A	A

Note: The column numbers (1–41) correspond to the S. No. assigned to the respective isolates in Table 1.

Supplementary Table S11: Variable site in Cytb gene

S. No	Samples																																									
	Vari	1	2	3	4	5	6	7	8	9	10	11	12	13	14	15	16	17	18	19	20	21	22	23	24	25	26	27	28	29	30	31	32	33	34	35	36	37	38	39	40	41
1	3	T	A	A	A	A	A	A	A	A	A	A	A	.	A	A	A	A	A	.	A	A	A	A	A	A	.	.	A	A	A	A	A	A	A	A	A	A	A	A	A	A
2	15	A	.	G	G	.	.	.	.	.	.	G	G	.	.	.	G	.	.	.	.	G	.	G	G	G	.	.	G	G	G	.	.	.	.	.	.	G	.	G	.	.
3	24	A	.	.	.	G	.	.	.	.	.	.	.	G	.	.	.	.	.	.	G	.	.	.	.	.	G	G	.	.	.	.	.	G	.	.	.	.	.	.	.	.
4	43	G	.	.	.	.	.	.	.	.	.	.	.	.	.	.	A	.	.	.	.	.	.	.	.	.	.	.	A	.	.	.	.	.	.	.	.	.	.	.	.	.
5	45	A	.	.	.	.	.	.	G	G	.	.	.	.	.	.	T	.	.	.	.	.	.	.	.	.	.	.	T	.	.	.	.	.	.	.	.	.	.	T	.	.
6	48	A	.	.	.	.	.	.	G	.	G	G	.	G	G	.	G	G	G	.	.	.	G	G	.	.	.	.	.	.	G	.	.	.	.	.	.	.	.	G	.	.
7	57	T	.	.	.	C	.	C	.	.	.	A	.	C	C	.	.	.	C	.	.	.	.	.	.	.	C	C	.	.	.	C	.	.	.	.	C	.	.	.	.	.
8	75	T	C	C	.	.	.	C	C	.	.	C	.	.	C	.	.	C	.	.	.	C	C	C	.	.	.	.	.	.	C	C	.	.	C	.	C	.	C	.	C	C
9	93	A	.	.	.	.	.	.	.	.	.	.	.	.	.	.	.	.	.	.	.	G	.	.	.	.	.	.	.	.	.	.	G	G	.	.	.	.	.	.	.	.
10	102	G	.	.	.	.	.	A	.	A	.	.	.	.	A	A	.	A	A	.	A	.	.	.	.	.	.	.	.	.	.	.	A	.	A	A	A	.	.	.	.	.
11	123	A	G	G	G	.	G	G	G	G	.	G	.	.	G	G	G	G	G	.	G	G	G	G	G	G	.	.	G	G	G	G	G	.	G	G	G	G	G	G	G	G
12	135	A	.	.	.	.	.	G	.	.	.	.	.	.	.	.	.	.	G	.	.	.	G	.	.	.	.	.	.	.	.	.	.	.	.	.	.	G	.	.	.	.

13	144	G	A	A	A	.	A	A	A	A	A	A	A	.	A	A	A	A	A	.	A	A	A	A	A	A	.	.	A	A	A	A	A	.	A	A	A	A	A	A	A	A
14	168	A	G	G	.	.	G	G	G	G	G	G	G	.	G	G	G	G	G	.	G	G	G	G	G	G	.	.	G	G	G	G	G	.	G	G	G	G	G	G	G	G
15	177	A	.	.	.	.	.	.	.	.	.	.	.	.	.	.	.	G	.	.	.	.	.	.	.	.	G	G	.	.	.	.	.	G	.	.	.	.	.	.	.	.
16	180	T	.	.	.	.	.	C	.	.	.	.	.	.	.	.	C	.	C	.	.	.	.	.	.	.	.	.	C	.	.	C	.	.	C	.	C	.	.	.	.	.
17	181	A	.	.	.	.	.	.	.	.	.	.	.	.	.	.	.	.	.	.	.	.	G	.	.	.	.	.	.	.	.	.	.	.	.	.	.	.	.	.	G	G
18	182	A	G	G	G	G	G	G	G	G	G	G	G	G	G	G	G	G	G	.	G	G	G	G	G	G	.	.	G	G	G	G	G	.	G	G	G	G	G	G	G	G
19	183	T	.	.	.	.	.	C	.	.	.	.	.	.	C	.	.	C	C	.	.	.	.	.	.	.	.	.	.	.	.	C	.	.	C	.	.	.	.	.	.	.
20	186	T	.	.	.	.	.	.	.	.	.	.	.	.	.	A	.	.	.	.	A	.	.	.	.	.	A	A	.	.	.	.	.	.	.	.	.	.	.	.	.	.
21	187	G	A	A	A	A	A	A	A	A	A	A	A	A	A	A	A	A	A	.	A	A	A	A	A	A	.	.	A	A	A	A	A	.	A	A	A	A	A	A	A	A
22	207	G	A	A	A	A	A	A	A	A	A	A	A	.	A	A	A	A	A	.	A	A	A	A	A	.	.	.	.	A	A	A	A	.	A	A	A	.	A	A	A	A
23	213	T	C	C	C	.	.	.	C	C	C	.	C	.	C	C	C	.	.	.	C	C	.	.	C	C	.	.	C	C	C	.	C	.	.	C	.	C	C	.	.	.
24	252	T	C	C	C	.	C	.	C	C	.	C	.	.	C	C	C	C	C	C	C	C	C	C	C	C	.	.	C	C	C	C	C	.	.	.	C	C	C	.	C	C
25	255	G	A	A	A	A	A	A	A	A	A	A	A	.	A	A	A	A	A	.	A	A	A	A	A	A	.	.	A	A	A	A	A	A	A	A	A	A	A	A	A	A
26	297	A	.	G	G	G	G	G	G	G	G	G	G	.	G	G	G	G	G	.	G	G	G	G	G	G	.	.	G	G	G	G	G	.	G	G	G	G	G	G	G	G
27	315	G	A	.	A	A	A	A	.	.	.	.	A	.	A	A	A	A	A	.	A	A	A	.	A	A	.	.	A	.	.	A	A	.	A	A	A	A	A	A	A	A
28	342	A	.	.	.	.	.	.	.	.	.	.	.	G	.	.	.	.	.	.	.	.	.	.	.	.	G	G	.	.	.	.	.	G	.	.	.	.	.	.	.	.
29	384	C	.	.	.	.	.	.	.	.	.	.	.	.	.	.	T	.	.	.	.	.	.	.	.	.	.	.	T	.	.	.	.	.	.	.	.	.	.	T	.	.
30	396	A	.	.	.	.	.	G	.	.	.	.	.	.	G	.	.	.	.	.	.	.	.	.	.	G	.	.	.	.	.	.	.	.	.	.	.	.	.	.	.	.
31	399	C	T	T	T	T	T	T	T	T	T	T	T	.	T	T	T	A	T	.	T	T	T	T	T	T	.	.	T	T	T	T	T	.	T	T	T	T	T	T	T	T
32	402	G	.	A	.	.	.	.	.	.	.	.	.	.	.	.	.	.	.	.	A	.	.	.	.	A	.	.	.	A	.	.	.	.	A	A	.	A	.	A	.	.
33	423	A	T	T	C	.	T	C	C	C	T	C	C	.	C	T	T	T	C	.	C	T	C	C	T	C	.	.	T	C	T	C	C	.	T	T	C	T	C	T	C	C
34	447	A	T	T	T	.	.	T	T	T	T	T	T	.	T	T	T	T	T	.	T	T	T	T	T	T	.	.	T	T	T	T	T	.	T	T	T	T	T	T	T	T
35	454	A	.	.	.	.	.	.	.	.	.	.	.	.	.	.	.	.	.	.	G	.	.	.	.	.	.	.	.	.	.	.	.	.	.	G	.	.	.	.	G	G

36	459	G	A	A	.	.	A	A	A	A	A	A	A	.	A	A	A	A	A	.	A	A	A	A	A	A	.	.	A	A	A	A	A	.	A	A	A	A	A	A	A	A
37	462	G	.	.	.	A	.	.	A	.	.	A	.	.	.	.	A	.	.	.	.	A	.	A	.	.	.	.	.	.	A	.	.	.	.	.	.	.	.	.	.	.
38	465	A	.	.	.	.	.	G	G	.	.	.	.	G	.	.	G	.	.	.	.	.	.	.	.	.	.	.	G	G	.	.	.	.	.	.	.	.	.	.	.	.
39	480	A	G	G	G	.	G	.	.	.	G	G	.	.	G	G	.	G	.	.	.	G	.	G	.	G	.	.	G	.	G	G	G	.	G	.	G	G	G	.	.	.
40	481	G	A	.	.	.	.	A	.	A	.	.	A	.	.	.	A	A	A	.	.	.	A	.	.	.	.	.	A	.	.	A	.	.	A	.	A	.	A	A	A	A
41	492	G	.	.	.	.	.	.	.	.	.	.	A	.	.	.	.	.	.	.	.	.	.	A	.	.	.	.	A	.	.	.	.	.	.	.	.	.	.	.	.	.
42	501	G	T	T	T	A	T	T	T	T	A	T	.	T	T	T	T	T	T	.	T	T	T	T	T	T	A	A	T	T	T	T	T	.	T	T	T	T	T	T	T	T
43	507	T	.	.	.	.	.	.	.	.	A	.	.	.	.	C	.	.	.	.	.	.	.	.	C	C	.	.	.	.	.	.	.	.	.	.	.	.	.	.	.	.
44	549	T	G	G	G	G	G	G	G	G	G	G	G	.	G	G	.	G	G	.	G	G	G	G	G	.	.	.	G	G	G	G	G	.	G	G	G	G	G	G	G	G
45	552	G	.	.	A	.	.	.	.	.	.	.	.	.	.	.	A	.	.	.	.	.	.	.	.	.	.	.	A	.	.	A	.	.	.	.	.	A	.	.	.	.
46	561	A	.	.	.	.	.	G	.	.	.	.	G	.	G	.	.	G	G	.	.	.	.	.	.	G	.	.	.	.	G	G	.	.	G	.	G	G	.	G	.	.
47	564	A	.	.	.	.	.	.	.	.	.	.	G	.	.	.	.	.	.	.	.	.	.	.	.	.	.	.	.	.	G	.	.	.	.	.	.	.	.	.	.	.
48	567	A	.	.	.	.	.	.	.	.	.	.	.	G	G	.	G	.	.	.	.	.	.	.	.	.	.	.	G	.	.	.	.	G	.	G	G	.	.	.	.	.
49	594	C	.	.	.	.	.	.	T	.	.	.	.	.	.	.	.	T	T	.	.	.	.	.	.	.	.	.	.	.	.	.	.	.	.	.	.	.	.	T	.	.
50	597	T	C	C	C	.	C	C	C	C	.	C	C	.	C	C	C	.	C	.	C	C	C	C	C	C	.	.	C	C	C	C	C	.	C	C	C	C	C	C	C	C
51	600	A	.	.	.	T	.	.	.	.	.	.	.	T	.	.	T	.	.	T	.	.	.	.	.	.	T	T	T	.	.	.	.	T	.	.	.	.	.	T	.	.
52	603	G	.	.	A	.	A	A	.	.	.	.	.	.	A	.	A	A	A	.	A	A	.	.	.	.	.	.	A	.	.	A	.	.	A	A	A	A	.	.	.	.
53	606	A	.	.	.	.	.	.	.	.	.	.	.	.	.	.	.	.	.	.	.	.	G	.	.	.	.	.	.	.	.	.	.	.	.	.	.	.	.	.	G	G
54	613	A	.	.	.	.	.	.	.	.	.	.	G	G	.	.	.	.	.	.	.	.	.	.	.	.	.	.	.	.	.	.	.	.	.	.	.	.	.	.	G	G
55	616	G	.	.	.	.	.	.	.	.	.	.	.	.	.	.	.	.	.	.	.	.	A	.	.	.	.	.	.	.	.	.	.	.	.	.	.	.	.	.	A	A
56	627	C	.	.	.	T	.	.	.	.	.	.	.	T	T	.	.	.	.	.	.	.	.	.	.	.	T	T	.	.	.	.	T	T	.	.	.	.	.	.	.	.
57	630	G	.	.	.	A	.	.	.	.	.	.	A	.	.	.	.	.	.	.	.	.	.	.	.	.	.	.	.	.	.	.	.	.	A	.	.	.	.	.	.	.
58	636	T	.	.	C	.	C	.	.	C	.	.	C	.	.	C	.	.	.	.	C	C	.	.	C	C	.	.	.	.	.	.	C	.	.	C	.	.	.	C	.	.

59	645	A	G	G	G	.	G	G	.	.	G	G	G	.	G	.	G	G	G	.	.	.	G	G	G	G	.	.	.	G	G	G	.	.	G	.	G	.	G	.	G	G
60	646	A	.	.	.	.	.	T	.	.	.	.	.	.	T	.	.	T	T	.	.	.	.	.	.	.	.	.	.	.	.	T	.	.	T	.	T	.	.	.	.	.
61	647	A	.	.	.	.	.	G	.	.	.	.	.	G	G	.	.	G	G	.	.	.	.	.	.	.	.	.	.	.	G	G	.	G	G	.	G	.	.	.	.	.
62	654	T	A	A	A	.	A	A	A	A	A	A	A	.	A	A	.	A	A	.	A	G	A	A	A	A	.	.	.	A	A	A	A	.	A	A	A	A	A	.	A	A
63	657	G	T	C	C	.	T	T	T	T	T	T	T	.	T	C	T	T	T	.	T	T	T	T	T	C	.	.	T	T	T	T	T	.	T	T	T	C	T	T	T	T
64	660	G	A	A	A	.	A	A	A	A	A	A	A	.	A	A	A	A	A	.	.	A	A	A	A	A	.	.	A	A	A	A	A	.	A	.	A	A	A	.	A	A
65	700	G	A	A	A	A	A	.	A	A	A	A	A	.	.	A	.	.	.	.	A	A	A	A	A	.	.	.	.	A	A	.	A	.	.	A	.	.	A	.	A	A
66	708	T	A	A	A	A	A	A	A	A	A	A	A	A	A	A	A	A	A	.	A	A	A	A	A	A	.	.	A	A	A	A	A	.	A	A	A	A	A	A	A	A
67	711	A	.	.	.	.	.	.	.	.	.	.	.	.	.	.	.	.	.	.	.	.	.	.	.	.	.	.	.	.	.	.	.	.	.	.	.	.	.	.	G	G
68	724	T	A	A	A	A	A	A	A	A	A	A	A	.	A	A	A	A	A	.	A	A	A	A	A	A	.	.	A	A	A	A	A	.	A	A	A	A	A	A	A	A
69	734	T	.	.	.	.	.	.	.	.	.	.	.	.	.	C	.	.	.	.	C	.	.	.	.	.	.	.	.	.	.	.	C	.	.	.	.	.	.	.	.	.
70	738	A	G	G	G	G	G	G	G	G	G	G	.	.	G	G	G	G	G	.	G	G	G	G	G	G	.	.	G	G	G	G	G	.	G	G	G	.	G	G	G	G
71	747	G	.	A	A	A	A	.	A	A	A	A	A	.	.	A	A	.	.	.	A	A	.	A	A	A	.	.	A	A	A	A	A	.	.	A	.	A	.	A	.	.
72	762	A	G	G	G	G	G	G	G	G	G	G	G	.	G	G	G	.	.	.	G	.	G	G	G	G	.	.	G	G	G	G	G	.	G	G	G	G	G	G	G	G
73	765	T	.	.	.	.	.	.	.	.	.	.	.	.	.	.	.	.	.	.	.	.	.	.	.	.	.	.	.	.	.	C	.	.	.	.	.	.	C	.	.	.
74	783	A	T	T	T	T	T	T	T	T	T	T	T	.	T	T	T	T	T	.	T	T	T	T	T	T	G	G	T	T	.	T	T	.	T	T	T	T	T	T	T	T
75	786	T	G	.	.	.	.	.	.	.	.	.	A	.	.	.	.	.	.	.	.	.	A	.	.	A	.	.	.	.	.	.	.	.	.	G	.	.	G	.	G	G
76	795	G	.	.	.	.	.	.	.	A	.	.	.	.	.	.	.	.	.	.	.	.	.	.	.	.	.	.	.	.	.	.	.	A	.	.	.	.	.	.	.	.
77	798	A	G	.	G	G	G	G	G	G	G	G	G	.	G	G	.	G	G	.	G	.	G	G	.	G	.	.	.	G	G	G	G	.	G	G	G	G	G	.	G	G
78	822	T	C	C	C	.	C	C	C	C	C	C	C	.	C	C	C	C	C	.	C	C	C	C	C	C	.	.	C	C	C	C	.	.	C	C	C	C	C	C	.	.
79	840	T	.	.	.	.	.	.	.	.	.	.	.	.	.	.	.	.	.	C	.	.	.	.	.	.	.	.	.	.	.	.	.	C	.	.	.	.	.	C	.	.
80	843	C	.	.	T	.	.	.	.	.	.	.	.	.	.	.	T	.	.	T	.	.	.	.	.	.	.	.	T	.	.	T	T	.	.	.	T	.	.	T	.	.
81	862	A	G	G	G	G	G	G	G	G	G	G	G	.	G	G	G	G	G	.	G	G	G	G	G	G	.	.	G	G	G	G	G	.	G	G	G	G	G	G	G	G

82	864	T	.	.	.	.	.	.	.	.	.	.	.	.	.	.	C	.	.	.	.	.	.	.	.	.	.	.	C	.	.	.	.	.	.	.	.	.	.	C	.	.
83	910	A	.	.	.	.	G	.	.	.	.	.	.	.	.	.	.	.	.	.	.	.	.	.	.	G	.	.	.	.	.	.	G	.	.	.	.	.	.	.	.	.
84	937	A	.	T	T	.	T	.	T	T	T	T	T	.	.	T	.	.	.	.	T	T	.	T	T	T	.	.	.	T	T	.	T	.	.	T	.	T	.	.	.	.
85	971	T	C	.	C	.	C	.	.	C	C	C	C	.	.	C	.	.	.	.	C	.	C	C	C	C	.	.	.	C	C	.	C	.	.	C	.	C	C	.	C	C
86	979	G	.	.	.	.	.	.	.	.	.	.	.	.	.	.	A	.	.	.	.	.	.	.	.	.	.	.	A	.	.	.	.	.	.	.	.	.	A	A	.	.
87	982	T	.	.	.	.	.	.	.	.	.	.	.	C	.	.	.	.	.	C	.	.	C	.	.	.	.	.	.	.	.	.	.	.	.	.	.	.	.	.	.	.
88	990	G	A	A	A	.	A	A	A	A	A	A	A	.	A	A	A	A	A	.	A	A	A	A	A	A	.	.	A	A	A	A	A	.	A	A	A	A	A	A	A	A
89	1008	T	.	.	.	.	.	A	.	.	.	.	.	.	A	.	A	A	A	.	.	.	.	.	.	.	.	.	A	.	.	A	.	.	A	.	A	.	.	A	.	.
90	1024	T	.	.	.	.	.	.	.	.	.	.	.	.	.	.	.	.	.	.	A	.	.	.	.	A	.	.	A	.	.	.	.	.	.	.	.	.	.	.	.	.
91	1026	A	.	G	.	.	G	.	.	.	.	G	.	.	.	.	.	G	.	.	G	G	.	G	G	.	.	.	.	G	G	.	.	.	.	.	.	.	.	.	.	.
92	1032	A	.	.	.	.	.	.	.	.	.	.	.	.	.	.	G	.	G	.	.	.	G	.	.	.	.	.	G	.	.	.	.	.	.	.	.	.	.	.	G	G
93	1035	T	A	A	G	.	A	A	A	A	A	G	A	.	A	A	A	A	A	.	A	A	A	A	A	A	.	.	A	A	A	A	A	.	A	A	A	A	A	A	A	A
94	1050	A	.	.	.	.	.	.	.	.	.	.	.	.	.	.	G	.	.	.	G	.	.	.	.	.	.	.	G	.	.	.	.	.	.	.	.	.	.	.	.	.
95	1078	A	.	.	.	.	.	.	.	.	.	.	.	.	G	.	.	.	.	.	.	.	.	.	.	.	.	.	.	.	.	.	.	.	.	.	.	.	.	.	.	.
96	1080	A	.	G	G	.	G	.	G	.	G	G	G	.	G	G	.	.	.	.	.	G	.	G	G	G	.	.	.	G	.	.	G	.	.	G	G	G	.	.	.	.

Note: The column numbers (1–41) correspond to the S. No. assigned to the respective isolates in Table 1.

Supplementary Table S12 : Variable sites in Atp 6

S. No	Samples																																								
	Vari	1	2	3	4	5	6	7	8	9	10	11	12	13	14	15	16	17	18	19	20	21	22	23	24	25	26	27	28	29	30	31	32	33	34	35	36	37	38	39	40	41
1	27	G	T	T	T	T	T	T	T	T	T	T	T	.	T	T	T	T	T	.	T	T	T	T	T	T	.	.	T	T	T	T	T	.	T	T	T	T	T	T	T	T
2	30	T	.	C	.	.	.	.	C	C	C	C	.	.	.	C	.	.	.	.	C	.	.	C	C	.	.	.	.	C	.	.	.	.	.	C	.	.	C	.	.	.
3	33	A	G	G	G	G	G	G	G	G	G	G	G	.	G	G	G	G	G	.	G	G	G	G	G	G	.	.	G	G	G	G	G	.	G	G	G	G	G	G	G	G
4	48	A	G	G	G	G	G	G	G	G	G	G	G	.	G	G	.	G	G	.	G	G	G	G	G	G	.	.	.	G	G	G	G	.	G	G	.	G	G	.	G	G
5	60	A	G	G	G	G	G	G	G	G	G	G	G	.	G	G	G	G	G	.	G	.	.	G	G	G	.	.	G	.	G	G	G	.	G	G	G	G	G	G	G	G

6	67	A	G	G	G	G	G	G	G	G	G	G	G	G	G	G	G	.	G	.	G	G	G	G	.	G	G	G	G	G	G	G	G	.	G	.	G	G	G	.	G	G
7	72	T	.	.	.	.	.	.	.	.	.	.	.	.	.	.	.	C	C	.	.	.	.	.	.	.	.	.	.	.	.	.	.	.	.	.	C	.	.	.	.	.
8	75	G	A	A	A	A	.	A	A	A	A	A	A	A	A	A	A	A	A	.	A	A	A	A	A	A	A	A	A	A	A	A	A	.	A	A	A	A	A	A	A	A
9	91	G	A	A	A	A	A	A	A	A	A	A	A	.	A	A	A	A	A	.	A	A	A	A	A	A	.	.	A	A	A	A	A	.	A	A	A	A	A	A	A	A
10	129	T	.	.	.	.	.	.	.	.	.	.	.	.	.	.	.	.	.	.	.	.	C	.	.	.	.	.	.	.	.	.	.	.	.	.	.	.	C	C	.	.
11	133	T	A	A	A	A	A	A	A	A	A	A	A	.	A	A	A	A	A	.	A	A	A	A	A	.	.	.	A	A	A	A	A	.	A	A	A	A	A	A	A	A
12	138	A	.	G	.	.	G	.	.	.	.	.	G	.	.	.	G	G	G	.	.	G	.	.	.	.	.	.	G	G	.	.	.	.	.	.	.	.	.	.	.	.
13	148	T	.	.	.	.	.	.	.	.	.	.	A	.	.	.	.	.	.	.	.	.	.	.	.	.	.	.	C	.	.	.	.	.	.	.	.	.	.	.	C	C
14	150	A	.	G	G	.	G	.	G	G	G	G	G	.	G	G	G	.	G	.	G	G	G	G	.	G	.	.	G	.	G	G	G	G	.	G	G	.	G	.	G	G
15	153	A	.	.	.	.	.	G	.	.	.	G	.	.	G	.	.	G	G	.	.	.	.	G	.	.	.	.	.	.	.	G	.	.	G	.	G	.	.	.	.	.
16	166	G	T	T	T	T	T	T	T	T	T	T	T	.	T	T	T	T	T	.	T	T	T	T	T	T	.	.	T	T	T	T	T	.	T	T	T	T	T	T	T	T
17	222	G	A	A	A	A	A	A	A	A	A	A	A	.	A	A	A	A	A	.	A	A	A	A	A	A	.	.	A	A	A	A	A	.	A	A	A	A	A	A	A	A
18	228	T	.	C	C	C	.	.	.	C	C	.	C	.	.	C	C	.	.	.	C	C	.	C	.	C	.	.	.	C	.	.	C	.	.	C	.	.	C	.	C	C
19	234	T	.	.	.	.	.	C	.	.	.	.	.	.	C	.	.	C	C	.	.	.	.	.	.	.	.	.	C	.	.	C	.	C	.	C	C	.	.	.	.	.
20	240	A	G	G	.	G	.	G	G	.	G	G	G	.	G	G	G	.	G	.	.	.	G	G	G	G	.	.	G	G	.	G	.	.	G	G	G	G	G	.	G	G
21	243	G	A	A	A	A	.	A	A	A	A	A	A	.	.	A	.	A	.	A	A	.	.	A	A	A	A	A	.	A	A	.	A	.	A	A	A	A	A	A	.	.
22	246	A	G	G	G	.	G	G	G	G	.	G	G	.	G	G	G	G	G	.	G	G	G	G	G	G	.	.	G	G	G	G	G	.	G	G	G	G	G	G	G	G
23	261	C	.	.	.	.	.	.	.	.	.	.	.	.	.	.	.	.	.	.	.	.	T	.	.	T	.	.	.	.	.	.	.	.	.	T	.	.	.	.	.	.
24	264	A	.	.	.	.	.	.	.	.	.	.	.	.	.	.	.	T	T	.	.	.	.	.	.	.	G	G	.	.	.	.	.	.	.	G	.	.	.	.	T	T
25	276	A	.	.	.	.	.	G	.	.	.	.	.	.	G	.	.	G	G	.	.	.	.	.	.	.	.	.	.	.	.	G	.	.	G	.	G	.	.	.	.	.
26	321	T	.	.	.	.	.	.	.	.	.	.	.	.	.	.	.	.	.	.	C	.	.	.	.	.	C	C	.	.	.	.	.	.	.	.	.	.	.	.	.	.
27	327	A	.	.	.	.	.	G	.	.	.	G	.	.	.	.	.	G	G	.	.	.	G	.	.	.	.	.	.	.	.	G	.	.	G	.	.	.	.	.	.	.
28	333	A	.	.	.	.	.	.	.	.	.	.	.	.	.	.	.	.	.	.	.	.	.	.	.	.	G	G	.	.	.	.	.	.	.	.	.	.	.	.	.	.
29	335	G	A	A	A	.	A	A	A	A	A	A	A	.	A	A	A	A	A	.	A	A	A	A	A	A	.	.	A	A	A	A	A	.	A	A	A	.	A	A	A	A
30	336	T	C	C	C	.	C	C	C	C	C	C	C	.	C	C	C	C	C	.	C	C	C	C	C	C	.	.	C	C	C	C	C	.	C	C	C	.	C	C	C	C
31	339	A	G	.	.	.	.	.	.	G	.	.	.	.	.	.	.	.	.	.	.	.	.	.	.	.	.	.	.	.	.	.	G	.	G	.	G	.	.	.	.	.
32	344	A	.	G	G	.	G	G	.	.	G	G	G	.	G	G	.	G	G	.	G	G	G	G	G	G	G	G	G	G	G	G	G	.	G	G	G	G	G	G	G	G
33	345	A	.	.	.	.	.	.	.	.	.	.	.	.	.	.	G	.	.	.	.	.	.	.	.	.	.	.	G	.	.	.	.	.	.	.	.	.	.	G	.	.
34	351	A	.	.	T	.	.	.	.	.	T	.	.	.	.	.	.	.	.	.	.	.	.	.	T	.	.	.	.	.	.	.	.	.	.	.	.	T	T	.	.	.
35	369	G	A	A	A	.	.	A	.	A	A	A	A	.	A	A	A	.	A	.	A	A	A	A	A	A	.	.	A	A	A	A	A	.	A	A	A	.	A	A	A	A

36	372	G	A	A	A	.	A	A	A	A	A	A	A	.	A	A	A	A	A	.	A	A	A	A	A	A	.	.	A	A	A	A	A	.	A	A	A	A	A	.	A	A
37	381	A	G	G	G	.	.	G	G	G	G	G	.	.	G	G	G	G	G	.	G	G	G	G	G	G	.	.	.	G	G	G	G	.	G	G	.	G	G	G	G	G
38	390	A	.	.	.	.	.	.	.	.	.	.	.	.	.	.	.	.	.	.	G	.	G	.	.	.	.	.	.	.	.	G	.	G	.	.	.	.	.	.	.	.
39	399	C	T	.	T	.	.	.	T	.	.	.	.	.	.	.	.	.	.	.	.	.	.	.	.	.	.	.	.	.	.	.	.	T	.	.	T	T	.	.	.	.
40	405	G	A	A	A	A	A	A	A	A	A	A	A	.	A	A	.	A	A	.	A	A	.	A	A	A	.	.	.	A	A	A	A	.	A	A	A	A	A	.	.	.
41	411	G	A	A	A	A	A	A	A	A	A	A	A	.	A	A	A	A	A	.	A	A	A	A	A	A	.	.	A	A	A	A	A	.	A	A	A	A	A	A	A	A
42	417	A	.	.	.	.	.	.	.	G	.	.	.	.	.	.	G	.	.	.	G	.	.	.	.	.	.	.	G	.	.	.	.	.	.	.	.	.	G	G	.	.
43	429	T	.	A	A	A	A	C	A	A	A	A	A	.	C	A	A	C	C	.	A	A	.	A	A	A	.	.	A	A	A	C	A	A	C	A	C	A	A	C	A	A
44	441	T	.	C	.	.	.	.	.	.	.	.	.	.	.	.	C	.	.	C	.	.	.	.	.	.	.	.	.	C	.	.	.	.	.	.	.	.	.	.	.	.
45	448	T	.	C	.	.	.	.	.	.	.	C	C	.	.	.	.	.	.	.	.	.	.	C	C	C	.	.	.	C	.	.	.	.	.	.	.	C	.	.	C	C
46	453	T	.	.	.	.	.	.	.	.	.	.	.	.	.	.	.	.	.	.	.	.	.	.	.	.	.	.	.	.	.	.	.	.	.	.	.	.	.	C	.	.
47	462	A	.	G	.	.	.	.	G	.	G	G	.	.	.	G	.	.	.	.	.	.	.	G	.	.	.	.	.	G	G	.	G	.	.	.	.	.	.	.	.	.
48	465	A	.	.	G	.	.	.	.	.	.	.	.	.	G	.	G	.	.	.	.	.	.	.	.	.	G	G	.	.	.	.	.	G	.	G	.	.	G	.	.	.
49	471	A	.	.	.	.	.	.	.	.	.	.	.	.	.	.	.	G	G	.	.	.	G	.	.	.	.	.	.	.	.	.	.	.	.	.	.	.	.	.	.	.
50	492	G	A	A	A	A	A	A	A	.	A	A	A	.	A	A	A	A	A	.	A	A	A	A	A	A	.	.	A	A	A	A	A	.	A	A	A	A	T	A	A	A
51	495	A	.	.	.	.	.	G	.	.	.	.	.	.	.	.	.	G	.	.	.	G	.	.	.	.	.	.	.	.	.	.	.	.	.	.	.	.	.	.	.	.
52	507	A	.	.	.	.	.	.	.	.	.	.	.	.	.	G	.	.	.	.	.	.	G	.	.	.	.	.	.	.	.	.	.	.	.	.	.	.	.	.	G	G
53	510	A	G	G	G	G	G	G	G	G	G	G	G	.	G	G	G	G	G	.	G	G	G	G	G	G	.	.	G	G	.	.	G	.	G	G	G	G	G	G	G	G
54	522	T	.	C	.	C	C	C	.	C	C	C	C	.	.	C	.	.	.	.	C	.	C	C	.	C	.	.	.	C	C	C	.	.	C	C	C	C	.	.	C	C
55	543	G	.	.	.	.	.	.	.	.	.	.	.	.	A	.	.	A	A	.	.	.	.	.	.	.	.	.	.	.	.	A	.	.	A	.	A	.	.	.	.	.

Note: The column numbers (1–41) correspond to the S. No. assigned to the respective isolates in Table 1.

Supplementary Table S13 : Percent identity (%) of the present study *H. contortus* mt genome isolates with the published genome of *H. contortus*

S. No	Sample ID	CP035799.1		NC_010383.2		LS 997568.1		CP149429.1	
		% identity	Query coverage	% identity	Query coverage	% identity	Query coverage	% identity	Query coverage
**1**	Pk 3_04	98.96%	100.00%	96.19%	100.00%	96.10%	100.00%	96.21%	99.00%
**2**	Pk 3_05	95.32%	100.00%	92.90%	100.00%	92.76%	100.00%	92.97%	99.00%
**3**	Pk 3_06	95.25%	100.00%	92.79%	100.00%	92.65%	100.00%	92.88%	99.00%
**4**	Pk 4_04	95.30%	100.00%	92.83%	100.00%	92.74%	100.00%	92.92%	99.00%
**5**	Pk 4_05	98.44%	100.00%	95.12%	100.00%	95.10%	100.00%	95.00%	99.00%
**6**	Pk 4_06	95.40%	100.00%	92.83%	100.00%	92.72%	100.00%	93.02%	99.00%
**7**	Pk 5_05	95.09%	100.00%	92.54%	100.00%	92.39%	100.00%	92.62%	99.00%
**8**	Pk 5_06	95.38%	100.00%	92.91%	100.00%	92.81%	100.00%	92.93%	99.00%
**9**	Pk 8_03	95.26%	100.00%	92.66%	100.00%	92.59%	100.00%	92.72%	99.00%
**10**	Pk 11_01	95.26%	100.00%	92.92%	100.00%	92.73%	100.00%	92.91%	99.00%
**11**	Pk 11_02	95.23%	100.00%	92.75%	100.00%	92.64%	100.00%	92.74%	99.00%
**12**	Pk 12_02	95.37%	100.00%	92.75%	100.00%	92.69%	100.00%	92.92%	99.00%
**13**	Pk 13_03	98.32%	100.00%	95.58%	100.00%	95.57%	100.00%	95.61%	99.00%
**14**	Pk 14_01	95.16%	100.00%	92.65%	100.00%	92.49%	100.00%	92.74%	99.00%
**15**	Pk 15_01	95.05%	100.00%	92.64%	100.00%	92.50%	100.00%	92.74%	99.00%
**16**	Pk 15_02	95.59%	100.00%	93.17%	100.00%	93.23%	100.00%	93.30%	99.00%
**17**	Pk 15_03	95.27%	100.00%	92.68%	100.00%	92.59%	100.00%	92.82%	99.00%
**18**	Pk 15_04	95.16%	100.00%	92.52%	100.00%	92.39%	100.00%	92.71%	99.00%
**19**	Pk 15_05	98.77%	100.00%	96.12%	100.00%	96.07%	100.00%	96.11%	99.00%
**20**	Pk 16_02	95.25%	100.00%	92.62%	100.00%	92.54%	100.00%	92.73%	99.00%
**21**	Pk 17_01	95.35%	100.00%	92.87%	100.00%	92.69%	100.00%	92.93%	99.00%
**22**	Pk 17_03	95.26%	100.00%	92.73%	100.00%	92.62%	100.00%	92.87%	99.00%
**23**	Pk 17_04	95.34%	100.00%	92.89%	100.00%	92.70%	100.00%	92.84%	99.00%
**24**	Pk 17_05	95.51%	100.00%	93.03%	100.00%	92.97%	100.00%	93.16%	99.00%
**25**	Pk 18_01	95.27%	100.00%	92.77%	100.00%	92.56%	100.00%	92.79%	99.00%
**26**	Pk 18_02	98.46%	100.00%	95.93%	100.00%	96.86%	100.00%	95.81%	99.00%
**27**	Pk 18_03	98.46%	100.00%	95.93%	100.00%	96.86%	100.00%	95.81%	99.00%
**28**	Pk 18_04	95.42%	100.00%	92.95%	100.00%	92.90%	100.00%	93.10%	99.00%
**29**	Pk 18_05	95.30%	100.00%	92.82%	100.00%	92.70%	100.00%	92.90%	99.00%
**30**	Pk 19_05	95.19%	100.00%	92.66%	100.00%	92.49%	100.00%	92.78%	99.00%
**31**	Pk 23_03	95.12%	100.00%	92.56%	100.00%	92.38%	100.00%	92.80%	99.00%
**32**	Pk 24_02	95.19%	100.00%	92.52%	100.00%	92.49%	100.00%	92.65%	99.00%
**33**	Pk 32_03	98.31%	100.00%	95.89%	100.00%	95.63%	100.00%	97.33%	99.00%
**34**	Pk 34_03	95.31%	100.00%	92.85%	100.00%	92.62%	100.00%	92.86%	99.00%
**35**	Pk 41_02	95.25%	100.00%	92.65%	100.00%	92.60%	100.00%	92.80%	99.00%
**36**	Pk 41_03	95.36%	100.00%	92.90%	100.00%	92.79%	100.00%	92.97%	99.00%
**37**	Pk 45_01	95.31%	100.00%	92.72%	100.00%	92.58%	100.00%	92.67%	99.00%
**38**	Pk 47_01	95.27%	100.00%	92.69%	100.00%	92.60%	100.00%	92.84%	99.00%
**39**	Pk 47_03	95.41%	100.00%	93.09%	100.00%	92.93%	100.00%	93.12%	99.00%
**40**	Pk 48_01	95.24%	100.00%	92.74%	100.00%	92.55%	100.00%	92.81%	99.00%
**41**	Pk 48_02	95.19%	100.00%	92.74%	100.00%	92.55%	100.00%	92.81%	99.00%

Data retrieved on 13/09/ 2025 from NCBI GenBank

Supplementary Table S14: Comparative analysis of H. contortus with the reference mt genome sequences of nematodes retrieved from GenBank

	Species	Phylum	Order	Super Family	Family	Accession No	Mt genome length
1	*H. contortus*	Nematoda	Rhabditida	Strongyloidea	Trichostrongylidae	CP035799.1	14001 bp
2	*H.contortus*	Nematoda	Rhabditida	Strongyloidea	Trichostrongylidae	NC_010383.2	14055 bp
3	*H. contortus*	Nematoda	Rhabditida	Strongyloidea	Trichostrongylidae	LS997568.1	14018 bp

4	*H. contortus*	Nematoda	Rhabditida	Strongyloidea	Trichostrongylidae	CP149429.1	14107 bp
5	*H. placei*	Nematoda	Rhabditida	Strongyloidea	Trichostrongylidae	AP017687	14251 bp
6	*H. placei*	Nematoda	Rhabditida	Strongyloidea	Trichostrongylidae	NC_029736	13664 bp
7	*C. oncophora*	Nematoda	Rhabditida	Strongyloidea	Trichostrongylidae	OZ286001.1	13792 bp
8	*M. digitatus*	Nematoda	Rhabditida	Strongyloidea	Trichostrongylidae	NC_013848.1	15221 bp
9	*T. colubriformis*	Nematoda	Rhabditida	Strongyloidea	Trichostrongylidae	OZ022262.1	13693 bp
10	*S. equinus*	Nematoda	Rhabditida	Strongyloidea	Strongylidae	NC_026868.1	14545 bp
11	*O. columbianum*	Nematoda	Rhabditida	Strongyloidea	Strongylidae	NC_023933.1	13561 bp
12	*A. ceylanicum*	Nematoda	Rhabditida	Strongyloidea	Ancylostomatidae	AP017674.1	13655 bp
13	*N. americanus*	Nematoda	Rhabditida	Strongyloidea	Ancylostomatidae	NC_003416.2	13605 bp
14	*T. canis*	Nematoda	Rhabditida	Ascaridoidea	Toxocaridae	EU730761.1	14163 bp

Supplementary Table S15: Pairwise distance analysis

Supplementary Table 15. Pairwise distance analysis

Species_Accession No	1	2	3	4	5	6	7	8	9	10	11	12	13	14	15	16	17	18	19	20	21	22	23	24	25	26	27	28	29	30	31	32	33	34	35	36	37	38	39	40	41	42	43	44	45	46	47	48	49	50	51	52	53	54
Haemonchus_contortus_Pk3_04
Haemonchus_contortus_Pk3_05	0.05
Haemonchus_contortus_Pk3_06	0.05	0.02
Haemonchus_contortus_Pk4_04	0.05	0.02	0.02
Haemonchus_contortus_Pk4_05	0.02	0.04	0.04	0.04
Haemonchus_contortus_Pk4_06	0.05	0.02	0.02	0.02	0.04
Haemonchus_contortus_Pk5_05	0.05	0.02	0.02	0.03	0.05	0.02
Haemonchus_contortus_Pk5_06	0.05	0.02	0.01	0.02	0.04	0.02	0.02
Haemonchus_contortus_Pk8_03	0.05	0.02	0.02	0.02	0.04	0.02	0.02	0.02
Haemonchus_contortus_PK11_01	0.05	0.02	0.01	0.02	0.04	0.01	0.02	0.01	0.02
Haemonchus_contortus_Pk11_02	0.05	0.02	0.01	0.02	0.04	0.02	0.02	0.01	0.02	0.01
Haemonchus_contortus_Pk12_02	0.05	0.02	0.02	0.02	0.04	0.01	0.02	0.02	0.02	0.01	0.02
Haemonchus_contortus_Pk13_03	0.02	0.06	0.06	0.06	0.03	0.06	0.06	0.06	0.06	0.06	0.06	0.06
Haemonchus_contortus_Pk14_01	0.05	0.02	0.02	0.02	0.04	0.02	0.01	0.02	0.02	0.02	0.02	0.02	0.06
Haemonchus_contortus_Pk15_01	0.05	0.02	0.02	0.02	0.04	0.02	0.02	0.02	0.02	0.02	0.02	0.02	0.06	0.02
Haemonchus_contortus_Pk15_02	0.04	0.03	0.03	0.03	0.04	0.03	0.03	0.03	0.03	0.03	0.03	0.03	0.05	0.03	0.03
Haemonchus_contortus_Pk15_03	0.05	0.03	0.03	0.03	0.04	0.03	0.02	0.03	0.03	0.03	0.03	0.03	0.06	0.02	0.03	0.03
Haemonchus_contortus_Pk15_04	0.05	0.02	0.03	0.03	0.05	0.03	0.02	0.03	0.03	0.03	0.03	0.02	0.06	0.02	0.03	0.03	0.01
Haemonchus_contortus_Pk15_05	0.01	0.05	0.05	0.05	0.03	0.05	0.06	0.05	0.05	0.05	0.05	0.05	0.02	0.05	0.06	0.05	0.06	0.06
Haemonchus_contortus_Pk16_02	0.05	0.02	0.02	0.02	0.04	0.02	0.03	0.02	0.02	0.02	0.02	0.02	0.06	0.03	0.02	0.03	0.03	0.03	0.05
Haemonchus_contortus_Pk17_01	0.05	0.02	0.02	0.02	0.04	0.02	0.03	0.02	0.02	0.02	0.02	0.02	0.06	0.02	0.02	0.03	0.03	0.02	0.05	0.02
Haemonchus_contortus_Pk17_03	0.05	0.01	0.02	0.02	0.04	0.02	0.02	0.02	0.02	0.02	0.02	0.02	0.06	0.02	0.02	0.03	0.03	0.02	0.05	0.02	0.02
Haemonchus_contortus_Pk17_04	0.05	0.02	0.01	0.02	0.04	0.02	0.02	0.01	0.02	0.01	0.00	0.01	0.06	0.02	0.02	0.03	0.03	0.03	0.05	0.02	0.02	0.02
Haemonchus_contortus_Pk17_05	0.05	0.02	0.02	0.02	0.04	0.02	0.02	0.02	0.02	0.01	0.01	0.02	0.06	0.02	0.02	0.03	0.03	0.03	0.05	0.02	0.02	0.02	0.01
Haemonchus_contortus_Pk18_01	0.05	0.02	0.02	0.02	0.04	0.02	0.02	0.02	0.02	0.01	0.02	0.01	0.06	0.02	0.02	0.03	0.03	0.03	0.05	0.02	0.02	0.02	0.02	0.02
Haemonchus_contortus_Pk18_02	0.02	0.06	0.06	0.06	0.03	0.06	0.06	0.06	0.06	0.06	0.06	0.06	0.02	0.06	0.06	0.05	0.06	0.07	0.02	0.06	0.06	0.06	0.06	0.06	0.06
Haemonchus_contortus_Pk18_03	0.02	0.06	0.06	0.06	0.03	0.06	0.06	0.06	0.06	0.06	0.06	0.06	0.02	0.06	0.06	0.05	0.06	0.07	0.02	0.06	0.06	0.06	0.06	0.06	0.06	0.00
Haemonchus_contortus_Pk18_04	0.05	0.03	0.03	0.03	0.04	0.02	0.03	0.03	0.03	0.03	0.03	0.03	0.06	0.03	0.03	0.02	0.03	0.03	0.05	0.03	0.03	0.03	0.03	0.03	0.03	0.06	0.06
Haemonchus_contortus_Pk18_05	0.05	0.02	0.01	0.02	0.04	0.02	0.02	0.01	0.02	0.01	0.01	0.02	0.06	0.02	0.02	0.03	0.03	0.03	0.05	0.02	0.02	0.02	0.01	0.01	0.02	0.06	0.06	0.03
Haemonchus_contortus_Pk19_05	0.05	0.02	0.01	0.02	0.04	0.01	0.02	0.01	0.02	0.01	0.01	0.01	0.06	0.02	0.02	0.03	0.03	0.03	0.05	0.02	0.02	0.02	0.01	0.02	0.02	0.06	0.06	0.03	0.01
Haemonchus_contortus_Pk23_03	0.05	0.02	0.02	0.03	0.04	0.02	0.01	0.02	0.02	0.02	0.02	0.02	0.06	0.01	0.02	0.03	0.02	0.02	0.06	0.02	0.02	0.02	0.02	0.02	0.02	0.06	0.06	0.03	0.02	0.02
Haemonchus_contortus_Pk24_02	0.05	0.02	0.02	0.02	0.04	0.02	0.03	0.02	0.02	0.02	0.02	0.02	0.06	0.02	0.02	0.03	0.03	0.03	0.05	0.02	0.02	0.02	0.02	0.02	0.02	0.06	0.06	0.03	0.02	0.02	0.02
Haemonchus_contortus_Pk32_03	0.02	0.06	0.06	0.06	0.03	0.06	0.06	0.06	0.06	0.06	0.06	0.06	0.02	0.06	0.06	0.05	0.06	0.06	0.02	0.06	0.06	0.06	0.06	0.06	0.06	0.02	0.02	0.05	0.06	0.06	0.06	0.06
Haemonchus_contortus_Pk34_03	0.05	0.02	0.02	0.03	0.04	0.03	0.02	0.02	0.02	0.02	0.02	0.02	0.06	0.01	0.02	0.03	0.02	0.02	0.05	0.02	0.02	0.02	0.03	0.02	0.03	0.06	0.06	0.03	0.02	0.02	0.01	0.03	0.06
Haemonchus_contortus_Pk41_02	0.05	0.02	0.02	0.02	0.04	0.02	0.03	0.02	0.02	0.02	0.02	0.02	0.06	0.02	0.02	0.03	0.03	0.03	0.05	0.01	0.02	0.02	0.02	0.02	0.02	0.06	0.06	0.03	0.02	0.02	0.03	0.02	0.06	0.03
Haemonchus_contortus_Pk41_03	0.05	0.02	0.03	0.03	0.04	0.02	0.02	0.02	0.03	0.02	0.02	0.02	0.06	0.01	0.03	0.03	0.02	0.02	0.05	0.02	0.02	0.02	0.02	0.03	0.02	0.06	0.06	0.03	0.03	0.02	0.02	0.03	0.06	0.01	0.03
Haemonchus_contortus_Pk45_01	0.05	0.02	0.02	0.02	0.04	0.02	0.03	0.02	0.02	0.02	0.02	0.02	0.06	0.03	0.02	0.03	0.03	0.03	0.05	0.02	0.02	0.02	0.02	0.02	0.02	0.06	0.06	0.03	0.02	0.02	0.03	0.02	0.06	0.03	0.02	0.03
Haemonchus_contortus_Pk47_01	0.05	0.01	0.02	0.02	0.04	0.02	0.02	0.02	0.02	0.02	0.02	0.02	0.06	0.02	0.02	0.02	0.03	0.02	0.05	0.02	0.02	0.01	0.02	0.02	0.02	0.06	0.06	0.03	0.02	0.02	0.02	0.02	0.06	0.02	0.02	0.02	0.02
Haemonchus_contortus_Pk47_03	0.05	0.03	0.03	0.03	0.04	0.03	0.03	0.03	0.03	0.03	0.03	0.03	0.05	0.03	0.03	0.03	0.03	0.03	0.05	0.03	0.03	0.03	0.03	0.03	0.03	0.06	0.06	0.02	0.03	0.03	0.03	0.03	0.05	0.03	0.03	0.03	0.03	0.03
Haemonchus_contortus_Pk48_01	0.05	0.02	0.02	0.02	0.04	0.02	0.02	0.02	0.02	0.02	0.02	0.02	0.06	0.02	0.02	0.03	0.03	0.02	0.05	0.02	0.02	0.01	0.02	0.02	0.02	0.06	0.06	0.03	0.02	0.02	0.02	0.02	0.06	0.02	0.02	0.03	0.03	0.02	0.03
Haemonchus_contortus_Pk48_02	0.05	0.02	0.02	0.02	0.04	0.02	0.02	0.02	0.02	0.02	0.02	0.02	0.06	0.02	0.02	0.03	0.03	0.02	0.05	0.02	0.02	0.01	0.02	0.02	0.02	0.06	0.06	0.03	0.02	0.02	0.02	0.02	0.06	0.02	0.02	0.03	0.03	0.02	0.03	0.00
Haemonchus_contortus_LS997568.1	0.02	0.06	0.06	0.06	0.03	0.06	0.06	0.06	0.06	0.06	0.06	0.06	0.02	0.06	0.06	0.05	0.06	0.06	0.02	0.06	0.06	0.06	0.06	0.06	0.06	0.01	0.01	0.06	0.06	0.06	0.06	0.06	0.02	0.06	0.06	0.06	0.06	0.06	0.06	0.06	0.06
Haemonchus_contortus_NC_010383.2	0.02	0.06	0.06	0.06	0.03	0.06	0.06	0.06	0.06	0.06	0.06	0.06	0.03	0.06	0.06	0.06	0.06	0.07	0.02	0.06	0.06	0.06	0.06	0.06	0.06	0.02	0.02	0.06	0.06	0.06	0.06	0.06	0.02	0.06	0.06	0.06	0.06	0.06	0.05	0.06	0.06	0.02
Haemonchus_contortus_CP149429.1	0.01	0.06	0.06	0.06	0.02	0.06	0.06	0.06	0.06	0.06	0.06	0.06	0.02	0.06	0.06	0.05	0.06	0.06	0.01	0.06	0.06	0.06	0.06	0.06	0.06	0.02	0.02	0.05	0.06	0.06	0.06	0.06	0.02	0.06	0.06	0.06	0.06	0.06	0.05	0.06	0.06	0.02	0.02
Haemonchus_contortus_CP035799.1	0.01	0.06	0.06	0.06	0.02	0.06	0.06	0.06	0.06	0.06	0.06	0.06	0.02	0.06	0.06	0.05	0.06	0.06	0.01	0.06	0.06	0.06	0.06	0.06	0.06	0.02	0.02	0.05	0.06	0.06	0.06	0.06	0.02	0.06	0.06	0.06	0.06	0.06	0.05	0.06	0.06	0.02	0.02	0.00
Haemonchus_placei_AP017687.1	0.16	0.16	0.16	0.16	0.16	0.16	0.16	0.15	0.15	0.16	0.16	0.16	0.16	0.16	0.16	0.15	0.16	0.16	0.16	0.16	0.16	0.16	0.16	0.16	0.16	0.16	0.16	0.16	0.16	0.16	0.16	0.16	0.16	0.15	0.16	0.16	0.16	0.16	0.15	0.16	0.16	0.16	0.17	0.16	0.16
Haemonchus_placei_NC_029736.1	0.16	0.16	0.16	0.16	0.16	0.16	0.16	0.16	0.16	0.16	0.16	0.16	0.16	0.16	0.16	0.16	0.16	0.16	0.16	0.16	0.16	0.16	0.16	0.16	0.16	0.16	0.16	0.16	0.16	0.16	0.16	0.16	0.16	0.16	0.16	0.16	0.16	0.16	0.15	0.16	0.16	0.16	0.17	0.16	0.16	0.00
Mecistocirrus_digitatus_NC_013848.1	0.37	0.39	0.37	0.39	0.37	0.38	0.38	0.38	0.38	0.38	0.38	0.38	0.38	0.38	0.39	0.39	0.39	0.39	0.37	0.39	0.39	0.39	0.38	0.38	0.39	0.38	0.38	0.38	0.38	0.37	0.39	0.39	0.37	0.39	0.38	0.39	0.38	0.39	0.38	0.40	0.40	0.37	0.37	0.37	0.37	0.37	0.37
Cooperia_oncophora_OZ286001.1	0.52	0.52	0.52	0.53	0.53	0.51	0.52	0.50	0.51	0.51	0.52	0.52	0.53	0.51	0.52	0.50	0.51	0.51	0.52	0.53	0.53	0.52	0.52	0.51	0.52	0.52	0.52	0.53	0.51	0.52	0.52	0.52	0.51	0.52	0.52	0.51	0.52	0.52	0.51	0.52	0.52	0.51	0.53	0.52	0.52	0.51	0.51	0.44
Strongylus_equinus_NC_026868.1	0.56	0.58	0.57	0.57	0.56	0.57	0.56	0.56	0.56	0.57	0.56	0.58	0.57	0.57	0.58	0.56	0.57	0.56	0.56	0.56	0.58	0.56	0.55	0.57	0.58	0.56	0.56	0.56	0.57	0.56	0.57	0.57	0.57	0.57	0.57	0.58	0.57	0.57	0.54	0.57	0.57	0.55	0.58	0.57	0.57	0.56	0.57	0.55	0.44
Trichostrongylus_colubriformis_OZ022262.1	0.52	0.52	0.50	0.51	0.51	0.51	0.52	0.50	0.50	0.51	0.50	0.52	0.52	0.52	0.53	0.52	0.52	0.53	0.51	0.51	0.51	0.51	0.50	0.50	0.52	0.52	0.52	0.52	0.50	0.51	0.51	0.52	0.53	0.51	0.50	0.52	0.50	0.51	0.50	0.51	0.51	0.53	0.52	0.51	0.51	0.48	0.49	0.56	0.44	0.51
Oesophagostomum_columbianum_NC_023933.1	0.54	0.55	0.54	0.55	0.54	0.55	0.55	0.53	0.55	0.54	0.55	0.55	0.55	0.54	0.56	0.55	0.54	0.54	0.54	0.55	0.55	0.55	0.54	0.55	0.56	0.55	0.55	0.53	0.55	0.54	0.54	0.54	0.54	0.54	0.55	0.54	0.55	0.55	0.52	0.54	0.54	0.54	0.55	0.55	0.55	0.46	0.47	0.48	0.39	0.19	0.47
Ancylostoma_ceylanicum_AP017674.1	0.49	0.49	0.49	0.51	0.49	0.50	0.50	0.50	0.49	0.50	0.49	0.51	0.50	0.49	0.51	0.49	0.50	0.50	0.49	0.50	0.50	0.49	0.49	0.50	0.51	0.49	0.49	0.50	0.50	0.50	0.50	0.49	0.50	0.49	0.50	0.51	0.49	0.50	0.49	0.50	0.50	0.49	0.50	0.50	0.50	0.45	0.45	0.42	0.35	0.18	0.42	0.17
Necator_americanus_NC_003416.2	0.60	0.58	0.58	0.60	0.59	0.60	0.59	0.59	0.58	0.59	0.60	0.60	0.59	0.58	0.61	0.60	0.58	0.59	0.61	0.60	0.59	0.59	0.60	0.59	0.60	0.61	0.61	0.59	0.59	0.61	0.58	0.59	0.60	0.58	0.60	0.59	0.58	0.61	0.57	0.59	0.59	0.60	0.63	0.60	0.60	0.54	0.55	0.55	0.44	0.25	0.53	0.23	0.22
Toxocara_canis_EU730761.1	1.39	1.37	1.36	1.38	1.37	1.39	1.37	1.36	1.36	1.37	1.35	1.37	1.40	1.37	1.38	1.39	1.36	1.36	1.39	1.36	1.38	1.37	1.35	1.38	1.38	1.39	1.39	1.38	1.36	1.36	1.36	1.36	1.39	1.37	1.38	1.37	1.39	1.39	1.35	1.36	1.36	1.39	1.39	1.38	1.38	1.28	1.28	1.53	1.29	1.12	1.41	1.10	1.05	0.94

The number of base substitutions per site from between sequences are shown. Analyses were conducted using the Maximum Composite Likelihood model. The analytical procedure encompassed 55 coding nucleotide sequences. The complete deletion option was applied to eliminate positions containing gaps and missing data

Supplementary Figure S1

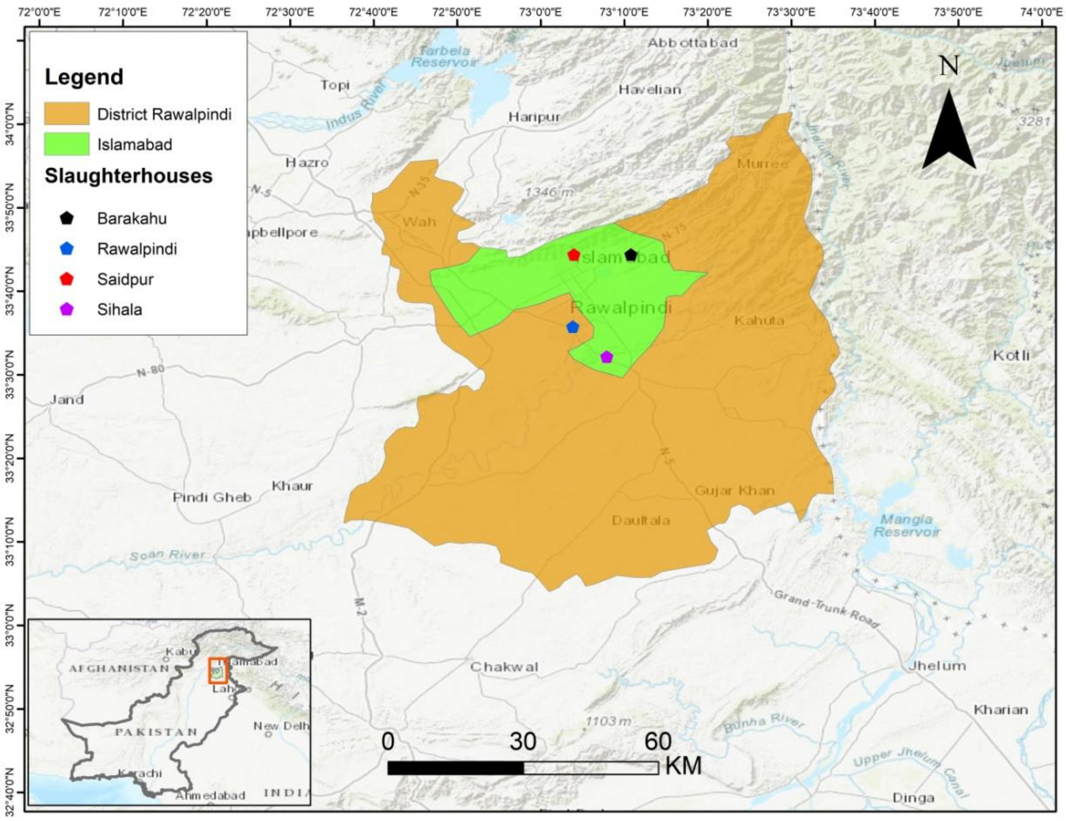

Supplementary Figure S1: Map of study area indicating four slaughterhouses in Rawalpindi district and adjoining areas, Pakistan.

Supplementary Figure S2.

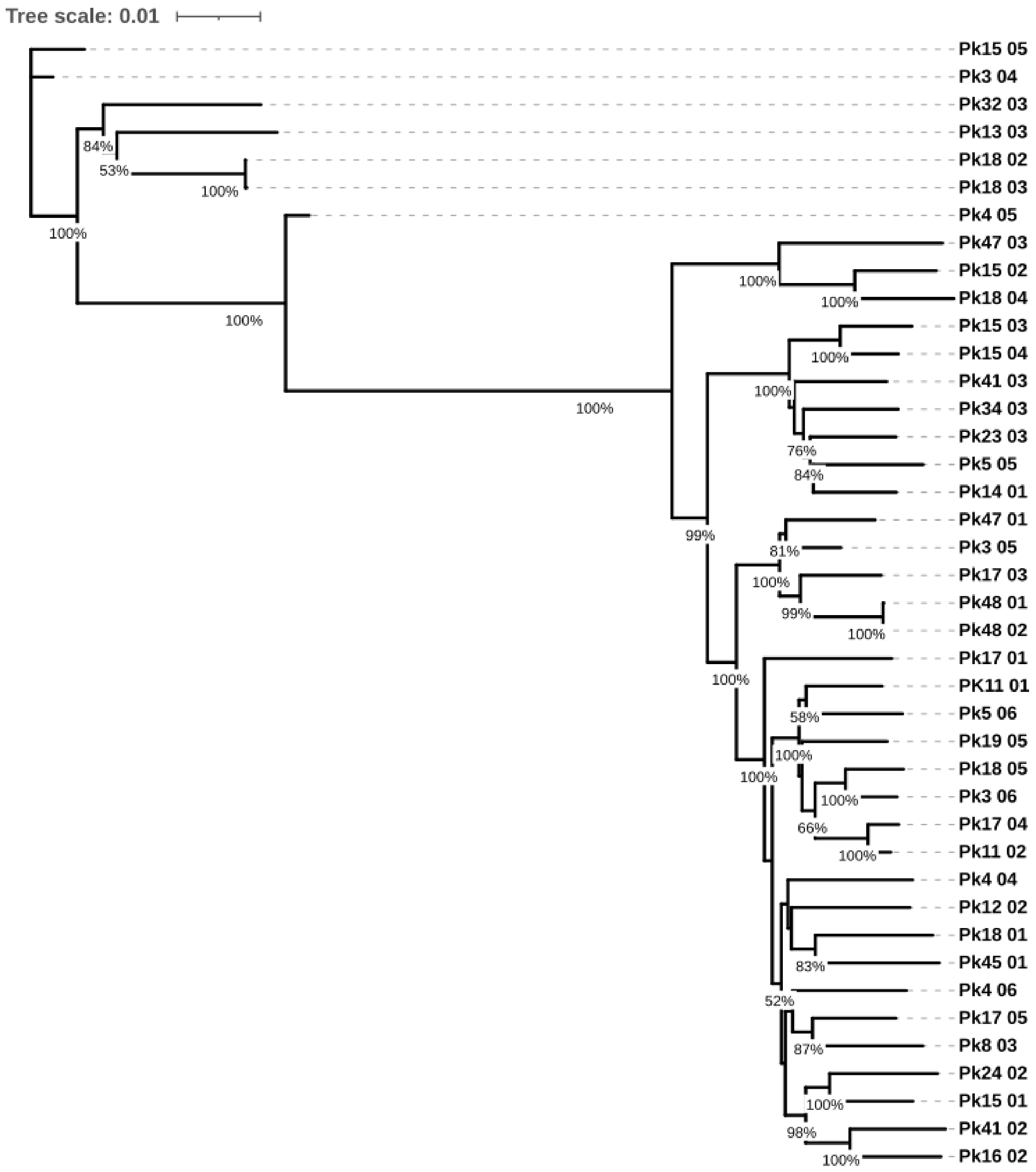

Supplementary Figure S2: Maximum likelihood tree of present study *H. contortus* isolates.
